# GWIS - model-free, fast and exhaustive search for epistatic interactions in case-control GWAS

**DOI:** 10.1186/1471-2164-14-S3-S10

**Published:** 2013-05-28

**Authors:** Benjamin Goudey, David Rawlinson, Qiao Wang, Fan Shi, Herman Ferra, Richard M Campbell, Linda Stern, Michael T Inouye, Cheng Soon Ong, Adam Kowalczyk

**Affiliations:** 1 National ICT Australia Victorian Research Lab, The University of Melbourne, Parkville, Victoria, Australia; 2 Computing and Information Systems, The University of Melbourne, Parkville, Victoria, Australia; 3 Electrical and Electronic Engineering, The University of Melbourne, Parkville, Victoria, Australia; 4 Pathology, The University of Melbourne, Parkville, Victoria, Australia; 5 Microbiology & Immunology, The University of Melbourne, Parkville, Victoria, Australia

## Abstract

**Background:**

It has been hypothesized that multivariate analysis and systematic detection of epistatic interactions between explanatory genotyping variables may help resolve the problem of "missing heritability" currently observed in genome-wide association studies (GWAS). However, even the simplest bivariate analysis is still held back by significant statistical and computational challenges that are often addressed by reducing the set of analysed markers. Theoretically, it has been shown that combinations of loci may exist that show weak or no effects individually, but show significant (even complete) explanatory power over phenotype when combined. Reducing the set of analysed SNPs before bivariate analysis could easily omit such critical loci.

**Results:**

We have developed an exhaustive bivariate GWAS analysis methodology that yields a manageable subset of candidate marker pairs for subsequent analysis using other, often more computationally expensive techniques. Our model-free filtering approach is based on classification using ROC curve analysis, an alternative to much slower regression-based modelling techniques. Exhaustive analysis of studies containing approximately 450,000 SNPs and 5,000 samples requires only 2 hours using a desktop CPU or 13 minutes using a GPU (Graphics Processing Unit). We validate our methodology with analysis of simulated datasets as well as the seven Wellcome Trust Case-Control Consortium datasets that represent a wide range of real life GWAS challenges. We have identified SNP pairs that have considerably stronger association with disease than their individual component SNPs that often show negligible effect univariately. When compared against previously reported results in the literature, our methods re-detect most significant SNP-pairs and additionally detect many pairs absent from the literature that show strong association with disease. The high overlap suggests that our fast analysis could substitute for some slower alternatives.

**Conclusions:**

We demonstrate that the proposed methodology is robust, fast and capable of exhaustive search for epistatic interactions using a standard desktop computer. First, our implementation is significantly faster than timings for comparable algorithms reported in the literature, especially as our method allows simultaneous use of multiple statistical filters with low computing time overhead. Second, for some diseases, we have identified hundreds of SNP pairs that pass formal multiple test (Bonferroni) correction and could form a rich source of hypotheses for follow-up analysis.

**Availability:**

A web-based version of the software used for this analysis is available at http://bioinformatics.research.nicta.com.au/gwis.

## Background

Genome-wide association studies (GWAS) have discovered many underlying genetic causes of disease, but have also raised important questions about standard approaches to modelling complex traits [1]. While commonly-used univariate analysis techniques have been able to detect a number of significantly associated loci, for many conditions these discovered variants do not account for a majority of the theoretical estimates of genetic heritability. Multivariate approaches may help to alleviate this issue of "missing heritability" [2]. Theoretically, it has been shown that 2-way and 3-way single nucleotide polymorphism (SNP) interactions can explain up to ~ 50% and ~ 100% of trait variance while each SNP involved explains none [3], indicating that critical SNP pairs may be ignored by univariate analysis predominantly applied to GWAS so far. It is hypothesised that systematic detection methods may assist discovery of such potentially epistatic interactions between DNA loci.

### Motivation

To date there exists little experimentally-validated evidence of SNP interactions in humans, largely due to the complexity of multivariate GWAS analysis. Even in only bivariate analysis, the number of possible SNP interactions that need to be searched is extremely large, as there are 125 billion possible SNP pairs in a GWAS of 500,000 SNPs. The scale of the problem produces significant computational and statistical challenges. Numerous approaches proposed to address these challenges are unable to scale to this large number of tests, due to both performance and accuracy (a large number of false-positive results are expected from so many tests, generating concerns about the effectiveness of multiple-test correction). This has led to claims that finding epistatic interactions via exhaustive search is infeasible [4,5]. While these pessimistic claims have recently been proven wrong (e.g. [6-10]), techniques that do scale to exhaustive search currently require weeks or months to process GWAS of 5 million SNPs, which are becoming increasingly common. As GWAS studies continue to grow in size, faster analysis techniques will be needed. This paper aims to offer solutions that meet these ever-increasing requirements.

### Epistatic interactions

Our goal is to present a system capable of exhaustive search through all SNP pairs in an entire GWAS, detecting all *significant epistatic interactions*. As discussed in [11], both the terms "significant" and "epistatic interaction" have diverse definitions when used by biologists, epidemiologists, statisticians and geneticists and are often not made explicit. We specify the precise meanings of these terms as used in this paper, presenting a verbal description now and a more specific elaboration in the Methods section. We say that two SNPs have an *epistatic interaction *if using both of them allows discrimination between Cases and Controls with significantly higher sensitivity and specificity than is possible using any one of them individually. The *significance *is quantified as a p-value for rejection of a well specified null hypothesis (see Methods for details). This rejection implies in particular, that the improvement cannot be explained by biased sampling from a population pre-classified by any one of the SNPs in the pair. In the Discussion section we argue that our generic formal definition of epistasis captures some biological aspects of epistasis that Fisher's popular definition of interaction [12] misses.

### GWIS approach

The definitions given above can be directly converted into computational methods, suitable for scanning trillions of SNP pairs in a modern GWAS and providing an alternative to widely-used regression based approaches. In this work, we present a platform called Genome Wide Interaction Search (GWIS), that is based on classification, and novel rigorous statistical tests based on receiver operating characteristic (ROC) curve analysis [13]. Our proposed method is genuinely "model free", since we do not assume any interaction model between SNP genotypes. In this sense we are close to other model free approaches, in particular Multifactor Dimensionality Reduction (MDR) [14-16], although we rely on analytical solutions to hypothesis-based testing rather than slower, computationally-costly cross-validation and permutation testing.

We demonstrate that exhaustive search of all possible pairs in standard GWAS is feasible and fast on a desktop computer and that our proposed technique is faster than currently available exhaustive techniques. Aside from the computational challenges mentioned above, there are a number of statistical challenges that also need to be resolved. Principled methods are needed that allow for significance-correction of the billions of SNP-pair and genotype combinations, and that are able to cope with characteristics of real-world data, e.g. confounding factors due to strong univariate signals, examining significance in the far tail of distributions where the central limit approximation does not hold, and SNPs with low minor allele frequency giving rise to very low genotype counts.

We introduce a novel and theoretically well-founded, model-free hypothesis test specifically designed for multivariate GWAS analysis. It is based on relating the sensitivity and specificity observed in the sampled data to the sensitivity and specificity that could be achieved in the 'true' population. The test, named *gain in sensitivity and specificity *(GSS), is designed to detect epistatic SNP interactions, and computes exact p-values, without using large sample normal approximations. Each application of the GSS test to a pair of SNPs involves solving a number of min-max optimisations, which are pair specific and are therefore impractical for scanning trillions of putative SNP-pairs. Hence, we introduce two extra tests, referred to as *sensitivity and specificity *(SS) and *difference in sensitivity and specificity *(DSS), that act as practical fast proxies for the GSS test.

### Validation

Algorithms for detecting epistatic SNP interactions are typically evaluated using simulated data, for reasons of both scalability and interpretation [17-19]. However, the creation of realistic structure in simulated data is problematic as much is unknown about the nature and existence of epistasis in humans [20,21]. Therefore, we primarily focus on seven GWAS datasets from the Wellcome Trust Case-Control Consortium (WTCCC) [22]. These data include various real GWAS challenges that are not always represented in simulated data. Although the set of true SNP interactions is not yet known for WTCCC data, analysis of this data using multiple types of analysis provides evidence on the properties of the epistatic interactions that can be observed, reveals confounding factors not generally modelled in synthetic data, and demonstrates the advantages and limitations of different statistical filtering approaches. The efficiency of our methods is demonstrated by comparing timings of our methods on various size datasets to those reported in several recent publications. The proposed statistical filters are further benchmarked by confirming their theoretically advantageous properties and validation of their power and false positive rates over an extensive collection of synthetic datasets available from [23]. We show the importance of exhaustive search without which heuristics may miss significant SNP pairs. We demonstrate that our GSS test is able to identify a number of interesting SNP pairs that show significant epistatic effects. Detected results are compared to those from existing literature, showing that GWIS repeats many known results, as well as suggesting many novel interactions.

### Contributions

This paper makes several contributions. First, we use an operational definition of epistasis based on classification of individuals into Cases or Controls to develop a set of robust, principled methods for explicitly detecting significant epistatic interactions in GWAS data. Second, we demonstrate that our proposed methods scale well and are fast enough to permit exhaustive analysis of current and near-future GWAS data. Third, we have applied GWIS to a diverse range of both simulated and real life benchmark data, and detected many significant associations in addition to confirming many associations previously reported. Finally, our analysis of real data indicates the limitations of conventional statistical methods such as Pearson's $\chi^{2}$ test for detecting epistatic interactions in the presence of strong main effects.

## Results

An exhaustive evaluation of all possible SNP pairings is the most powerful strategy to detect epistatic interactions [24] but to date remains a computationally challenging task. Most methods have been unable to scale exhaustive methods to entire GWAS without performing some reduction in the number of pairs to be evaluated [5], or requiring special hardware such as a compute cluster [25-27].

### Comparison of computation time

GWIS is able to exhaustively search whole GWAS on a desktop PC with no special hardware, and can also take advantage of available retail Graphics Processing Units (GPUs) to further reduce execution time. The implementation of GWIS allows multiple filters to simultaneously evaluate SNP pairs with low impact on speed. Table 1 shows runtime for GWIS using CPU and GPU implementations, applying either 1 or 3 statistical filters. For comparison, we show timing reported by other recent SNP interaction detection methods, both CPU and GPU, scaled to 450K and 5M SNP arrays using the formulas reported in the Supplement Section 2, "Calculation of Timing". Timing data for GWIS was acquired using a 4-core, 64 bit, 3 GHz Intel CPU and an NVIDIA GTX 470 graphics card (GPU). We converted the timing results reported in literature to the above platform. Exact comparison with other results is problematic because different hardware was used, but the dramatic improvements in runtime cannot be attributed to hardware choice alone.

**Table 1:** Runtime required for GWIS compared to recent CPU and GPU methods.

Method	Time for *n_SNP _*× *n_samples_*		Exhaustive Search
	0.45M × 5K	5M × 10K	
**CPU Implementation:**			
**(4 cores, 64 bits, 3GHz Intel)**			

GWIS (1 filter)	2.7 hours*	28 days	Yes
GWIS (3 filters)	10.9 hours*	113 days	Yes
BOOST [31]	23 hours	8 months	Yes
PLINK [53]	89 days	60 years	Yes

RAPID [32]	15 mins	NA	No
SIXPAC [24]	8.0 hours	NA	No

**GPU Implementation:**			
(448 CUDA cores, 1.215 GHz, NVIDIA GTX 470)			

GWIS (1 filter)	13 mins*	2.2 days	Yes
GWIS (3 Filters)	22 mins*	3.8 days	Yes
GBOOST [7]	1.4 hours	15 days	Yes
EpiGPU [8]	17 hours	6 months	Yes
SHEsisEPI [9]	28 hours	10 months	Yes
EPIBLASTER [6]	8.9 days	6 years	Yes

Runtime required for GWIS compared to recent CPU and GPU methods. The first column reports results for WTCCC-sized data (450K SNPs, 5K samples) while the second column shows timings for larger recent GWAS (5M SNPs, 10K samples). The rightmost column denotes whether a method performs exhaustive search of all pairs. Filters run by GWIS are $\chi^{2}$ alone or in combination with DSS and SS tests. The times indicated by '*' are actual run times, and all other times are estimated by scaling (see Supplement Section 2 for exact calculations). Runtime for non-exhaustive methods has not been estimated as it is difficult to judge how these methods scale with the number of SNPs and samples.

Table 1 demonstrates that exhaustive evaluation of all possible SNP pairs is feasible on a standard desktop machine with GWIS taking 2.7 hours for CPU and 13 minutes for GPU implementations. This represents an approximate 9× and 6× speed up over other alternative CPU and GPU exhaustive-search methods respectively, and is faster than many methods that use heuristic search strategies. The only faster method reported here is a non-exhaustive search algorithm RAPID, whose timing reported here excludes parameter tuning that increases the actual time dramatically and has profound impact on performance (see the following Section).

For GWIS, we report runtime using one filter and three filters, namely $\chi^{2}$ alone or in combination with DSS and SS tests. The latter two tests are more computationally intensive than most existing statistical filters such as $\chi^{2}$, Difference of Odds (DoO) and the Fisher Exact test (FE). Approximately 60% of the runtime for $\chi^{2}$ alone is spent computing contingency tables, that are subsequently used by all statistical tests. On the reference machine used for CPU results in Table 1, $\chi^{2}$ alone runs in 2.7 hours. $\chi^{2}$, DoO and FE can be completed in 4.6 hours. $\chi^{2}$, DSS and SS requires 10.9 hours.

If we consider arrays of 5M SNPs, the estimated difference in times shows the necessity of faster exhaustive methods. Many algorithms that had acceptable runtime on current size GWAS will take weeks or months to compute on the larger number of SNPs as the total number of pairs to be evaluated grows quadratically. While the CPU implementation of GWIS would require about 3 months, the GPU implementation requires 3 days, a feasible wait for research results. Both CPU and GPU implementations could be deployed on a computing cluster to easily reduce this runtime down to a few minutes.

We expected the runtime of our methods to increase linearly with the number of samples and quadratically with increasing SNPs (i.e. linear in terms of SNP-pairs). To verify this, we examined program runtime on simulated datasets varying both the number of samples and the number of SNPs. These datasets contained between 125K and 1M SNPs and between 1250 and 10K samples. Due to the independence of computations on each SNP-pair, both CPU and GPU implementations show the expected relationships between samples, SNPs and runtime. Note that actual timings will be affected by machine architecture; in addition to obvious factors such as clock speed, we exploit low-level functions that are found in most modern CPUs. Older CPUs without high performance functions will not execute GWIS as quickly.

### Summary and analysis of interactions detected using different statistical filters

The efficiency of GWIS enables exhaustive pairwise analysis of multiple studies using multiple statistical filters. We present an initial analysis of the seven WTCCC datasets listed in Table 2 and explore the detected pairs arising from two statistical tests, $\chi^{2}$ and DSS, implemented in GWIS. $\chi^{2}$ is a standard hypothesis test for association [28] that has been used in numerous interaction detection methods [25,29,30] but its effectiveness has been generally evaluated over simulated rather than real data. DSS is a novel filter that explicitly searches for pairs that show a more significant association with phenotype than either of the two SNPs individually (details in the Methods section). For comparison, we also evaluated GBOOST [7], a GPU method based on the earlier BOOST method [10,31] and which represents the current state of the art for epistasis detection [19]. Table 3 reports the number of SNP pairs detected using each method that show significant association where significance is defined by Bonferroni correction $\left(\text{p - value}={\left(\begin{array}{c} 459,012\\ 2 \end{array}\right)}^{-1}\approx 10^{-11}\right)$. GBOOST was run using default parameters. For some datasets, a univariate analysis using $\chi^{2}$ detected extremely strong associations. These p-values reported here and for corresponding plots in supplementary material are likely due to associations driven by the HLA region which have been previously reported [22].

**Table 2:** Abbreviations and number of Case samples for each WTCCC dataset.

Abbreviation	Num. of Cases	Disease
BD	1868	Bipolar Disorder
CAD	1926	Coronary Artery Disease
CD	1748	Crohn's Disease
HT	1952	Hypertension
RA	1860	Rheumatoid Arthritis
T1D	1963	Type 1 Diabetes
T2D	1924	Type 2 Diabetes

Abbreviations and number of Case samples for each WTCCC dataset [22]. All datasets have 459,012 SNPs and share 2983 Control samples.

**Table 3:** Summary of the number of SNP pairs detected by different filtering methods.

Univariate			Bivariate Filter				Bivariate Filter + GSS			
**Dataset**	**log_10 _**$P_{\chi^{2}}$		$\chi^{2}$		**DSS**	**GBOOST**		$\chi^{2}$	**DSS**	**GBOOST**

HT	-9.8		128		429	51		41	107	24
BD	-10.9		2445		556	34		44	179	27
CAD	-13.1		210147		7807	43		42	116	39
T2D	-13.3		56592		3105	52		79	134	41
CD	-34.3		> 500000^∗^		5591	25		29	57	22
RA	-37.7		> 500000^∗^		823	99		59	312	95
T1D	-133.6		> 500000^∗^		4993	37		2	107	33

Summary of the number of SNP pairs detected by $\chi^{2}$, GBOOST and our introduced DSS heuristic over all WTCCC datasets before and after filtering with GSS. The rows of the table are sorted in descending order of p-values for univariate $\chi^{2}$ test (Column 2). Columns 3-5 show results for the bivariate filters, and columns 6-8 show the number of epistatic interactions discovered after further filtering with GSS. In some diseases, strong univariate SNPs likely cause proliferation of non-epistatic but significant pairs according to $\chi^{2}$. These pairs are largely removed by the proposed GSS filter. A '*' indicates that an upper bound on the number of recorded pairs was reached. The number of significant pairs may be much higher.

We found that the evaluated methods varied greatly in the number of interactions detected. $\chi^{2}$ reported many interactions that passed Bonferroni correction, totalling many hundreds of thousands of SNP pairs in some datasets. This suggests additional filtering is required. GBOOST was also able to detect a number of SNP pairs with significant association in all datasets, though this is reduced compared with previously reported results and is less than we report using our novel DSS test. We also attempted to run RAPID [32], which is based on a geometric approximation to $\chi^{2}$, but despite a lengthy parameter tuning stage, requiring multiple iterations over the WTCCC data, we were unable to detect any significant SNPs in real data. These differences in results with previous reports for GBOOST and RAPID may be caused by varying quality control measures, or parameter settings.

The vast number of positive results that a conventional $\chi^{2}$ statistic generates for some datasets appears to be associated with the strength of univariate SNP association seen in the data. We hypothesise that SNPs showing strong univariate association may have a possible confounding effect. If a strongly associated SNP is paired with a SNP showing no association, the resulting pair is likely to have at least the same level of association according to $\chi^{2}$ as the strongest of the two. Given the vast number of pairs being examined, it is likely that such "univariately-driven" pairs overwhelm the results and reduce the ranking of SNP pairs with "genuine" epistatic effects enough that they are impossible to recover using post-processing techniques.

**Figure 1:**
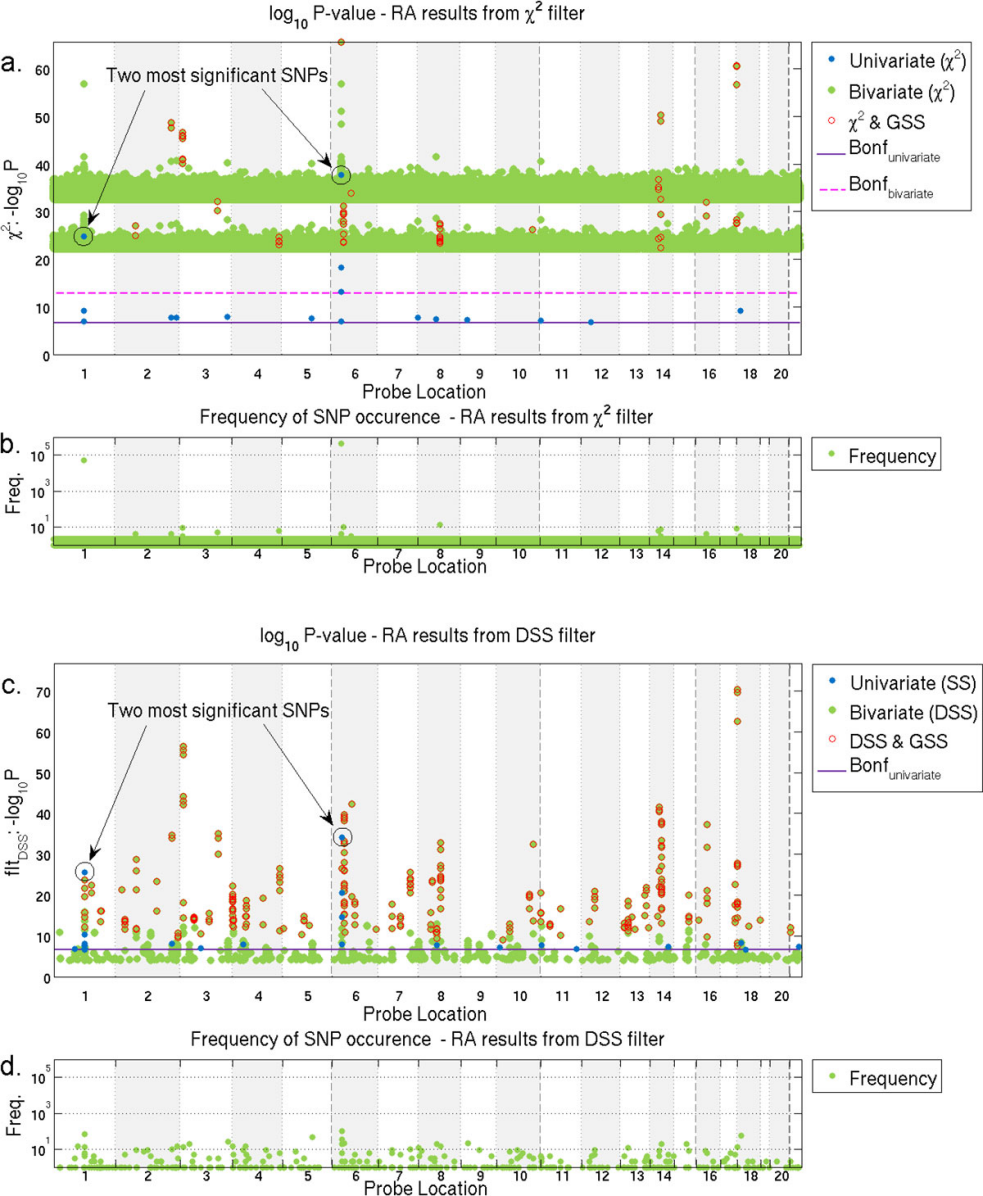
**Strength of SNPs individually and as pairs, and the frequency of SNPs appearing in pairs detected in RA by **$\chi^{2}$**and DSS**. Manhattan plots for univariate and bivariate SNP and the frequency of SNPs occurrence in pairs detected in RA by $\chi^{2}$ and DSS. The Manhattan plots (a, c) show location and p-values of univariately significant SNPs (blue) and bivariately significant SNP pairs (green). Additionally, we mark the subset of SNP pairs that are also significant according to GSS (red circle). Each SNP pair generates two points. The frequency plots (b, d) show the number of reported pairs that each SNP appears in. The Manhattan plot for $\chi^{2}$ (a) indicates almost all reported pairs appear in two distinct bands across the genome. The frequency plot (b) indicates these pairs all involve one of the two most significant SNPs from univariate analysis (highlighted) and therefore majority of them are unlikely to be epistatic. Manhattan plot (c) shows the DSS filter eliminates the banding pattern seen for $\chi^{2}$ and the frequency plot (d) shows that a greater number of unique SNPs are present in detected pairs. Note in Manhattan plot (c), the p-values for univariate association are from SS test as DSS only applies to pairs. There is also no pairwise Bonferroni line shown because DSS is a heuristic rather than a calibrated p-value.

Figure 1 further investigates this effect in detail, showing the strength of significant univariate and pairwise association detected by $\chi^{2}$ in the RA dataset. Univariate analysis reveals a strong signal coming from chromosome 6 within the HLA region, a known risk area for RA and many other diseases. In Figure 1(a) we see two bands of SNP pairs across the entire genome. The significance of association for SNP pairs in the upper and lower bands correspond closely to the association of the most and second-most significant SNPs on chromosome 6 and 1 respectively.

In Figure 1(b) we plot the number of times that each SNP occurs in the list of top pairs reported by $\chi^{2}$. While most SNPs occur in fewer than 10 pairs, the two outliers correspond to the two SNPs with strongest univariate significance indicating they occur in 99% of the 500,000 top-ranked SNP pairs reported by $\chi^{2}$. The majority of these SNP pairs are therefore unlikely to be evidence of epistatic interactions as their perceived association is due to univariate effects only. When used for the detection of epistatic SNP pairs, the $\chi^{2}$ statistic tests only for an association with phenotype but, unfortunately, fails to adequately take into account whether this association is due to univariate effects only. In the search for epistatic interactions, such pairs represent a source of noise that can cause practical problems for many standard tests of association.

### Novel statistics to account for strong univariate effects

The confounding by strong univariate signals similar to the results of $\chi^{2}$ filtering in Figure 1(a) has been seen elsewhere [26,27,33], but previously proposed methods of accounting for these effects are either heuristic (difficult to interpret and lacking in statistical rigour), or are based on regression (requiring slower iterative solutions and assumptions about the way in which SNPs interact). Here we present the results of our novel GSS test as an alternative solution for dealing with these effects.

In Table 3, we indicate the number of pairs detected by $\chi^{2}$ that are significant according to the GSS test given the conservative Bonferroni threshold of significance. The number of significant pairs falls dramatically for diseases with strong univariate signals, from hundreds of thousands down to tens. These reductions support our hypothesis that most of the SNP pairs detected by the $\chi^{2}$ filter show very weak or no epistatic effect.

Interestingly, repeating the same approach over the pairs detected by GBOOST removes very few pairs for most datasets. This is likely because GBOOST looks for significant interactions by examining the improvement of fit in log-linear regression models with and without an interaction term, in essence searching for SNP pairs with no strong univariate effects. The downside of such a technique is that a number of assumptions must be made, in this case requiring that the epistatic SNP pair must fall under an additive model. Such assumptions are not made by the GSS test.

Current implementations of the GSS statistic are too computationally expensive to use on all possible SNP pairs but can easily be run over a few million candidate pairs (our MATLAB implementation requires ≈ 90 minutes for evaluation of 1 million SNP pairs, see Additional File 1 Section 1.6). We therefore take a two-stage filtering approach similar to many other methods [6,10,29,32,34], running a fast but lenient primary filter exhaustively over all pairs, followed by the slower but more accurate GSS test on the smaller subset of pairs selected by the initial filter. As a primary filter, we could use $\chi^{2}$, though the proliferation of strong univariate SNPs is often so large that it is not feasible to store all significant pairs within a ranked list. As an alternative primary filter, we introduce the DSS, based on similar concepts to the GSS statistic. The DSS test measures the log-p-value difference between a pair of SNPs and the strongest individual SNP in the pair. This approach is similar to that used in [26,27], and is well correlated with the GSS test (see Additional File Figures 3 and 4) but is much faster to compute.

To demonstrate the effectiveness of the DSS heuristic followed by the GSS filter, we repeat the same analysis as performed for $\chi^{2}$ and GBOOST. Table 3 shows that DSS detects hundreds or thousands of SNP pairs in all datasets and after filtering using the GSS statistic, there are more SNP pairs remaining than for either $\chi^{2}$ or GBOOST, in every dataset, respectively.

In Figure 1(c), we plot the significance of SNP pairs chosen by the DSS filter. The figure demonstrates that the DSS heuristic largely addresses the proliferation of SNP pairs caused by strong univariate SNPs, with the chosen SNP pairs no longer showing the similar banding effect seen in the corresponding plot for $\chi^{2}$ shown in Figure 1(a). The frequency plot (Figure 1(d)) further demonstrates this, indicating that while some SNPs appear more frequently than others, no single SNP dominates the entire list. The SNP pairs with high DSS show an improved concordance with GSS compared to the concordance seen for $\chi^{2}$ in Figure 1(a).

### Comparison to previously reported interactions

The WTCCC datasets have been thoroughly examined by a number of epistasis detection methods many of which have reported significantly interacting SNP pairs, including some with evidence of replication of association in other datasets. We have conducted a comparison of these previous results [26,35-37] with the SNP pairs reported by GWIS using a combination of DSS and GSS filters.

Each study reported in the literature uses its own statistics for determining a pair's significance and while direct comparison between p-values from these statistics is not meaningful, we can instead evaluate the usefulness of a SNP or SNP pair directly. Namely, we would like to find a pair of SNPs which segregate a significant subset of Cases with no or very few Controls, or conversely a significant fraction of Controls with few Cases.

Odds Ratios (ORs) are commonly used to measure effect size [28] and have the advantage that they can also show whether the effect is protective or contributory. It is well known that the OR can be meaningless if the "odds" are close to zero. For contributory (deleterious) alleles this occurs when the critical parameter sensitivity ≈ 0 while for protective alleles, this is reversed and the odds ratio becomes uninformative when specificity ≈ 0. As we are only interested in either sensitivity or specificity depending on direction of association, we use the term "critical sens/spec" to refer to sensitivity and specificity depending on whether a given pair is contributory or protective. By examining the OR and the critical sens/spec we are able to summarise information on effect size, association direction and the proportion of correctly classified samples.

**Figure 2:**
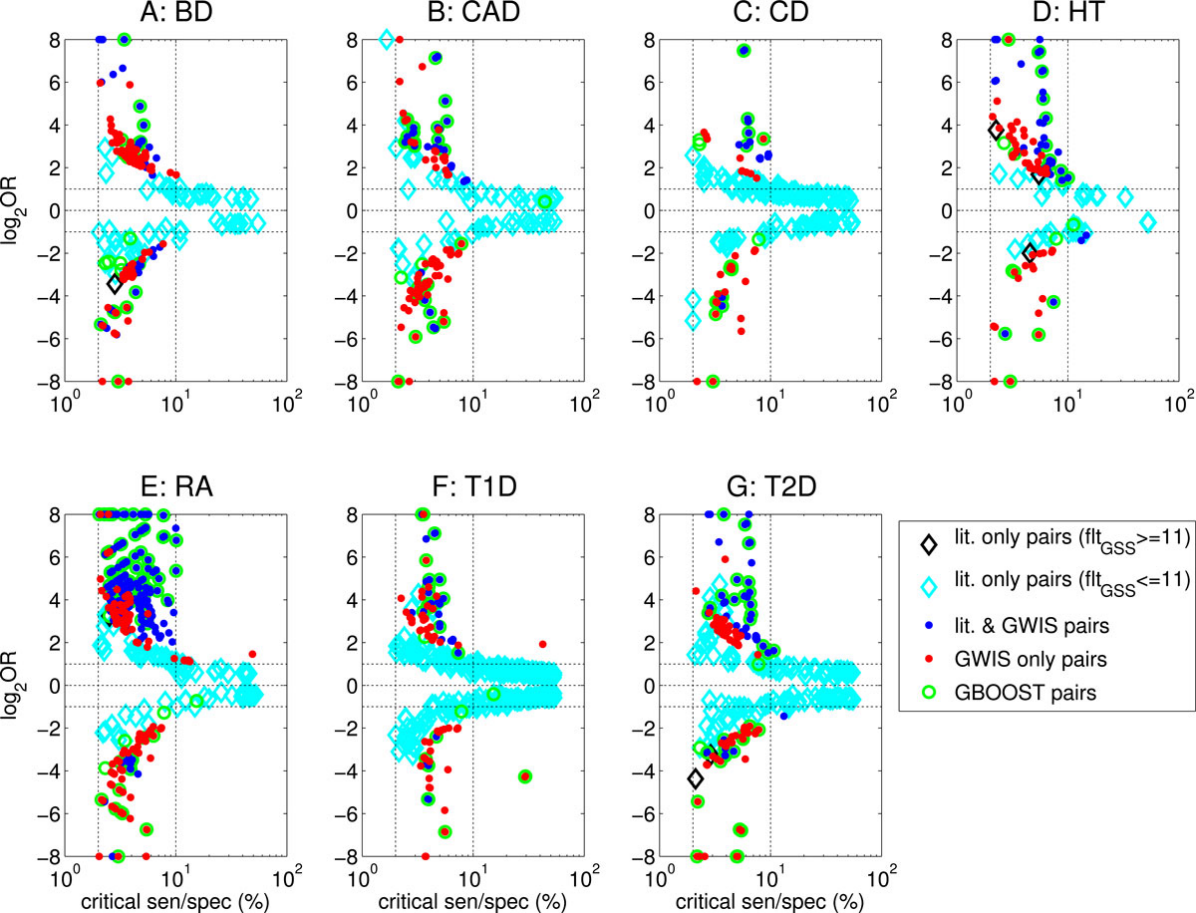
**Odds ratio (OR) vs. "critical sens/spec" of detected pairs in seven WTCCC datasets in our study and reported in literature to date**. Odds ratio (OR) vs. "critical sens/spec", i.e. sensitivity for contributing genotypes (log_2 _OR*>*0) or vs. specificity for protective genotypes (log_2 _OR*<*0). We show pairs from seven WTCCC datasets reported by GWIS or in previous literature. Results from GBOOST, an implementation of log-linear regression method, have been indicated by circles. Here we show all pairs from the full set of previous literature results that we have compiled. Each pair is represented by a point whose style indicates the methods it was reported by. There are nine pairs in the literature which pass the formal Bonferroni threshold for the gain test, $\text{fl}{\text{t}}_{\text{GSS}}>{\text{log}}_{10}\left(\begin{array}{c} 459,012\\ 2 \end{array}\right)\approx 11$ but were not detected by GWIS (black diamonds); the literature pairs which did not pass this formal requirement are marked by cyan diamonds. There are few pairs that were only detected by GBOOST (empty green circles). There is a substantial number of pairs with high odds ratios and coverage which were detected only by GWIS (red dots with no surrounding green circle) while many more were detected both in the literature and by GWIS (blue dots). The left most vertical dotted line marks the formal minimum requirement of critical sens/spec ≥ 2%, while such horizontal lines are for *log*_2_OR = ±1 corresponding to OR = 2 or OR = 1/2, respectively.

In Figure 2, we plot log_2 _OR vs. critical sens/spec for each of the SNP pairs reported as significantly interacting by GWIS, reported in previous studies or reported by both. SNP pairs identified by GBOOST when run using default parameters have been separately marked.

Although a substantial number of pairs were detected by both GWIS and literature methods, there are some discrepancies. Many pairs detected by GWIS alone often have greater odds ratios and critical sens/spec than pairs detected by the literature only. This suggests that GWIS can detect many potentially interacting pairs that are missed by methods in the literature.

All diseases show some literature pairs that have not been detected by GWIS (black and cyan diamond markers in Figure 2). These can be split into two categories. The first category, marked by black diamonds, consists of pairs which are significant according to our GSS filter but have not been reported due to shortcomings in our DSS filtering stage. They account for the 9 discrepancies between columns "Lit. after GSS' and 'Overlap" in Table 4 and are discussed later in this section.

**Table 4:** Overlap between SNP pairs found by GWIS using the DSS filter and previous studies after GSS filtering.

Disease	Lit. total	Lit. after GSS	Overlap	GWIS only	GWIS total
BD	134	57 (43%)	56	123 (69%)	179
CAD	117	37 (32%)	37	79 (68%)	116
CD	234	21 (9%)	21	36 (63%)	57
HT	93	53 (57%)	49	58 (54%)	107
RA	293	191 (65%)	190	122 (39%)	312
T1D	801	35 (4%)	35	72 (67%)	107
T2D	230	59 (26%)	56	78 (58%)	134

Overlap between SNP pairs found by GWIS and previous studies. "Lit. total" refers to the number of SNP pairs previously reported. "Lit. after GSS" refers to the number of pairs that remain significant after applying the GSS filter. "Overlap" is the set of pairs detected by GWIS and reported in literature. GWIS only and GWIS total refer to the number of pairs only detected by GWIS and the total number of pairs detected by GWIS respectively. Significance for DSS and GSS is defined by Bonferroni correction $\left(\text{p - value}={\left(\begin{array}{c} 459,012\\ 2 \end{array}\right)}^{-1}\approx 10^{-11}\right)$.

The second category, marked by cyan diamonds, predominantly consist of literature pairs where the level of improvement of the pair over its individual SNPs is insufficient to be deemed epistatic according to our stringent requirement of improvement above Bonferroni threshold, i.e. $\text{fl}{\text{t}}_{\text{GSS}}\geq \text{lo}{\text{g}}_{10}\left(\begin{array}{c} 459,012\\ 2 \end{array}\right)\approx 11$. In essence, these pair are deemed to be driven by main effects alone. This category also includes a few literature pairs that had insufficient critical sens/spec to be considered by GWIS (*<*2%, see "Minimal sensitivity and specificity" in Methods section). The supplementary Figures 22-24 show how this category changes, and the overlap with literature improves, once the Bonferroni threshold requirement is relaxed.

Recall that GWIS is intended to detect potential epistatic interactions. It is very encouraging that although GWIS' epistasis definition does not explicitly maximize odds-ratios or critical sens/spec, literature pairs with high odds-ratios and critical sens/spec are reliably detected by GWIS.

The analysis across datasets shows the expected trend of lower critical sens/spec having increased |log_2 _OR| and SNP pairs with low critical sens/spec (≤ 7%) often having very large |log_2 _OR|. These pairs are often closely located (*≤ *1Mb) and a number have been detected by previous studies. Some exceptions do exist to these trends with T1D showing a SNP pair detected only by GWIS that has OR above 4 and critical sens/spec above 30%.

As discussed earlier, GBOOST results are largely significant according to the GSS filter. There were a few points detected by GBOOST but not by GSS filtering. These pairs tended to have relatively small OR and were only just under the strict Bonferroni threshold being used.

We can also use the previous literature to provide evidence that the DSS statistic is acting as a reasonable proxy for the GSS filter. If pairs from previous literature that are significant according to GSS but were not detected by DSS, then the DSS filter has failed to detected some relevant pairs. In Table 4, we show the number of interactions reported by GWIS using the combination of DSS and GSS filters, the number of pairs reported only by previous literature after GSS filter and the number of pairs that appear in both sets of results.

The results indicate that the number of previously reported pairs that remain significant under GSS varies dramatically ranging from *≈ *4% to *≈ *66%. This large variance is likely related to the fact that different methods chose to focus on one or two WTCCC datasets rather than all seven. Datasets that show a large reduction in the number of reported pairs after GSS filtering have tended to be driven by methods searching for strong phenotype association rather than purely epistatic effects.

The "Overlap" column shows that aside from nine pairs in four diseases, all previously reported pairs that are significant under GSS were also detected by GWIS using the combination of DSS and filters. This provides additional evidence that the DSS primary filter is sensitive enough to detect pairs that are likely to be significant under GSS.

We also note that many 'novel' SNP pairs were also detected by GWIS. While re-iterating that further quality control and inspection would need to be performed to validate such pairs, it is indicative that exhaustive search combined with the statistics we propose here is likely able to detect a greater quantity of novel epistatic interactions. Such further analysis may also involve re-adjustment of the cutoff threshold to values below Bonferroni threshold used in Figure 2.

### Further validation over simulated data

To further validate our proposed statistic GSS and heuristic DSS, we evaluate their power and false positive rates over a set of synthetic benchmark datasets. The datasets chosen were generated for [23] and simulate 5 models of SNP interaction. The data shows association with phenotype only when the "true" SNPs are considered as a pair, with no association univariately. For each combination of heritability, minor allele frequency and sample-size, 500 datasets were generated, creating a total of 70 penetrance functions and 42,000 datasets. These datasets have been used to evaluate the results of several previous methods [10,23,38].

**Figure 3:**
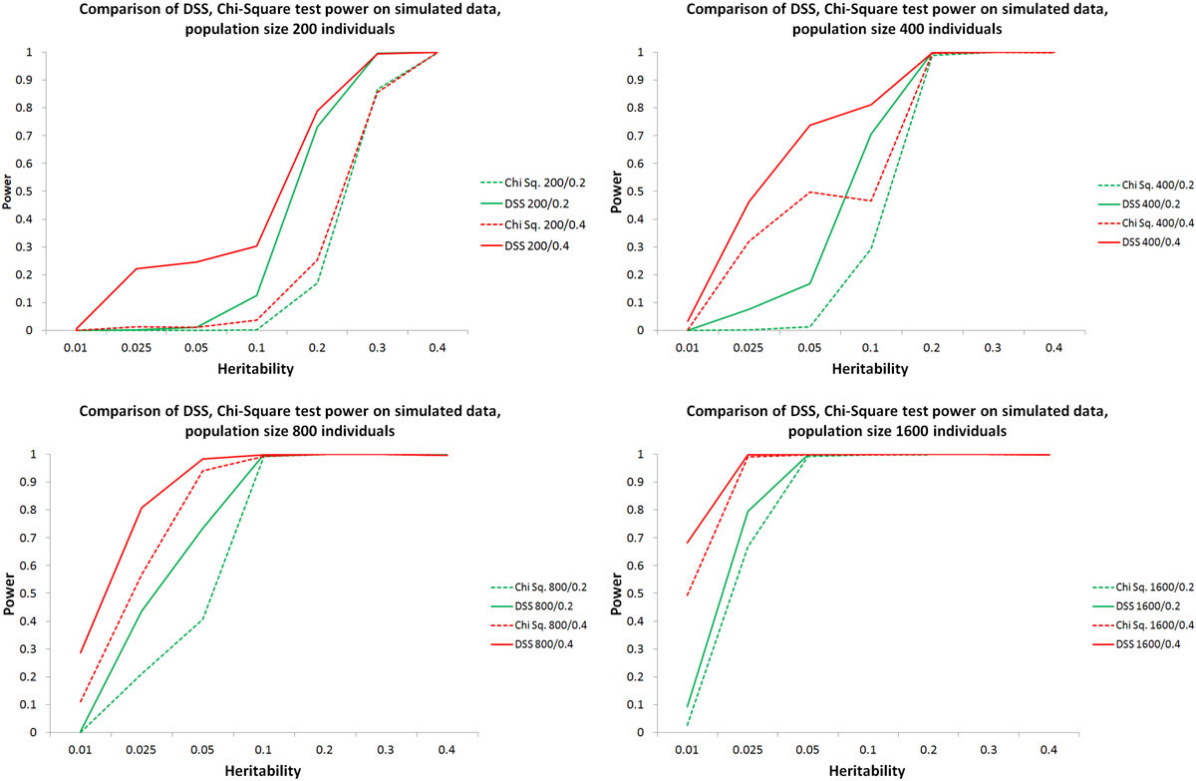
**Power of $\chi^{2}$ and the proposed DSS heuristic over simulated data**. These charts compare the power of $\chi^{2}$ and the proposed DSS heuristic to detect an epistatic pair. All DSS results are shown as solid lines; $\chi^{2}$ results are shown as dashed lines. Lines of same colour represent results from different statistics on the same simulated data. The effects of varying heritability, sample size (200, 400, 800, 1600) and minor allele frequency (0.2, 0.4) are shown here. Each data point shows the mean power over 500 randomly generated datasets. Across all parameter configurations DSS demonstrated higher power to detect the interacting pair of SNPs than $\chi^{2}$. False-positive rates for both tests (not shown here) were very low and grew linearly with the number of samples (individuals).

For each parameter combination, a single "epistatic" interaction has been embedded into each of the datasets. This allows us to calculate power (i.e. the fraction of times our method detects the "true" pair) and false positive rate (the number of other pairs falsely detected as interacting). These results are shown in Figure 3. To "detect" a pair, the computed significance has to pass a standard Bonferroni-corrected level $\left(\text{p - value}={\left(\begin{array}{c} 1000\\ 2 \end{array}\right)}^{-1}\approx 2\times 10^{-6}\right)$.We only provide results comparing our DSS heuristic and the $\chi^{2}$ statistic as it was not practical to execute GSS on the thousands of simulated datasets.

Over all the parameter combinations, DSS provided higher power than $\chi^{2}$, albeit with a slightly higher false-positive rate. This matches our expectations for DSS as a heuristic fast filter for epistasis (i.e. a manageable number of false-positives are expected). The number of false-positives from $\chi^{2}$ was extremely low (0 or 1 per 500,000 SNP-pairs) suggesting that the Bonferroni-corrected significance threshold was too strict for the $\chi^{2}$ test on this data. With a different threshold, $\chi^{2}$ might have recovered some false negative errors.

The number of false-positives from DSS was also very low, and appeared to grow linearly with increases in the number of samples. The maximum false-positive rate observed for DSS on any dataset was 0.003 and the average false positive rate over all parameter combinations was 0.001.

Although with the Bonferroni-corrected p-value threshold DSS performed better than $\chi^{2}$, these results should be viewed with caution. Both methods could have performed better with a different significance threshold. Many of the DSS false-positives could have been filtered with a stricter threshold and likewise, many of the $\chi^{2}$ false-negatives could have been detected with a weaker threshold. However, generation of p-values is intrinsic to the tests being evaluated, and in real datasets the set of true interactions is unknown making it impossible to tune the significance threshold. Our results on the WTCCC datasets show that SNP-pair p-value assignment by the DSS heuristic is of practical use for quickly finding SNP-pairs with characteristics suggestive of phenotype association. Although we could have adjusted the p-value threshold to suit either algorithm, we felt the strict Bonferroni level is the only meaningful threshold that could be applied to real world data and therefore the only threshold that is justifiable on simulated data.

While these figures validate our proposed DSS filter, it is worth noting that the simple scenario of a single epistatic interaction is unlikely to emulate that of real datasets, and as such, the conclusions that can be drawn from current synthetic benchmarks, including that used here, are limited. For instance, the QQ plots in Additional File Figure 2 clearly indicate that in the real-life WTCCC data used in previous sections the DSS filters yield systematically fewer false positives than $\chi^{2}$ filters, contrary to the observations for simulated data above. We elaborate on this in the Discussion section.

## Discussion

### Improved efficiency allows analysis on current and future datasets

In recent years, there have been several proposals that exploit the inherently parallelisable structure of GWAS data to provide reasonably fast solutions capable of processing a WTCCC dataset in several hours. However, SNP arrays currently being used in GWAS studies are an order of magnitude larger [39], resulting in two orders of magnitude increase in the number of pairs and a pressing need for ever more efficient processing of GWAS. Moreover, datasets are often processed repeatedly as data and parameters are altered, quality control measures applied or to correct for population and batch effects, meaning that effective research demands rapid processing. The analysis of higher-order interactions will also dramatically increase the computational burden of epistasis detection. Combined, these points indicate that multivariate GWAS analysis is still a computational challenge.

Our method provides faster discovery of epistatic interactions, which enables more effective, interactive usage. The tool provides an efficient and fast screening capabilities that can be run locally on researchers' desktop computers rather than expensive computing clusters. The reported results can then be refined with more computationally expensive methods such as logistic regression or permutation testing, or in combination with additional biological reference material.

### Feasibility of exhaustive search removes the need for ad-hoc constraints

As indicated by several previous publications [21,24,40], there is a need for exhaustive search over all bivariate associations in Case-Control studies. While there are several established heuristics that aim to reduce the number of pairs considered, they all have corresponding weaknesses.

**Figure 4:**
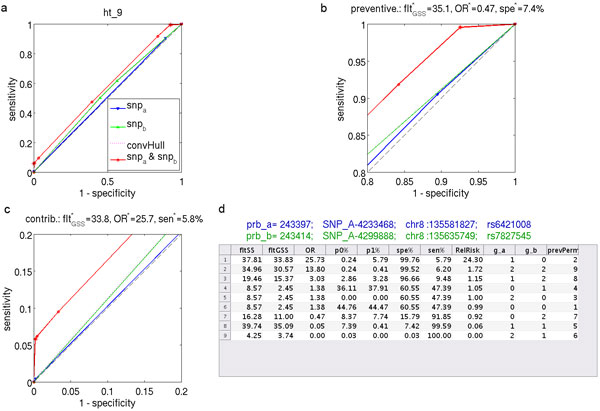
**Example of a pair of individually insignificant SNPs in HT data**. Example of a pair of individually insignificant SNPs in HT data that combined display both strong protective and contributing effects. Panel (a) shows prevalence mapping ROC curves for the pair (red) and individual SNPs (blue, green). Panels (b) and (c) zoom into the protective (top-right 20%) and contributory (lower-left 20%) areas respectively. Panel (d) shows selected statistics for the pair. The nine rows correspond to possible genotype calls for the pair of SNPs. Columns are: flt_SS _- the sensitivity-specificity filter score; flt_GSS _- the gain filter score; OR - Odds-Ratio; *p*0 - percentage of Controls for the genotype call in this row; *p*1 - percentage of Cases; spe% - specificity %; sen% - sensitivity %; RelRisk - relative risk for the genotype call := *p*1/*p*0; *g*_1_, *g*_2 _- genotype calls for the pair of SNPs; prevPerm - prevalence permutation (see Additional File Section 1.2). The genotype call (1, 0) (row 1) segregates 5.79% of Cases with only 0.24% of Controls resulting in an odds ratio of 25.73, flt_SS _= 37.81 and flt_GSS _= 33.83. Conversely, genotype calls {(1, 1), (2, 1)} (rows 8 and 9) cover 7.39% of Controls with only 0.41% of Cases resulting in an odds ratio of 0.05. This combination is also highly significant with flt_SS _= 39.74 and flt_GSS _= 35.9. The two points corresponding to these calls are highlighted with stars.

A popular strategy is to consider only pairs containing univariately strong SNPs [38,40] or pairs that have been ranked highly by feature selection techniques [14,41]. The obvious drawback with this approach is that some SNPs with strong epistatic association in pairs may show little association with phenotype individually, and therefore this constraint is likely to remove many of the pairs we want to identify (see examples in Figure 4 and Additional File Figures 7-10 and 13).

An alternative strategy is the use of known biological data. Here, the number of SNPs examined is reduced to those with prior evidence of possible epistatic effects [42] or that can be mapped to known biological networks [35]. These strategies are likely to be hindered by a lack of epistasis understanding in complex organisms.

Distance constraints, in which SNP pairs are discarded if they are too close together [10,24,32], are commonly used with some evidence [26,43] indicating that such pairs may be linked to genotyping errors. However, it is not always clear that all closely located SNP pairs are due to genotyping errors [26]. Moreover, some recent methods [44] have been designed specifically to look for pairs that were closely located, in order to find associations caused by non-typed SNPs.

The feasibility of exhaustive search as demonstrated in this work removes the need for such constraints. Exhaustive search can examine all possible SNP pairings and, if a robust statistical filter is used, will greatly reduce the set of epistatic interactions requiring follow-up analysis. Further filtering can then be applied to remove those SNP pairs that are not relevant for a given experiment.

### Comments on the definition of epistasis

Our prime goal in this paper is to present a practical system capable of exhaustive search through all SNP pairs in real, full scale GWAS, detecting all pairs evidencing significant epistatic effects. This requires a robust definition of epistasis which can be translated into an actionable mathematical algorithm [11].

Operationally, epistatic interaction means in this paper two things:

(i) that there exists a scoring function of genotype calls for the pair of SNPs and a decision threshold such that a substantial subset of subjects scoring above the threshold is significantly enriched (biased) in either Cases or Controls, and the split of the sample according to this threshold results in OR significantly different from 1;

(ii) for any scoring function depending on a single SNP of the pair only, such an enrichment is highly unlikely to be achievable by re-sampling data from the population.

In particular, our definition captures three examples of penetrance tables for "non-standard" epistatically interacting loci discussed by Cordell [11, Tables 1, 2, 3], and moreover, this can be done with a suitable choice of "purely additive" scoring functions and appropriate decision thresholds (no need for any cross-terms). In that respect our generic formal definition of epistasis is closer to its biological counterpart than Fisher's definition of interaction [12], which focuses on fitted models' deviation from additivity. Note that even the original review of Fisher's paper pointed out that his definition does not capture a number of biologically plausible aspects of epistatic interaction, see [11]. However, Fisher's definition is mathematically sound and thus widely used in analysis of contingency tables in statistical literature [28], in quantitative genetics [21] and has been applied in a number of GWAS analysis papers using model based regression approaches [10,26,35,37,45-47].

### Analysis of real datasets may improve simulated data

Despite advances in speed, the most common benchmark for epistasis remains simulated data, where a single epistatic interaction embedded in a small number of SNPs is used to judge a method's power and false positive rate under various parameter settings. In this work, we evaluate the power of a standard $\chi^{2}$ and our proposed DSS filter over many such datasets. In conjunction, we also extensively and exhaustively examined multiple real life GWAS, revealing complexities such as confounding signals generated by highly associated univariate SNPs and multiple epistatic signals of varying strength. Such complexities are rarely modelled together in a single epistasis simulation and indicates limitations in the ability of simulated data to be indicative of true power or false positive rates. We believe that further analysis of real data may help better characterise the complexities of GWAS which can be used to create more realistic simulated data. Broader scenarios with multiple epistatic, non-epistatic and univariate signals may better emulate the complexities which we believe are still hidden in real datasets.

### Univariate associations can have a confounding effect on standard tests for association

Using the $\chi^{2}$ statistic as a filter to detect epistatic SNP pairs, we discovered that top-ranked SNP pairs were almost always driven by univariately strong SNPs. If a dataset contains a SNP with strong univariate association its pairings with random SNPs will cause the $\chi^{2}$ filter to report many thousands of SNP pairs that show an association with phenotype but do not show epistatic-like effects according to our definition.

Studying pairwise associations in GWAS data is necessarily a filtering process, reducing the billions of possible interactions by several (5 or 6) orders of magnitude down to a small number that can be analysed in detail. In order to have any chance of discovering epistatic interactions, the majority of pairs of SNPs that show little improvement over their univariate associations must be explicitly discarded; in other words, we must specifically look for pairs of SNPs that together show *improved *association with phenotype.

Empirical evidence showing the impact this confounding has on the $\chi^{2}$ statistic provided in this paper is intrinsically interesting. Indeed, $\chi^{2}$ filtering has been used in bivariate analysis of GWAS in the past using the standard $\chi^{2}$ test directly [25,29,30] or some variant of it [27,32,48,49]. It is also likely that the same confounding will affect other standard tests for association. Such confounding has been previously observed but has rarely been dealt with in a rigorous manner that is not based on regression. Our GSS/DSS test, explicitly searching for gains in specificity and sensitivity, is a new, efficient alternative in this regard.

### Multivariate analysis increases the need for stringent quality control and follow-up analysis

GWIS is a model-free method for detecting epistatic SNPs, designed to be sensitive to any associations in the data that separate Cases and Controls. However, this separation may be due to signals other than that caused by phenotype. It has been noted that pairwise SNP analysis may be more susceptible to noise caused by genotyping errors, population structure or batch effects [26,43] compared to univariate analysis and reported interactions may be a product of these sources of noise. Given that these will vary between experiments follow-up analysis of reported interactions, especially quality control of genotype calls, remains critical for determining their validity.

## Methods

In this section we outline various filtering procedures used in this paper for detection of putative epistasis loci. We shall focus particularly on the receiver operating characteristic (ROC) analysis method, which is part of the novelty of this paper. More details and formal descriptions have been shifted to the Additional File 2 Materials and forthcoming papers will contain the full details and formal proofs.

### ROC analysis for GWAS

Here we outline three particular "model free" statistical filtering methods implemented in GWIS and explicitly used in this presentation.

Our filtering approach quantifies the ability of a pair of SNP-probes to segregate Cases from Controls in available data sample compared to the segregation ability of the two SNP-probes taken individually. There are a number of methods in the literature that attempt to measure this type of improvement for epistasis detection, e.g. BOOST uses the decrease in residual error between additive and full interaction regression models [10] while the “random chemistry” approach of Eppstein et. al. [50] uses Euclidean distance between ROC curves.

The key distinct features of our method can be summarised follows:

*• *It is based on ROC curve analysis, focussing on classification rather than regression;

*• *The filters use an exact quantification of underlying probability distributions rather than relying on asymptotic normality;

*• *The approach permits a natural interpretation that links the properties of the sample data back to population data.

With each SNP-probe or pair of SNP-probes, we associate a *sample prevalence mapping*, allocating to each individual the ratio of the number of Cases to the sum of Cases and Controls in the dataset which carry exactly the same genotype as this individual. For any pair of SNP-probes we have three such prevalence mappings, one for the pair and two for the individual probes. Each mapping can be used to construct a ROC curve: the plot of the *true positive rate *(TPR) versus the *false positive rate *(FPR). These are piecewise linear curves. Specifically, a 9-piece ROC curve for the pair, *ROC *(*g*_1_, *g*_2_), which dominates both 3-piece curves *ROC*(*g_i_*), *i *= 1, 2, for the individual SNP-probes. This domination results from the increased number of genotype calls for a pair of SNPs which allows for finer stratification of the data. For most probe pairs, this stratification will have little effect on the ability to separate Cases from Controls but for some the difference will be significant.

**Figure 5:**
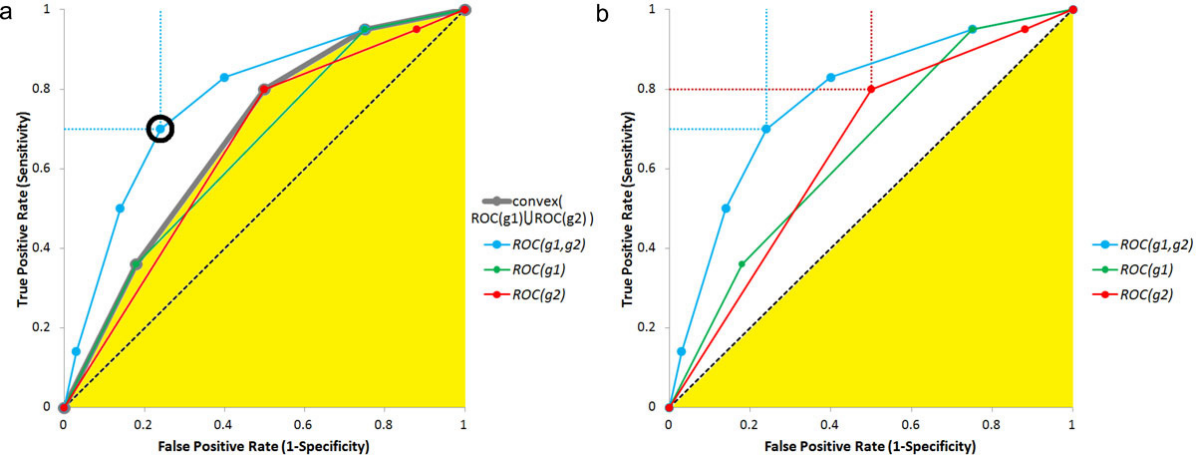
**Illustration of the principles underlying the GSS and DSS filters**. Illustration of the principles underlying the GSS (a) and DSS (b) filters.

For any specific sensitivity and specificity value, say (*se*, *sp*), achieved by the pair of SNP-probes, we have to determine the probability of observing equal or higher specificity and sensitivity due to biased sampling from the population for which true specificity and sensitivity falls in the region below either of the ROC curves for the individual SNPs. When the ROC curve for the pair overlaps any of the individual SNP curves, this probability will be close to 1, hence not significant. However, as a measure of potentially improved capability of the pair, it is natural to use the most significant improvement, i.e. the smallest such p-value, corresponding to the circled dot in Figure 5. Here, in order to reduce computations we use a slightly expanded region which is the convex region encompassing both ROC(*g_i_*) curves for individual probes. This expansion is conservative in the sense that it produces less significant, i.e. increased p-values. We shall refer to this smallest probability value as *P*_GSS_, the *p-value for gain in sensitivity and specificity*, and introduce the following notation for their negative log_10_:

(1) $$\text{fl}{\text{t}}_{\text{GSS}}\left(g_{1},g_{2}\right)\;:=-\;\text{lo}{\text{g}}_{10}\;P_{\text{GSS}}\left(g_{1},g_{2}\right).$$

This will be referred to as the score or output of the GSS*-filter*.

The crux for our approach is to compute *P*_GSS _by solving the following min-max optimization

(2) $$P_{\text{GSS}}\left(g_{1},g_{2}\right):\;=\underset{\left(x_{0},x_{1}\right)}{\text{min}}\underset{(\pi_{0},\pi_{1})\in H_{0}}{\text{max}}\overset{x_{0}}{\underset{i=0}{\sum }}\left(\begin{array}{c} t_{0}\\ i \end{array}\right)\;\pi_{0}^{i}{\left(1-\pi_{0}\right)}^{t_{0}-\;i}\overset{t_{1}}{\underset{j=x_{i}}{\sum }}\left(\begin{array}{c} t_{1}\\ j \end{array}\right)\;\pi_{1}^{j}{\left(1-\pi_{1}\right)}^{t_{1}-\;i},$$

where "min" is over all cumulative counts *x*_0 _and *x*_1 _of Cases and Controls such that

(3) $$\left(\frac{x_{0}}{t_{0}},\frac{x_{1}}{t_{1}}\right)\in \;\text{ROC}\left(g_{1},g_{2}\right)$$

while the "max" is over the smallest convex region $H_{0}$ of the unit square *I*^2 ^:= [0, 1]^2 ^containing ROC(*g*_1_) and ROC(*g*_2_), see the shaded region in Figure 5; and *t*_0_, *t*_1 _are the total numbers of Controls and Cases in the sample dataset, respectively. In this case π_0 _and π_1 _represent the (unknown) population proportion of deleterious alleles in Controls and Cases respectively. For a given point on the ROC curve (defined by *x*_0 _and *x*_1_), maximizing over the unknown population probabilities corresponds to a worst case scenario for rejection of the null hypothesis $\left(\pi_{0},\pi_{1}\right)\in H_{0}$, with the *p*-values quantifying the largest probability of observing a sensitivity greater than *x*_1_/*t*_1 _and a specificity greater than 1 - *x*_0_/*t*_0 _by biased sampling. The true p-value, for the actual (π_0_, π_1_) for the population, must obviously be less than this. Minimizing over the pairs of points of ROC(*g*_1_, *g*_2_) curve gives the set of alleles with the "best" capability to discriminate Cases from Controls.

The optimisation itself is relatively easily computable on modern hardware with carefully crafted algorithms. More details are given in the Supplement and [51].

The above optimisation *P*_GSS _has to be solved separately for each pair of probes which will create a pair-specific null hypothesis *H*_0_. It is convenient and meaningful to consider the special case of (2) for $H_{0}:=\left\{\pi_{1}\leq \pi_{0}\right\}$ which is the part of *I*^2 ^below the main diagonal. It can be shown that in such a case the whole optimisation (2) reduces to optimisation against the diagonal $H_{0}=\left\{\pi_{0}=\pi_{1}\right\}$ itself. This corresponds to the classical hypothesis test for a simple null hypothesis that probes have no segregation power and the observed separation is purely due to biased sampling. This form of the hypothesis test is close to the classical small-sample unconditional test of independence [28]. The resulting probability will be referred to as the *p-value for sensitivity and specificity test*, and can be computed as

(4) $$P_{\text{SS}}\left(g_{1},g_{2}\right):=\underset{(\frac{x_{0}}{t_{0}},\frac{x_{1}}{t_{1}})\in \text{ROC}{(g}_{\text{1}}\text{,}g_{\text{2})}}{\text{min}}\underset{0\;\leq \;\pi_{0}\leq 1}{\text{max}}\overset{x_{0}}{\underset{i=0}{\sum }}\left(\begin{array}{c} t_{0}\\ i \end{array}\right)\;{\pi_{0}}^{i}{\left(1-\pi_{0}\right)}^{t_{0}-i}\overset{t_{1}}{\underset{j=x_{1}}{\sum }}\left(\begin{array}{c} t_{1}\\ j \end{array}\right)\;{\pi_{0}}^{j}{\left(1-\pi_{0}\right)}^{t_{1}-j}.$$

In this case π_0 _and π_1 _again represent the (unknown) population proportions of deleterious alleles in Controls and Cases, respectively, but since the null hypothesis is in fact restricted to the main diagonal, the optimisation over the population parameters reduces to maximisation over a single variable π_0_. The interpretation is as before with the "max" part corresponding to an upper limit on the true p-value and minimisation over the pairs of points corresponding to selection of the smallest such upper limit, thereby giving the most significant improvement of the pair is a classification of individuals into Controls and Cases using the pair's genotype calls over bias sampling from hypothetically inseparable population.

The crucial, "max" part of this statistic can be easily tabulated (as a function of counts (*x*_1_, *x*_2_)), and therefore *P*_SS _is relatively easy to implement in practice for exhaustive scanning of probe-pairs as a primary filter.

The definition of *P*_SS _above is naturally extendable to the case of single genotyping probe: namely, *P*_SS_(*g_i_*) is defined by (4) if we replace ROC(*g*_1_, *g*_2_) by ROC(*g_i_*). This brings us to the introduction of the following proxy for flt_GSS _filter (c.f. Figure 5.b):

(5) $$\text{fl}{\text{t}}_{\text{DSS}}\left(g_{1},g_{2}\right):\;=-{\text{log}}_{10}\frac{P_{\text{SS}}\left(g_{1},g_{2}\right)}{\text{min}\;\left(P_{\text{SS}}\left(g_{1}\right),P_{\text{SS}}\left(g_{2}\right)\right)}=\text{fl}{\text{t}}_{\text{SS}}\left(g_{1},g_{2}\right)-\text{max}\;\left(\text{fl}{\text{t}}_{\text{SS}}\left(g_{1}\right),\text{fl}{\text{t}}_{\text{SS}}\left(g_{2}\right)\right),$$

where

$$\text{fl}{\text{t}}_{\text{SS}}\left(g_{1},g_{2}\right)\;:=-{\text{log}}_{10}P_{\text{SS}}\left(g_{1},g_{2}\right).$$

We shall call flt_SS _and flt_DSS _the filters for SS and DSS, respectively. The fit_DSS _quantifies an improvement of a pair over its individual constituents allowing it to act as a computationally inexpensive proxy for flt_GSS _which is suitable for scanning massive numbers of pairs (*g*_1_, *g*_2_); see Additional File Figures 3 and 4.

### Odds ratio

GWAS studies aim in particular at identification of genomic rare variants in the population which are associated with increased or decreased risk of developing a disease. At data filtering stage, the main focus in this paper, we would like to identify SNP-pairs and sets of genotyping calls which allow us to identify subsets of the dataset with an OR for developing disease significantly different from 1. There are two possibilities illustrated by example in Figure 4. The first of them, the *contributing or high risk *scenario, (*cntr ≡ OR *≫ 1), is illustrated in subplot (c). Here the red star corresponds to set of genotype calls with the highest flt_GSS _for contributing gain, which happen to be a singleton set {(1, 0)}. The carriers of this genotype constitute *ξ*_1_:= *x*_1_/*t*_1 _= 5.79% of Cases and *ξ*_0_:= *x*_0_/*t*_0 _= 0.24% Controls, resulting in extremely high odds ratio *OR *= 25.73 and significant flt_GSS _= 33.83. The opposite, *protective *scenario, (*prtv ≡ OR *≪ 1), illustrated in Figure 4(b). Here we find that for the set two genotype calls, {(2, 1), (1, 1)}, we have very low number of Cases carrying these genotypes, *ξ*_1 _= 0.42% and relatively high fraction of Controls *ξ*_0 _= 7.39% resulting in *OR ≈ *0.05. In the contributing scenario we would like to increase *ξ*_1 _= SEN and decrease *ξ*_0 _to *≈ *0; in the protective situation, we would like to maximize *ξ*_0 _= SPE with simultaneous reduction of *ξ*_1 _to ≈ 0.

### Implementation of tests

The above section introduces principles on which our custom filtering algorithms are built. In this subsection we describe some additional enhancements and heuristics which were added to practical implementations used.

#### Protective and contributing capabilities

As we have discussed above, for any *k*-tuple of genotyping features we may find subsets of their values displaying different degree of protection or contribution to the phenotype in question. One obvious modification to the above GSS test is to extend it to two separate tests, one for protective the other for contributing capabilities. The heuristic which we have followed in this regard consisted in restricting the "max" in computing *P*_GSS _once to a subset ROC*_cntr_*(*g*_1_, *g*_2_) contributing alleles, and another to a subset ROC*_prtv_*(*g*_1_, *g*_2_) of protective alleles. The demarcation is defined as follows. Let $\left(\frac{x_{0}\left(i\right)}{t_{0}},\frac{x_{1}\left(i\right)}{t_{1}}\right)$, *i *= 0, 1, ..., 9 denote the (ordered) sequence of 10 points of ROC(*g*_1_, *g*_2_). Then

$$\begin{array}{llll} \text{RO}{\text{C}}_{cntr}\left(g_{1},g_{2}\right)\; & :=\left\{\left(\frac{x_{0}\left(i\right)}{t_{0}},\frac{x_{1}\left(i\right)}{t_{1}}\right)\;\;\left|\;\frac{x_{1}\left(i\right)-x_{1}\left(i-1\right)}{t_{1}}\right)/\frac{x_{0}\left(i\right)-x_{0}\left(i-1\right)}{t_{0}}\geq 1,\;\;i=1,...,8\right\},\; & & \;\\ \text{RO}{\text{C}}_{prtv}\left(g_{1},g_{2}\right)\; & :=\left\{\left(\frac{x_{0}\left(i\right)}{t_{0}},\frac{x_{1}\left(i\right)}{t_{1}}\right)\;\left|\;\frac{x_{1}\left(i+1\right)-x_{1}\left(i\right)}{t_{1}}\right)/\frac{x_{0}\left(i+1\right)-x_{0}\left(i\right)}{t_{0}}\leq 1,\;\;i=1,...,8\right\}.\; & & \;\\ & \; & \end{array}$$

#### Minimal sensitivity and specificity

Both SS and GSS tests are capable of identification of genotype probes which allow for strong separation in relatively small fractions of the population. This is a desired property for detection of rare variants. However, in practice the limited sample size imposes practical limitations on minimal size which could be of practical interest and is immune to noise or numerical instability of the optimisation procedures used. In our analysis we demanded that in computation of the outer minimum in either (2) or (4) we disregarded all contributions from *x*_0_, *x*_1 _such that min(1 *- x*_0_/*t*_0_, *x*_1_/*t*_1_) *<*0.02.

#### Limited precision implementation

The solution of this optimisation is not straightforward, for an average GWAS, *t*_0 _and *t*_1 _have sizes measured in thousands. This means in practice that in evaluating (2) and (4) we need to deal with multiplications, divisions and summations of thousands of numbers either so small or so large that they cannot be represented directly in computer hardware. For the description of the specific procedures developed to deal with this task and presentation of related formal proofs of their correctness we refer to a dedicated methods paper [51]. Here we only outline the main steps of those derivations:

• First, we prove that the functions under "max" in (2) and (4) have no local maxima;

• For (2) the maximum is achieved on the boundary of $H_{0}$.

• Due to that uniqueness, we can efficiently use any iterative procedure for finding the maximum. In particular we have used the bisection method, which converges to the solution along the boundary.

• Finally, for numerical efficiency we have developed specific numerical simplifications which effectively reduce computation of the sums in (2) and (4) down to additions of small numbers of terms of order of one, with provably negligible penalty errors.

With the simplifications outlined above, the computation of values for individual probes and probe-pairs becomes a tractable numerical task. However when it comes to an exhaustive tabulation of the whole 2-dimensional distribution underpinning computation of *P_SS _*for tens of thousands of possible values of counts *x*_0 _and *x*_1_, hence for the multiple millions of pairs (*x*_0_, *x*_1_), the computing burden could become significant, warranting additional simplifications and reductions. In the case of GSS the computational burden is even harder, direct scan with this statistical filter becomes impractical (see Additional File Section 4), and so arises the need for developing more efficient proxies such as DSS (5).

### Other filters used

We have used a number of other techniques than those described above for filtering putative interactions in GWAS. We outline them here for completeness.

#### $\chi^{2}$ for independence

This is one of the most popular methods for interactions detection in GWAS. It has two distinct components:

• *Computation of *$\chi^{2}$*statistics*. This is a well defined statistic which could be used directly for ranking of hits;

• *Computation of p-value for determination of significance*. This part is is more complex and the usual solution is to apply a formula which is rigorously derived for sampling from a normal distribution [28].

We have used such formulae with 8 and 2 degrees of freedom when dealing with bivariate or univariate analysis, respectively. Additionally, we have applied the $\chi^{2}$ distribution with 4 degrees of freedom to scores derived by the BOOST algorithm, following the original recommendation of the authors of that method (see [7]). In all those cases we have serious reservations regarding allocation of such p-values (see Discussion for an elaboration of this point).

We compute the following standard $\chi^{2}$ statistic for the contingency Table 5, see [28,52]:

**Table 5:** 2 × *V*-contingency table.

	Genotype Frequencies				
**Phenotype**	**1**	**2**	**...**	**V**	**Row Counts**

Controls ≡ 0	*n*_01_	*n*_02_	...	*n*_0*V*_	*t*_0_
Cases ≡ 1	*n*_11_	*n*_12_	...	*n*_1*V*_	*t*_1_

Col. Counts=	*n*_:1_	*n*_:2_	...	*n*_:*V*_	*n*

2 × *V *-contingency table.

(6) $$X^{2}={\left(\overset{1}{\underset{i=0}{\sum }}\underset{\upsilon \in V}{\sum }\frac{{\left(n_{i\upsilon }-E_{i\upsilon }\right)}^{2}}{E_{i\upsilon }}\right|}_{E_{i\upsilon }=\frac{t_{i}n_{:\upsilon }}{n}}.$$

This statistic is known to have *approximately $\chi^{2}$*distribution with *V *- 1 degrees of freedom [28,52], which is used to allocate the p-values. Note, if the null hypothesis *H*_0 _: *N_iυ _*= *E_iυ _*for all *i*, *υ *holds, then *X*^2 ^= 0.

#### Fisher Exact test

Fisher Exact test is often used for evaluation of 2×2 contingency tables [28] and as such can be applied for allocation of p-values to observed cumulated count (*x*_0_, *x*_1_). Such p-values turn out to be in fact very close numerically to the *P*_ss _test, see Additional File Figure 21. For that reason we did not scan data with Fisher Exact test based filters, but the SS filter is a good indicator of its performance.

#### BOOST

We have used BOOST and GPU version GBOOST algorithms which we have downloaded from the web, and for details we refer to [7,10]. These algorithms perform exhaustive search though all pairs of probes, but they use different methodology: they use log-linear regression rather than classification and asymptotically justified approximation for allocation of p-values to derived scores, the 4-degree of freedom $\chi^{2}$ test (see [31]).

## List of abbreviations

DoO: Difference of Odds; DSS: Difference in Sensitivity and Specificity; FE: Fishers Exact; FPR: False positive rate; GPU: Graphics Processing Unit; GWAS: Genome wide association studies; GWIS: Genome Wide Interaction Search; GSS: Gain in Sensitivity and Specificity; OR: Odds Ratio; ROC: Receiver operating characteristic; SNP: Single nucleotide polymorphism; SS: Sensitivity and Specificity; TPR: True positive rate; WTCCC: Wellcome Trust Case-Control Consortium.

WTCCC Datasets

BD: Bipoloar Disorder; CAD: Coronary Artery Disease; CD: Crohn's Disease; HT: Hypertension; RA: Rheumatoid Arthritis; T1D: Type I Diabetes; T2D: Type II Diabetes.

## Authors' contributions

BG contributed to development and initial and final implementation of algorithms used, carried out most numerical experiments, and drafted the manuscript. DR implemented and optimised CPU implementation, performed simulations and assisted in writing and revising manuscript. QW implemented and optimized GPU version of algorithms, performed benchmarking including third party software, and collated results for literature comparison. FS assisted in development of software for analysis of results, and collated results for literature comparison. HF developed software for analysis of results, contributed to analysis of data, and assisted in critical revisions of manuscript. RC has critically revised and analysed results and manuscript. LS assisted in critical revisions of manuscript. MI helped get access to data and critical revised manuscript. CSO contributed to writing of manuscript, specifically for discussion, and contributed to comparison against literature. AK conceived of the study and participated in its design, designed and developed prototypes of methods used, implemented software for analysis of results, coordinated the project and helped to draft the manuscript.

All authors read and approved the final manuscript.

## Competing interests

The authors declare that they have no competing interests.

## Supplementary Material

Additional file 1 Supplementary Materials Click here for file

Additional file 2 Lists of detected SNP pairs Click here for file
