# Supplementary material for: GWIS - model-free, fast and exhaustive search for epistatic interactions in case-control GWAS
Source: BMC Genomics. 2013 May 28;14(Suppl 3):S10. doi: 10.1186/1471-2164-14-S3-S10 (PMC3665501; doi:10.1186/1471-2164-14-S3-S10)
Supplement: Additional file 1 — Supplementary Materials [file 1471-2164-14-S3-S10-S1.pdf]

GWIS - Model-free, Fast and Exhaustive Search for Epistatic Interactions in Case-Control GWAS
Supplementary Materials

Benjamin Goudey*[1,2] , David Rawlinson[1,3], Qiao Wang[1,2], Fan Shi[1,2], Herman Ferra[1], Richard M. Campbell[1,3], Linda Stern [2], Michael Inouye [4,5], Cheng Soon Ong[1,2], Adam Kowalczyk*[1,2]

[1]National ICT Australia Victorian Research Lab, and Departments of
[2]Computing and Information Systems,
[3]Electrical and Electronic Engineering,
[4]Pathology,
[5]Microbiology & Immunology,
The University of Melbourne, Parkville, Victoria, Australia

Email: Benjamin Goudey*- bwgoudey@csse.unimelb.edu.au; Adam Kowalczyk*- adam.kowalczyk@nicta.com.au;

*Corresponding author

February 24, 2013

1 Supplementary: Methods

In this section we first outline the principle of a very general procedure which underpins the main methodology for detection of interactions in GWAS used in this paper. Then we elaborate some technical results leading to numerical algorithms for its practical implementation.

We consider a finite sample $\mathcal{S} = \mathcal{S}_0 \cup \mathcal{S}_1$ split into two subsets of Controls and Cases respectively and two functions $f_i : \mathcal{S} \to \mathbb{R}$, $i = 1, 2$. Those functions can be viewed as two predictive models designed to separate Cases from Controls. For each of them we consider the ordinary $\text{ROC}(f_i)$, $i = 1, 2$ curve [1], the plot of sensitivity vs specificity, (see Figure 1). Assume that the second $\text{ROC}(f_2)$ curve (the blue curve in Figure 1) is dominating the first one (the red curve in Figure 1). The main question for us is as follows. How to quantify the extent to which the second classification model is better than the first one? How to determine whether this difference is significant or not? The gist of our answer is compacted in the two statements below:

- The gain of f_2 over f_1 is quantified by the most significant probability (i.e. the smallest p-value) that a specificity and sensitivity achieved by f_2 at any point of $ROC(f_2)$ can be exceeded by random

sampling of Controls and Cases from a population for which the true sensitivity and specificity are in the region bounded by $ROC(f_1)$.

- This probability is essentially dependent on the sample sizes, $t_0 = |S_0|$ and $t_1 = |S_1|$

The rest of this section elaborates the above in a more precise fashion.

Once this probability is derived, its significance can be assessed in a principled manner. The computation of this p-value can be easily cast as a standard test against a null hypothesis, with the above probability becoming the formal p-value for its rejection. However, crucial here is that this probability can be computed as the solution of a min-max optimisation, even in the case of relatively large sample sizes encountered in GWAS studies.

Now we outline the main formal results leading to a solution of this computational problem. The full details will be presented elsewhere.

1.1 SS test for a ROC curve

We start with formal introduction of our model. We consider a population $\mathcal{P} = \mathcal{P}_0 \cup \mathcal{P}_1$ split into two disjoint subsets of Controls and Cases, respectively, from which we have sampled a relatively small subset $\mathcal{S}$. We assume $\mathcal{S}_i := \mathcal{P}_i \cap \mathcal{S}$ and $t_i := |\mathcal{S}_i|$ for $i = 0, 1$.

We recall, for a real function $f : \mathcal{P} \to \mathbb{R}$ and a threshold $\theta \in \mathbb{R}$, the true positive rate (TPR) or sensitivity on S is defined as

$$\text{TPR}_{\mathcal{S}}(f, \theta) = \text{SEN}_{\mathcal{S}}(f, \theta) := \frac{|\{f \geq \theta | \mathcal{S}_1\}|}{t_1};$$

the *false positive rate (*FPR*)* is defined as

$$\text{FPR}_{\mathcal{S}}(f, \theta) := \frac{|\{f \geq \theta | \mathcal{S}_0\}|}{t_0}$$

and it is related to the *specificity*

$$\text{SPE}_{\mathcal{S}}(f, \theta) := \frac{|\{f < \theta | \mathcal{S}_0\}|}{t_0}$$

by the equation

$$\text{FPR}_{\mathcal{S}}(f, \theta) = 1 - \text{SPE}_{\mathcal{S}}(f, \theta).$$

The *Receiver Operating Characteristics (*ROC*) curve* is defined here as a subset of the unit square $\mathbb{I}^2 :=$

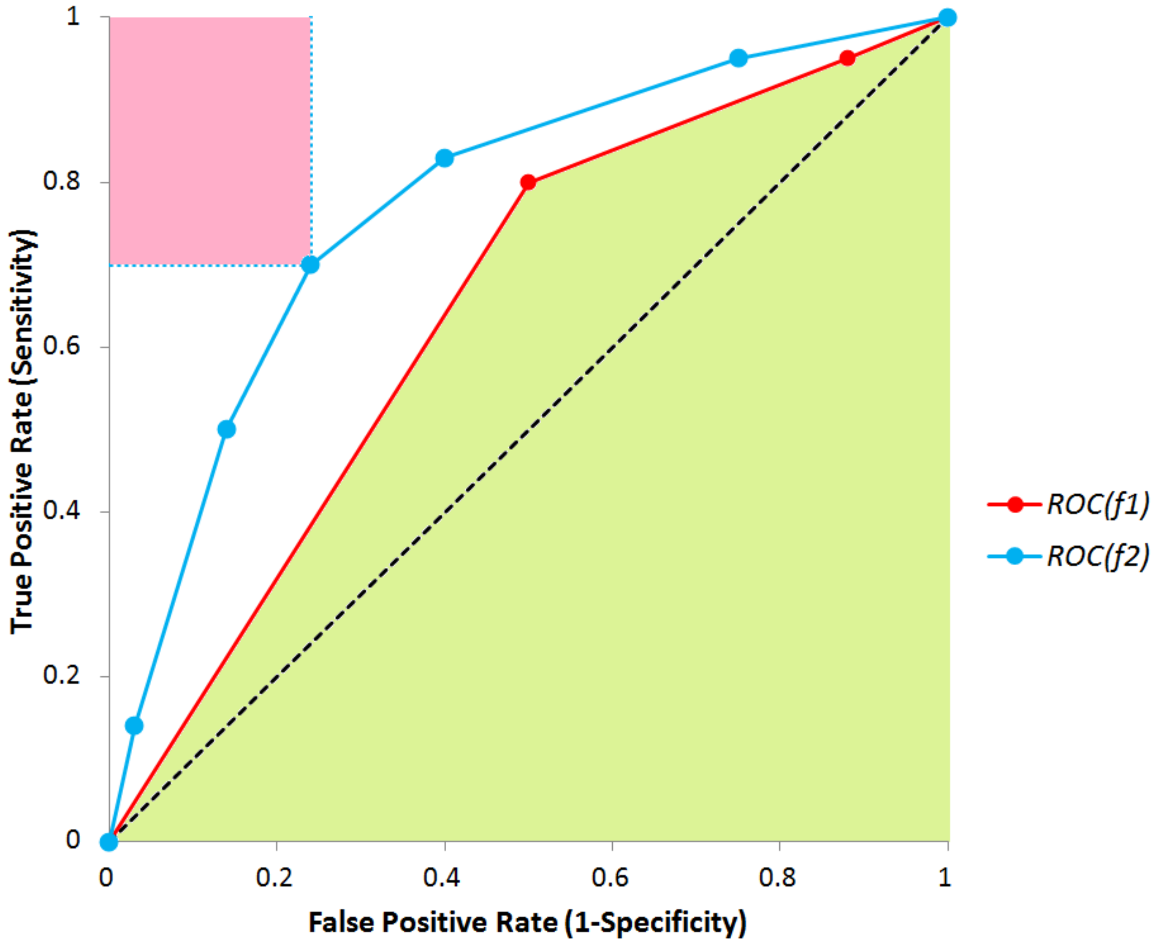

Figure 1: The principle for quantification of significance of ROC curve gain. In terms of this figure, first for each point on ROC(f_2) we are seeking the maximal probability mass accumulated above the point (i.e. the "pink" region) for sampling t_0 Controls and t_1 Cases from any population for which (true) (FPR, TPR) points fall into the "green" region below the ROC(f_1) curve. Then we choose the point from ROC(f_2) with the smallest such probability, and deem this probability to be the measure of the "gain" of f_2 over f_1.

$[0,1]^2$ which is the collection of all values of $\text{FPR}_{\mathcal{S}}$ and $\text{TPR}_{\mathcal{S}}$ achieved for all possible decision thresholds:

$$\begin{aligned}\text{ROC}_{\mathcal{S}}(f) &:= \big\{\big(\text{FPR}_{\mathcal{S}}(f,\theta),\ \text{TPR}_{\mathcal{S}}(f,\theta)\big)\ \big|\ \theta\in\mathbb{R}\big\}\ \cup\ \{(0,0)\}\\ &= \big\{\big(1-\text{SPE}_{\mathcal{S}}(f,\theta),\ \text{SEN}_{\mathcal{S}}(f,\theta)\big)\ \big|\ \theta\in\mathbb{R}\big\}\ \ \cup\ \{(0,0)\}\\ &\subset \mathbb{I}^2.\end{aligned}$$

In a typical case of interest here the set $\text{ROC}_{\mathcal{S}}(f)$ is finite, and will be viewed as a set of vertices of a piecewise linear, concave curve linking the vertices ordered by the corresponding values of parameter θ. Let $\mathcal{H}_0$ be a subset of $\mathbb{I}^2$. We formally define the $\mathcal{H}_0$*-significance* for $\text{ROC}(f)$ as the probability

$$P_{\mathcal{H}_0}(f) := \min_{\theta}\ \max_{(f',\theta'|\mathcal{P})\sim\mathcal{H}_0} \mathbf{P}_{\mathcal{S}_0,\mathcal{S}_1}\left[\text{SEN}_{\mathcal{S}}(f',\theta')\geq \text{SEN}_{\mathcal{S}}(f,\theta)\ \&\ \text{SPE}_{\mathcal{S}}(f',\theta')\geq \text{SPE}_{\mathcal{S}}(f,\theta)\right], \tag{1}$$

where the "min" is over all thresholds $\theta\in\mathbb{R}$; the "max" is over all selections of functions $f':\mathcal{P}\to\mathbb{R}$ and thresholds $\theta'\in\mathbb{R}$ such that the sensitivity and specificity for the *population* $\mathcal{P}$ belong to the region $\mathcal{H}_0$, which means that

$$\big(1-\text{SPE}_{\mathcal{P}}(f',\theta'),\text{SEN}_{\mathcal{P}}(f',\theta')\big)\in\mathcal{H}_0;$$

finally, the probability "$\mathbf{P}$" is for the sampling of t_0 Controls and t_1 Cases from such a population $\mathcal{P}$.

In the special case of when $\mathcal{H}_0\subset\mathbb{I}^2$ is the main diagonal, we shall use the notation

$$P_{\text{SS}}(f) := P_{\mathcal{H}_0}(f). \tag{2}$$

1.2 Prevalence mapping

Let $g:\mathcal{P}\to V$ be a function with a finite set of values $V=\{v_1,v_2,...,v_m\}$. This is a prototype of genotyping function, in particular, a SNP-probe with values in $V:=\{0,1,2\}$, or a pair of SNP-probes, $g=(g_1,g_2):\mathcal{P}\to\{0,1,2\}^2$ mapping $\mathcal{P}$ into the 9-element space $V=\{0,1,2\}^2=\{(0,0),(1,0),...,(2,2)\}$. With such a function we formally associate *the prevalence mapping* $f_{g|\mathcal{S}}:\mathcal{P}\to[0,1]$ defined as

$$f_{g|\mathcal{S}}(s) := \frac{|\{s'\in\mathcal{S}_1\mid g(s')=g(s)\}|}{|\{s'\in\mathcal{S}\mid g(s')=g(s)\}|} \tag{3}$$

and a *relative risk mapping* $r_{g|\mathcal{S}}:\mathcal{P}\to[0,\infty]$

$$r_{g|\mathcal{S}}(s) := \frac{t_0}{t_1}\times\ \frac{|\{s'\in\mathcal{S}_1\mid g(s')=g(s)\}|}{|\{s'\in\mathcal{S}_0\mid g(s')=g(s)\}|} \tag{4}$$

for every $s\in\mathcal{P}$. The mapping $f_{g|\mathcal{S}}$ is more natural in analysis of diseases while $r_{g|\mathcal{S}}$ is commonly used in analysis of contingency tables. Note that both the prevalence mapping and the relative risk mapping are

constant on the level sets $g^{-1}(v) \subset \mathcal{P}$ of the genotyping map $g : \mathcal{P} \to V$. Thus we can naturally extend both of them to mappings from V into $[0, 1]$ and V into $\mathbb{R}$. Thus we also have mappings $f_{g|\mathcal{S}} : V \to [0, 1]$ and $r_{g|\mathcal{S}} : V \to \mathbb{R}$ respectively. We have the following result.

Theorem 1 *(i) The curve $ROC_{\mathcal{S}}(g)$ is uniquely defined by the level sets $g^{-1}(v) \cap \mathcal{S}$, $v \in V$ and the permutation of the set V according to the decreasing values of the prevalence mapping $f_{g|\mathcal{S}} : V \to [0, 1]$, or equivalently of the relative risk mapping, $r_{g|\mathcal{S}} : V \to \mathbb{R}$.*
(ii) Each such ROC curve is a collection of vertices of the piecewise linear upper boundary of the convex hull of the cloud set *of g defined below*

$$\text{CLOUD}_{\mathcal{S}}(g) := \left\{ \left(\frac{|g^{-1}(V') \cap \mathcal{S}_0|}{t_0}, \frac{|g^{-1}(V') \cap \mathcal{S}_1|}{t_1} \right) \,\middle|\, V' \subset V \right\} \subset \mathbb{I}^2.$$

(iii) This ROC curve dominates every ROC curve for any deterministic function f of the genotyping calls, i.e. for any superposition of the form $f \circ g : \mathcal{S} \to \mathbb{R}$.

Thus the ROC curve for the prevalence mapping is canonically defined by the corresponding genotype mapping, and is the "best" ROC curve for any deterministic predictor of phenotype which factorises through this genotype mapping. The permutation of the finite set V induced by $f_{g|\mathcal{S}}$ as in (i) above is referred to as the *prevalence permutation of g.*

1.3 Tests for Gain of a Pair of Genotyping Probes.

In this section we formally introduce the set of filters used in this paper. They are defined in terms of probability values. Those values are very small in practice, and it is practical and convenient to use their negative logarithms. This way they are easily representable within the limited precision of computer hardware and align with our common language habits, i.e. that the "better" or "more significant" value is represented by a larger number. As an implicit rule we shall use the prefix "flt", the first three consonants of word "filter", as a substitute for "$-\log_{10}$".

Let us consider a pair of probes $g_i : \mathcal{P} \to V$, $i = 1, 2$. We denote by $f_{g_i|\mathcal{S}} : \mathcal{P} \to [0, 1]$, $i = 1, 2$, the prevalence mapping for individual probes and by $f_{g_1,g_2|\mathcal{S}} : \mathcal{P} \to [0, 1]$ the prevalence mapping for the pair.

We define the *gain in sensitivity and specificity* filter for a probe-pair

$$\text{flt}_{\text{GSS}}(g_1, g_2) := -\log_{10} P_{\mathcal{H}_0}(f_{g_1,g_2|\mathcal{S}}),$$

where $P_{\mathcal{H}_0}$ is defined by (1) for

$$\mathcal{H}_0 := \text{CONV}\big(\text{ROC}_{\mathcal{S}}(f_{g_1|\mathcal{S}}) \cup \text{ROC}_{\mathcal{S}}(f_{g_2|\mathcal{S}})\big) \subset \mathbb{I}^2$$

which is the smallest convex set containing both ROC curves for individual probes.

This is our main filter for detection of epistasis. However, it is not well suited for scanning of large numbers of probe pairs, since the set $\mathcal{H}_0$ is intrinsically dependent on the pair of probes (g_1, g_2). In order to deal with this limitation we introduce the following heuristic filter which is computationally less expensive, yet is a very good proxy for GSS. We define the *differential sensitivity-specificity* (DSS) *filter* for the probe-pair via the ratio of probabilities

$$\begin{aligned}\mathrm{flt}_{\mathrm{DSS}}(g_1, g_2|\mathcal{S}) &:= -\log_{10} \frac{P_{\mathrm{SS}}(f_{g_1,g_2|\mathcal{S}})}{\min(P_{\mathrm{SS}}(f_{g_1|\mathcal{S}}), P_{\mathrm{SS}}(f_{g_2|\mathcal{S}})} \\ &= \mathrm{flt}_{\mathrm{SS}}(g_1, g_2|\mathcal{S}) - \max\left(\mathrm{flt}_{\mathrm{SS}}(g_1|\mathcal{S}), \mathrm{flt}_{\mathrm{SS}}(g_2|\mathcal{S})\right),\end{aligned}$$

where P_{SS} and $\mathrm{flt}_{\mathrm{SS}}$ are defined by (2) and $-\log_{10} P_{\mathrm{SS}}$, respectively.

We have the following general result

Theorem 2

$$\mathrm{flt}_{\mathrm{SS}}(f_{g_1,g_2}) \geq \max\big(\mathrm{flt}_{\mathrm{DSS}}(f_{g_1,g_2}), \mathrm{flt}_{\mathrm{GSS}}(f_{g_1,g_2})\big).$$

Historical Context

In the context of the century-old Pearson-Yule Association Controversy vividly summarised by Agresti [2, Chapter 11], our approach and philosophy is clearly on Yule's side. Our statistics are derived exactly from discrete distributions without assumptions or the use of any approximated continuous distributions. We must emphasise however, that our motivations are predominantly practical as the number of samples in modern GWAS (tens of thousands of samples) and the billions of SNP pairs evaluated in exhaustive bivariate analysis invalidate the continuous approximations underlying Pearson's original approach. To control the large multiple choice correction, we need to examine the far tails of the distributions under and in these regions the central limit theorem justifying "large sample" approximations loses its validity. Thus either the theorem has to be amended to cover such situations, or the far tails of distributions need to be evaluated directly. In this paper we have taken the latter approach and have shown that it yields meaningful results.

1.4 Computational implementation

Efficient implementation of computation of $P_{\mathcal{H}_0}$ is critical for implementation of all the above filters. We have the following chain of equalities.

$$P_{\mathcal{H}_0}(f) = \min_{(\xi_0,\xi_1)\in\text{ROC}_{\mathcal{S}}(f)} \max_{(f',\theta'|\mathcal{P})\sim\mathcal{H}_0} \mathbf{P}_{\mathcal{S}_0,\mathcal{S}_1}\left[\text{SEN}_{\mathcal{S}}(f',\theta') \geq \xi_1 \;\&\; \text{SPE}_{\mathcal{S}}(f',\theta') \geq 1-\xi_0\right] \quad (5)$$

$$= \min_{(\xi_0,\xi_1)\in\text{ROC}_{\mathcal{S}}(f)} \max_{(p_0,p_1)\in\mathcal{H}_0} \mathbf{P}\left[\frac{X_1}{t_1} \geq \xi_1 \;\&\; \frac{X_0}{t_0} \leq \xi_0 \;\middle|\; X_i \sim Bi(p_i,t_i),\; i=0,1\right] \quad (6)$$

$$= \min_{(\xi_0,\xi_1)\in\text{ROC}_{\mathcal{S}}(f)} \max_{(p_0,p_1)\in\mathcal{H}_0} \Phi(p_0,p_1|\xi_0,\xi_1), \quad (7)$$

where

$$\Phi(p_0,p_1|x_0,x_1) := \sum_{i=0}^{x_0}\binom{t_0}{i}p_0^i(1-p_0)^{t_0-i}\sum_{j=x_1}^{t_1}\binom{t_1}{j}p_1^j(1-p_1)^{t_1-j} \quad (8)$$

for $0 \leq p_0, p_1 \leq 1$ and $0 \leq x_i \leq t_i$ for $i = 0, 1$.

The function (8) has a number of properties simplifying computation of its maximum.

Theorem 3 *(i) If subset $\mathcal{H}_0 \subset \mathbb{I}^2$ is closed, than the $\max_{(p_0,p_1)\in\mathcal{H}_0}$ is achieved on (topological) boundary of $\mathcal{H}_0$.*

(ii) If $\mathcal{H}_0$ is convex, then any local maximum of Φ on $\mathcal{H}_0$ is its unique global maximum.

As a consequence of the uniqueness outlined above, the maximum of Φ on $\mathcal{H}_0$ can be found by considering its restriction to the boundary and application of iterative procedures, which are guaranteed to converge to the solution. However, any practical solution needs to deal with numerical overflow and underflow, since terms under summation in (8) involve quantities of such small magnitude and ratios of quantities of such large magnitude that they cannot be represented in computer memory. This requires careful handling and results in some other simplifications and dedicated algorithms. We intend to discuss this in detail in future work.

Note that in the case of P_{SS} and P_{DSS} we need to deal only with the boundary of $\mathcal{H}_0$ which is simply the main diagonal of the unit square $\mathbb{I}^2$. In the case of GSS the relevant boundary is more complex: the piecewise linear upper boundary of $\text{CONV}\left(\text{ROC}_{\mathcal{S}}(f_{g_1}) \cup \text{ROC}_{\mathcal{S}}(f_{g_2})\right)$.

1.5 QQ plots

In Figure 2 of this section we show QQ-plots for the filters used in the paper. The x-axis shows $-\log_{10}$ of observed cumulative frequency, y-axis shows $-\log_{10}$ of p-values allocated by various filters. The data for three main filters, flt_{χ^2}, flt_{DSS} and flt_{SS} was collected for all $\approx 10^{11}$ SNP-pairs. For flt_{GSS} however, we used

only a small fraction of all pairs. Namely, for each study we have used $\approx 10^7$ random pairs (the solid red lines in the plots) and the subset of top few thousand pairs preselected by the other filters (red broken line), each then evaluated by $\mathrm{flt}_{\mathrm{GSS}}$. Ideally, each plot should follow the diagonal line at the initial stage, then should dramatically rise, which corresponds to a relatively small number of discovered interacting SNP-pairs. The $\mathrm{flt}_{\mathrm{GSS}}$ an $\mathrm{dflt}_{\mathrm{DSS}}$ are the closest to that ideal, though results for $\mathrm{flt}_{\mathrm{GSS}}$ are based on a very small fraction of the population only (10^7 constitutes merely 0.001% of the total number of SNP-pairs in this data). The plots for $\mathrm{flt}_{\mathrm{SS}}$ and flt_{χ^2} show that those filters allocate grossly inflated "p-values" leading to excess false-positive results. Note that the same applies to the popular Fisher Exact test, which allocates p-values virtually the same as $\mathrm{flt}_{\mathrm{SS}}$, see Supplementary Methods, Figure 2. The empirical evidence provided by the seven GWAS datasets indicates that the popular χ^2 and Fisher Exact tests poorly match the dependencies introduced by interactions in the real GWAS datasets and should be considered with extreme care when applied to inference of epistasis in GWAS.

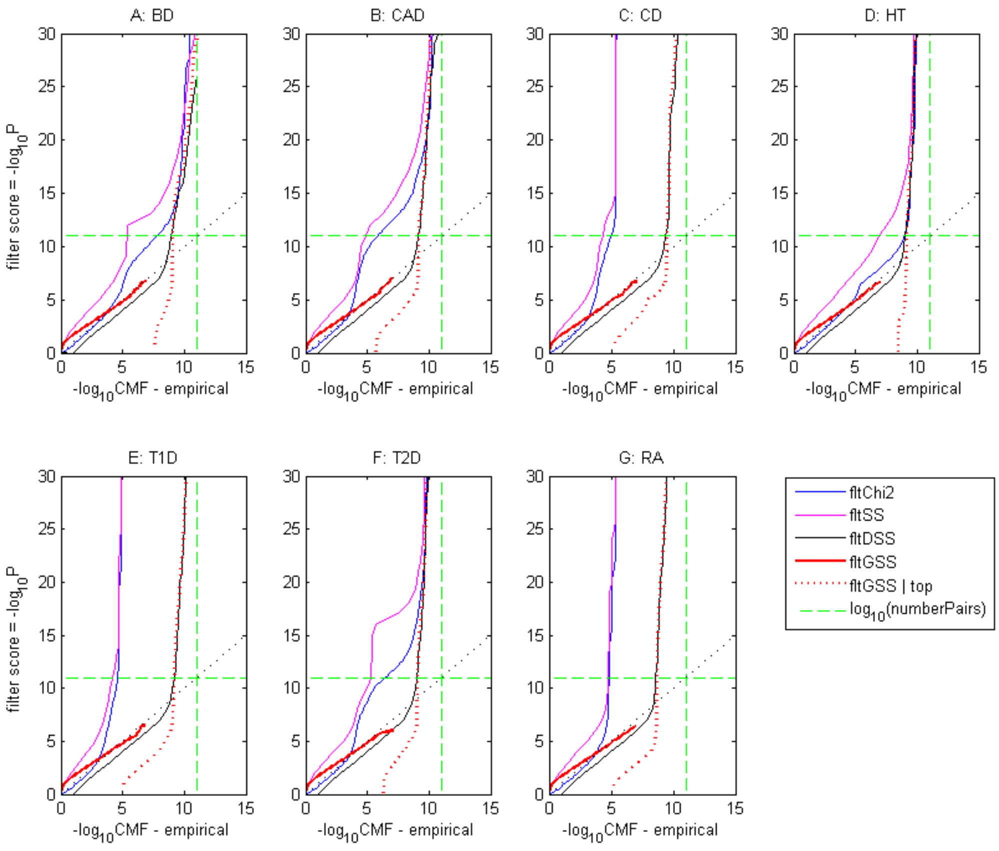

Figure 2: QQ-plots for the filters used in the paper.

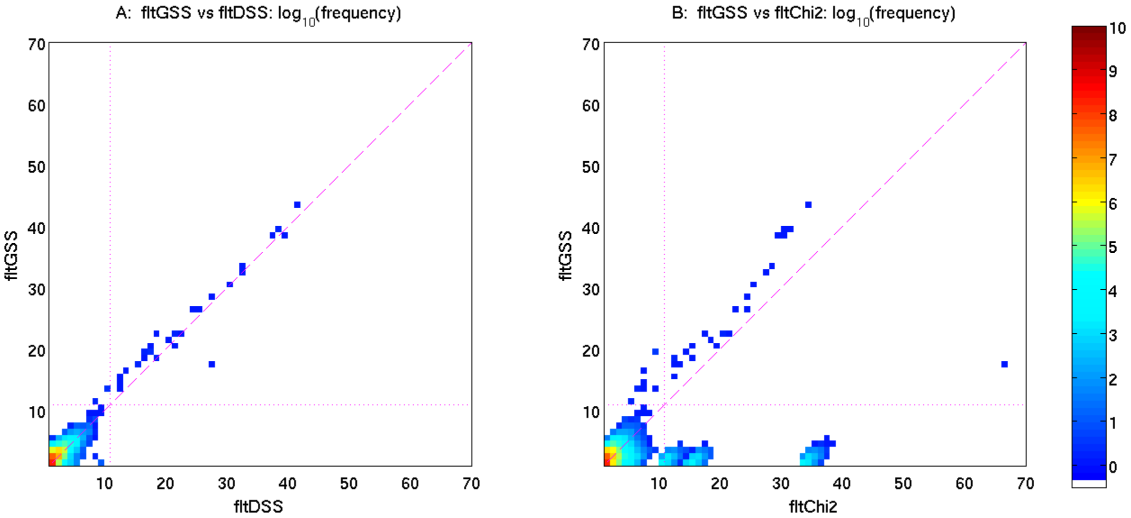

Figure 3: Plot of $\mathrm{flt}_{\mathrm{GSS}}$ versus $\mathrm{flt}_{\mathrm{DSS}}$ and flt_{χ^2} for an exhaustive evaluation of all pairs for 28063 SNPs on Chromosome 6 for rheumatoid arthritis (RA-data). As in Figure 4 we observe a remarkable concordance between $\mathrm{flt}_{\mathrm{DSS}}$ and $\mathrm{flt}_{\mathrm{GSS}}$ filters in plot (a), however this is not the case for comparison of $\mathrm{flt}_{\mathrm{DSS}}$ and flt_{χ^2}. In accordance with results shown in Figure 1.a of the paper, filter flt_{χ^2} shows a number of spurious "significant" associations caused by main effects.

1.6 Comparison of Filters

In Figure 4 we show scatterplot for comparing $\mathrm{flt}_{\mathrm{DSS}}$ and $\mathrm{flt}_{\mathrm{GSS}}$ for top 500K pair preselected by $\mathrm{flt}_{\mathrm{DSS}}$. In Figure 3 we compare $\mathrm{flt}_{\mathrm{GSS}}$ with $\mathrm{flt}_{\mathrm{DSS}}$ and flt_{χ^2} for an exhaustive evaluation of all pairs for 28063 SNPs on chromosome 6 for rheumatoid arthritis. This evaluation used our Matlab implementation used in this paper capable of evaluation of 10,000 pairs per minute. The computation used 600 CPU hours, With this efficiency it is required 150,000 CPU hours $\approx$ 17 CPUyears to repeat this computation for a single WTCCC study which uses 450K SNPs. This is impractical and in order to deal with this bottleneck we have developed DSS test for first stage of filtering.

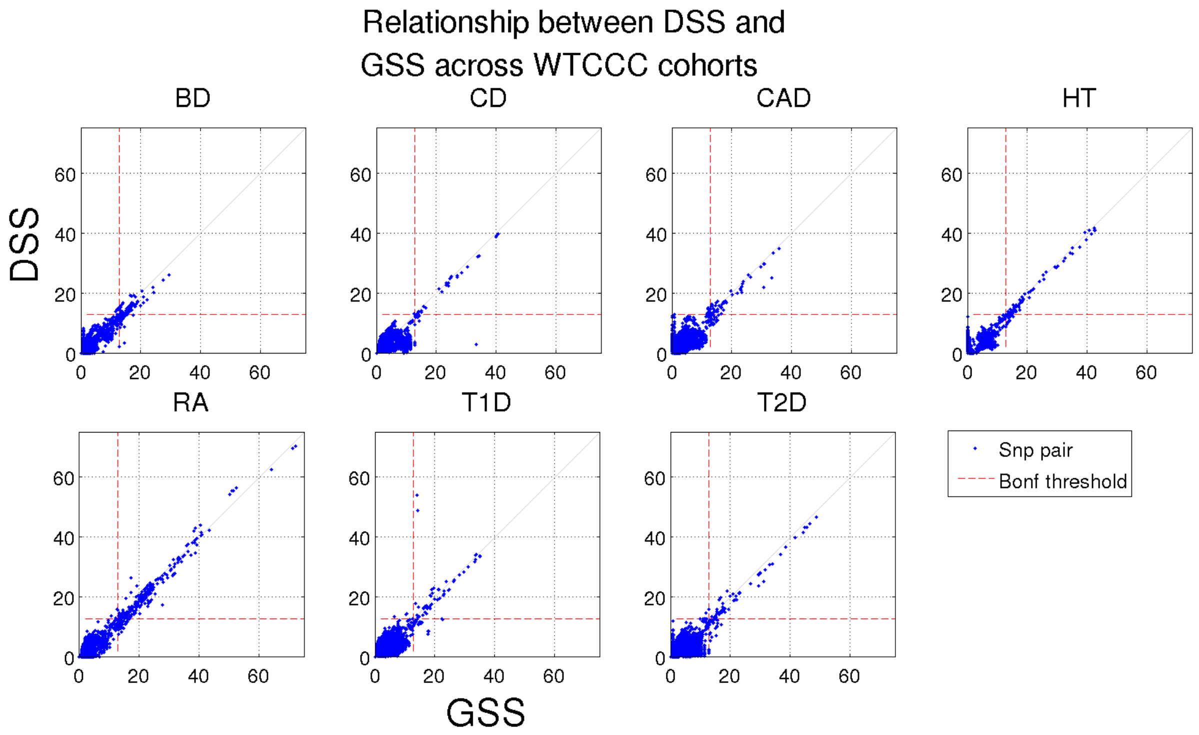

Figure 4: Plot of flt_{GSS} versus flt_{DSS} for seven WTCCC diseases for the top $500K$ pairs preselected by flt_{DSS}. We observe a remarkable concordance between both filters, especially for the critical values above Bonferroni threshold ($\approx \log_{10} \binom{n_{snp}}{2} \approx 11$), marked by red lines). This shows that flt_{DSS} is a reasonable proxy for the more expensive flt_{GSS}.

2 Calculation of Timing

To compare the computation times of the different methods, we estimated the running times from the times reported in literature. We scaled the reported time such that it is comparable in terms of the size of the dataset and the computational platform. The rightmost column in Table 1 is obtained using the following formulas.

Formula for CPU (current WTCCC data, 450K probes, 5K samples)

$$T_{adj} = \left(\frac{450K}{n_{SNP}}\right)^2 \times \frac{5000}{n_{sample}} \times \frac{n_{cores}}{4} \times \frac{f_{clock}}{3GHz} \times T_{rep}$$

Formula for GPU (current WTCCC data, 450K probes, 5K samples)

$$T_{adj} = \left(\frac{450K}{n_{SNP}}\right)^2 \times \frac{5000}{n_{sample}} \times \frac{n_{cores}}{448} \times \frac{f_{clock}}{1.215GHz} \times T_{rep}$$

Formula for CPU (forthcoming GWAS data, 5M probes, 10K samples)

$$T_{adj} = \left(\frac{5M}{n_{SNP}}\right)^2 \times \frac{10000}{n_{sample}} \times \frac{n_{cores}}{4} \times \frac{f_{clock}}{3GHz} \times T_{rep}$$

Formula for GPU (forthcoming GWAS data, 5M probes, 10K samples)

$$T_{adj} = \left(\frac{5M}{n_{SNP}}\right)^2 \times \frac{10000}{n_{sample}} \times \frac{n_{cores}}{448} \times \frac{f_{clock}}{1.215GHz} \times T_{rep}$$

Method	n_{SNP}	n_{sample}	n_{cores}	f_{clock} [GHz]	T_{rep} [hours]	T_{adj} (current) [hours]	T_{adj} (future) [hours]
BOOST	360000	5000	1	3	60	23	5787
PLINK	89294	5000	1	3	336	2133	526750
GBOOST	351542	5003	240	1.476	1.34	1.42	352
EpiGPU	300000	1000	512	1.544	1.05	17	4236
SHEsisEPI	500000	5000	480	1.476	27	28	7028
EPIBLASTER	500000	2000	1920	1.242	24	213	52571

Table 1: Computation time (reported and estimated) from literature. BOOST and PLINK are CPU based methods and the others are GPU based methods. All numbers were obtained from the respective publications [3–7] except for PLINK which was obtained from [8].

3 Scaling of GWIS of implementations

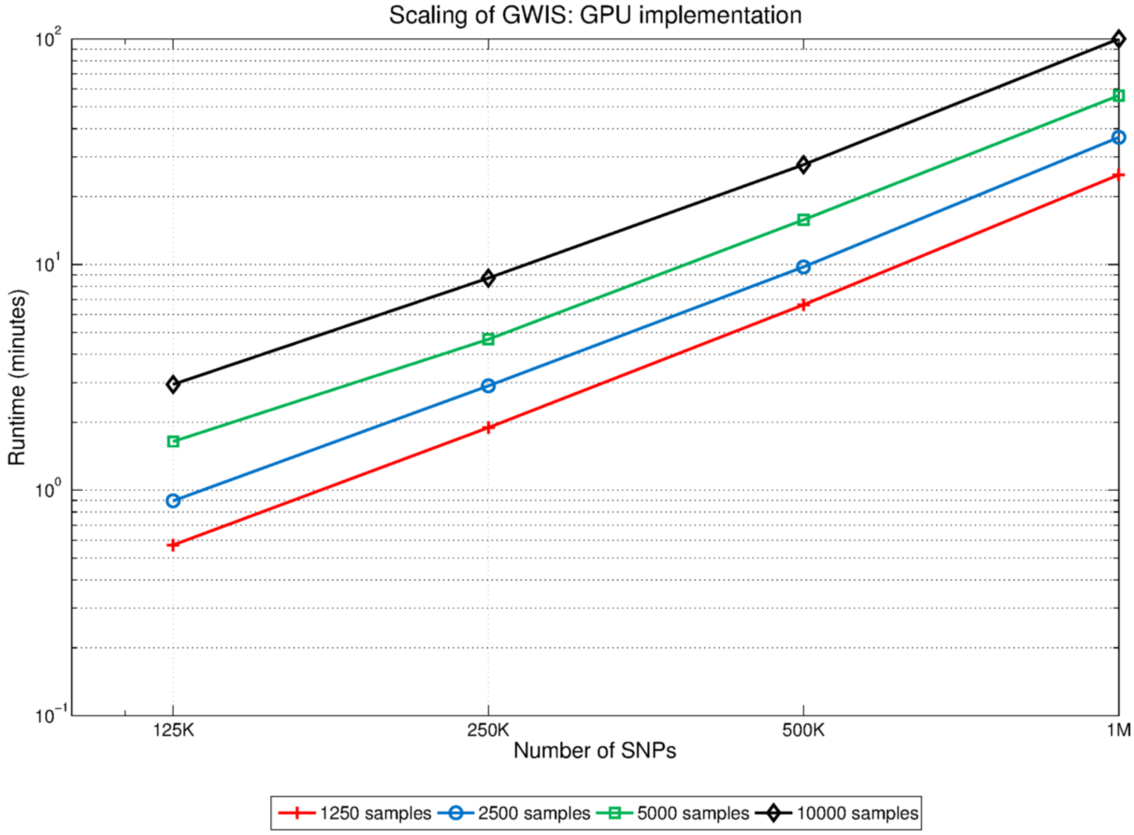

Figure 5: Scaling of runtime as the number of SNPs (x-axis) and samples (different lines) are increased. The linearity of the lines on the logaritmic graph scales shows that the runtime scales quadratically with the number of SNP (eg linearly with the number of possible SNP pairs) while the even spacing between the different series shows that the runtime scales linearly with the number of samples.

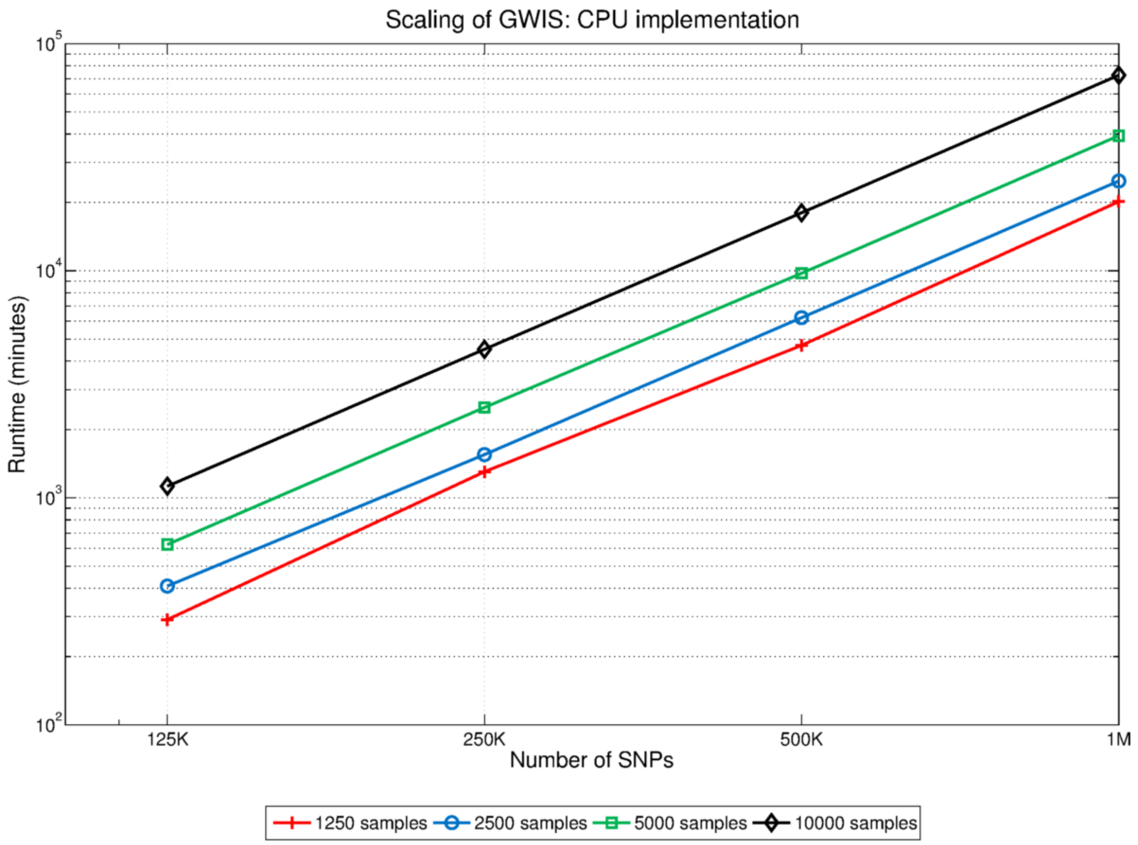

Figure 6: Scaling of runtime as the number of SNPs (x-axis) and samples (different lines) are increased. The linearity of the lines on the logaritmic graph scales shows that the runtime scales quadratically with the number of SNP (eg linearly with the number of possible SNP pairs) while the even spacing between the different series shows that the runtime scales linearly with the number of samples.

4 Representative SNP Pairs

We show here representative examples of epistatic interactions from the final list of 682 pairs selected by flt_{GSS} in the seven WTCCC datasets. These are statistically significant associations in the data that *need further screening*. The first stage of such a screen should involve a check for genotyping errors, including analysis of intensity plots that determine the genotype calls, see [9] [10].

Each example is a pair of SNPs that combined display enhanced protective (from disease) or contributing (to disease) effects - or both. In each figure, three panels show prevalence mapping ROC curves for both the pair (red) and individual SNPs (blue, green). Panels (b) and (c) zoom into the protective (top-right 20%) and contributory (lower-left 20%) areas respectively.

Panel (d) shows selected statistics for the pair of SNPs. The nine rows correspond to possible genotype calls for the pair of SNPs. Columns are: flt_{SS} - the sensitivity-specificity filter score; flt_{GSS} - the gain filter score; OR - Odds-Ratio; $p0$ - percentage of Controls for the genotype call in this row; $p1$ - percentage of Cases; spe% - specificity %; sen% - sensitivity %; RelRisk - relative risk for the genotype call $:= p1/p0$; g_1, g_2 - genotype calls for the pair of SNPs; prevPerm - prevalence permutation (see Supplement Section 1.2).

The pairs shown were chosen for the range of protective and contributory effects observed across a range of diseases. Figure 7 shows both protective and contributory effects in CAD. Figure 8 shows only protective effects in CD. Figure 9 shows both protective and contributory effects in HT in the absence of strong univariate association. Figure 10 shows only contributory effects in RA. Finally, Figures 11 and 12 show bivariate epistatic modifications of a strong univariate SNPs in T1D and CD.

Figure 4a and Equation 2 describe how we are optimising the statistical difference between ROC curves. This is different from purely maximising sensitivity and specificity and an example of this can be seen in Supplementary Figure 11. Here we see that the optimal points as chosen by GSS, are driven by genotype combinations as indicated by rows one and nine, while the maximal sensitivity and specificity correspond to row 4 (spec=62.5 and sens 71.52). Similar comments can be made about figure 12 (rows 2 and 9 are optimal for GSS while row 5 is optimal for specificity and sensitivity).

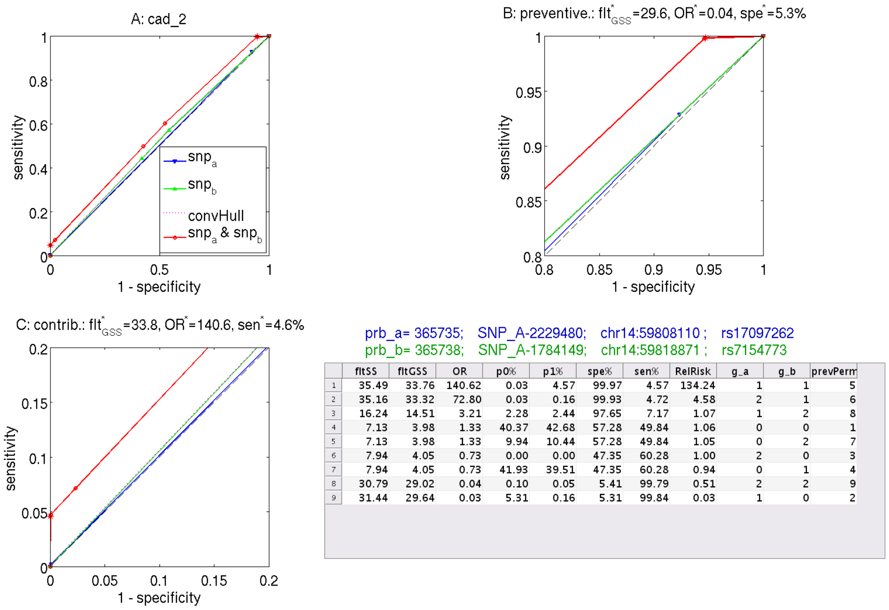

	fltSS	fltGSS	OR	p0%	p1%	spe%	sen%	RelRisk	g_a	g_b	prevPerm
1	35.49	33.76	140.62	0.03	4.57	99.97	4.57	134.24	1	1	5
2	35.16	33.32	72.80	0.03	0.16	99.93	4.72	4.58	2	1	6
3	16.24	14.51	3.21	2.28	2.44	97.65	7.17	1.07	1	2	8
4	7.13	3.98	1.33	40.37	42.68	57.28	49.84	1.06	0	0	1
5	7.13	3.98	1.33	9.94	10.44	57.28	49.84	1.05	0	2	7
6	7.94	4.05	0.73	0.00	0.00	47.35	60.28	1.00	2	0	3
7	7.94	4.05	0.73	41.93	39.51	47.35	60.28	0.94	0	1	4
8	30.79	29.02	0.04	0.10	0.05	5.41	99.79	0.51	2	2	9
9	31.44	29.64	0.03	5.31	0.16	5.31	99.84	0.03	1	0	2

Figure 7: Epistatic interactions of a pair of SNPs showing both protective and contributory effects, CAD data. Note both SNPs have insignificant discrimination capabilities on their own. For a full description of this figure, see Supplementary Section 4, "Representative SNP Pairs".

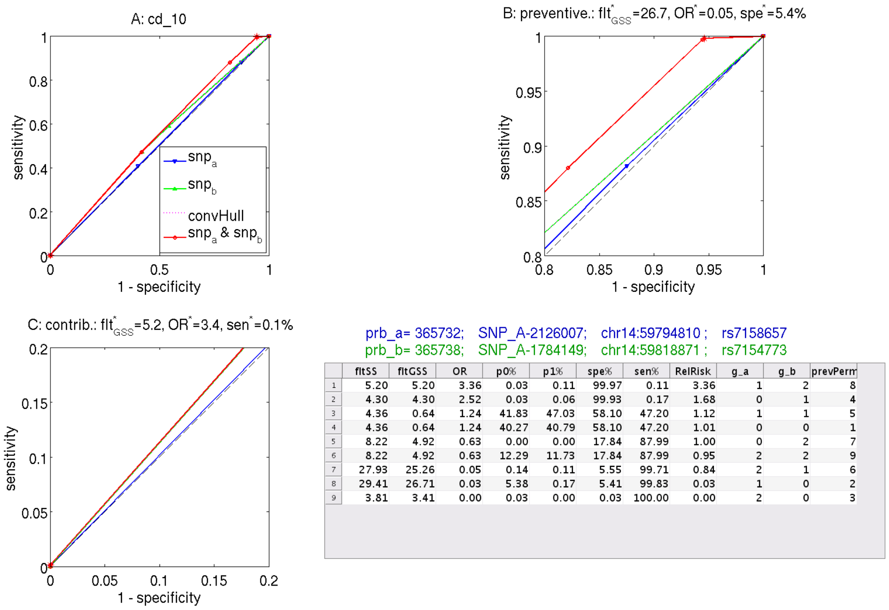

	fltSS	fltGSS	OR	p0%	p1%	spe%	sen%	RelRisk	g_a	g_b	prevPerm
1	5.20	5.20	3.36	0.03	0.11	99.97	0.11	3.36	1	2	8
2	4.30	4.30	2.52	0.03	0.06	99.93	0.17	1.68	0	1	4
3	4.36	0.64	1.24	41.83	47.03	58.10	47.20	1.12	1	1	5
4	4.36	0.64	1.24	40.27	40.79	58.10	47.20	1.01	0	0	1
5	8.22	4.92	0.63	0.00	0.00	17.84	87.99	1.00	0	2	7
6	8.22	4.92	0.63	12.29	11.73	17.84	87.99	0.95	2	2	9
7	27.93	25.26	0.05	0.14	0.11	5.55	99.71	0.84	2	1	6
8	29.41	26.71	0.03	5.38	0.17	5.41	99.83	0.03	1	0	2
9	3.81	3.41	0.00	0.03	0.00	0.03	100.00	0.00	2	0	3

Figure 8: Epistatic interactions of a pair of SNPs showing both protective effect only, CD data. For a full description of this figure, see Supplementary Section 4, "Representative SNP Pairs". Here, only significantly protective effects are produced by the pair of SNPs.

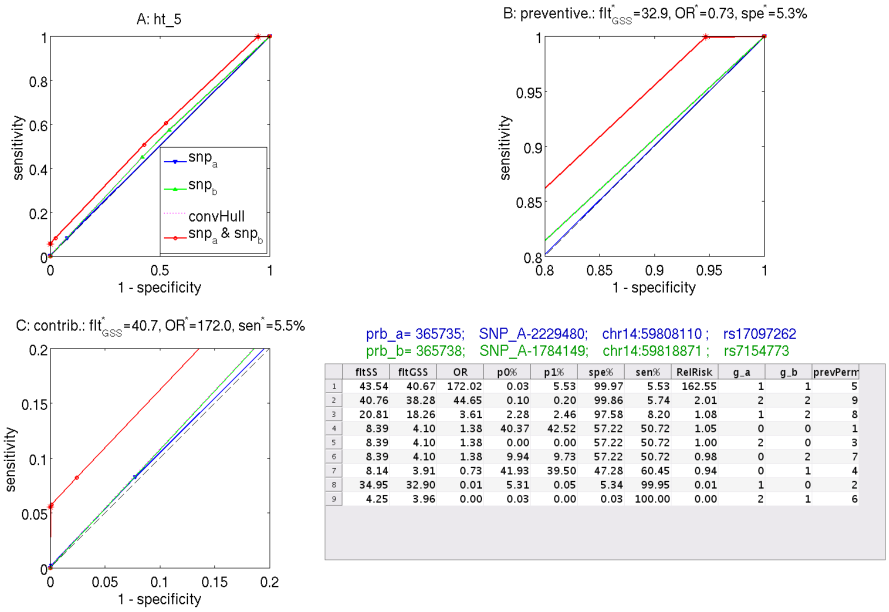

	fltSS	fltGSS	OR	p0%	p1%	spe%	sen%	RelRisk	g_a	g_b	prevPerm
1	43.54	40.67	172.02	0.03	5.53	99.97	5.53	162.55	1	1	5
2	40.76	38.28	44.65	0.10	0.20	99.86	5.74	2.01	2	2	9
3	20.81	18.26	3.61	2.28	2.46	97.58	8.20	1.08	1	2	8
4	8.39	4.10	1.38	40.37	42.52	57.22	50.72	1.05	0	0	1
5	8.39	4.10	1.38	0.00	0.00	57.22	50.72	1.00	2	0	3
6	8.39	4.10	1.38	9.94	9.73	57.22	50.72	0.98	0	2	7
7	8.14	3.91	0.73	41.93	39.50	47.28	60.45	0.94	0	1	4
8	34.95	32.90	0.01	5.31	0.05	5.34	99.95	0.01	1	0	2
9	4.25	3.96	0.00	0.03	0.00	0.03	100.00	0.00	2	1	6

Figure 9: Epistatic interactions of a pair of SNPs showing both protective and contributory effects, HT data. For a full description of this figure, see Supplementary Section 4, “Representative SNP Pairs”.

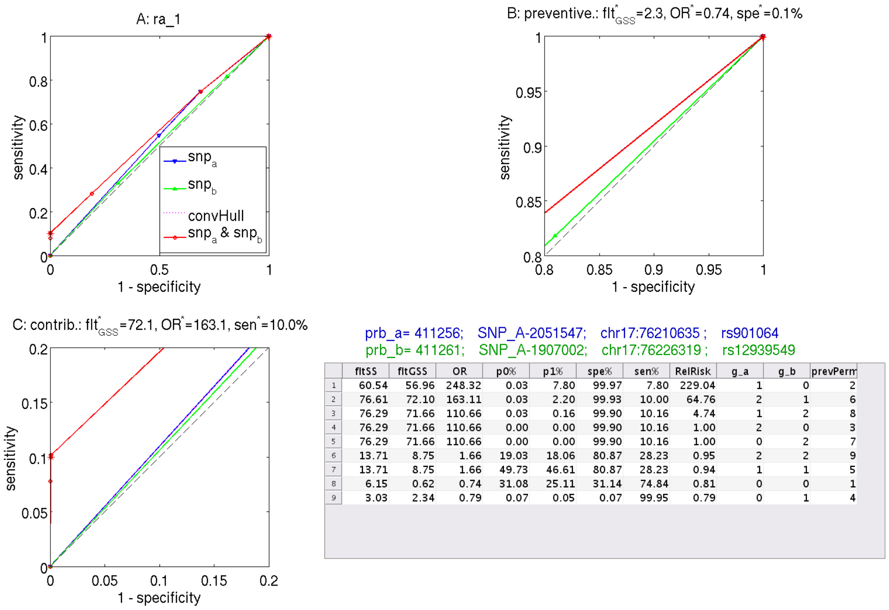

	fltSS	fltGSS	OR	p0%	p1%	spe%	sen%	RelRisk	g_a	g_b	prevPerm
1	60.54	56.96	248.32	0.03	7.80	99.97	7.80	229.04	1	0	2
2	76.61	72.10	163.11	0.03	2.20	99.93	10.00	64.76	2	1	6
3	76.29	71.66	110.66	0.03	0.16	99.90	10.16	4.74	1	2	8
4	76.29	71.66	110.66	0.00	0.00	99.90	10.16	1.00	2	0	3
5	76.29	71.66	110.66	0.00	0.00	99.90	10.16	1.00	0	2	7
6	13.71	8.75	1.66	19.03	18.06	80.87	28.23	0.95	2	2	9
7	13.71	8.75	1.66	49.73	46.61	80.87	28.23	0.94	1	1	5
8	6.15	0.62	0.74	31.08	25.11	31.14	74.84	0.81	0	0	1
9	3.03	2.34	0.79	0.07	0.05	0.07	99.95	0.79	0	1	4

Figure 10: Epistatic interactions of a pair of SNPs showing both protective and contributory effects, RA data. For a full description of this figure, see Supplementary Section 4, “Representative SNP Pairs”.

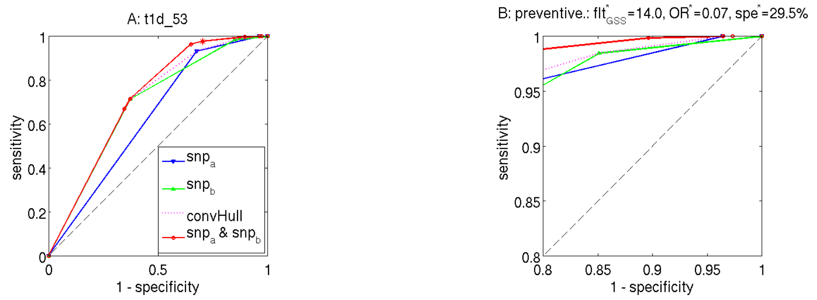

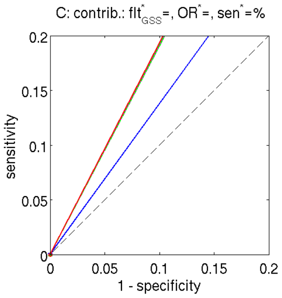

prb_a= 173423; SNP_A-2185852; chr6 :32703061 ; rs3129768
prb_b= 173424; SNP_A-1842324; chr6 :32712350 ; rs9272346

	fltSS	fltGSS	OR	p0%	p1%	spe%	sen%	RelRisk	g_a	g_b	prevPerm
1	111.50	0.81	0.26	34.65	66.94	65.35	66.94	1.93	0	0	1
2	111.50	0.81	0.26	2.72	4.58	65.35	66.94	1.68	1	0	2
3	125.21	0.71	0.24	27.67	24.96	62.63	71.52	0.90	0	1	4
4	178.50	12.55	0.07	5.45	1.38	34.96	96.48	0.25	0	2	7
5	161.18	13.97	0.05	19.13	1.99	29.51	97.86	0.10	1	1	5
6	66.42	5.02	0.01	6.77	0.15	10.38	99.85	0.02	1	2	8
7	25.11	0.41	0.00	0.10	0.00	3.61	100.00	0.00	2	0	3
8	24.32	0.29	0.00	0.85	0.00	3.51	100.00	0.00	2	1	6
9	18.58	0.00	0.00	2.65	0.00	2.65	100.00	0.00	2	2	9

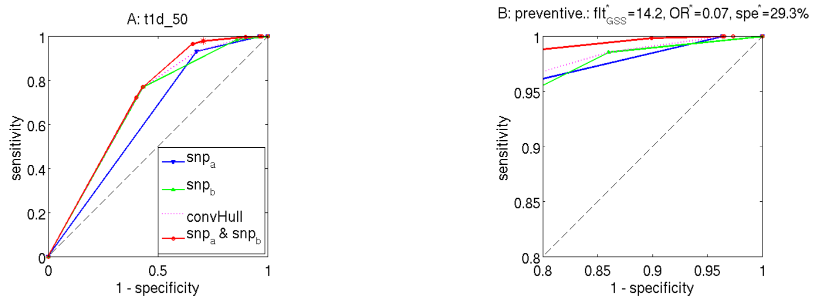

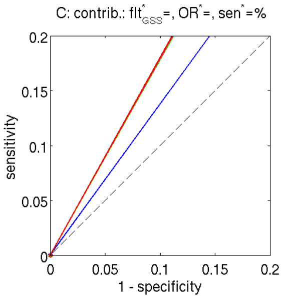

prb_a= 173423; SNP_A-2185852; chr6 :32703061 ; rs3129768
prb_b= 173425; SNP_A-4206711; chr6 :32717405 ; rs9272723

	fltSS	fltGSS	OR	p0%	p1%	spe%	sen%	RelRisk	g_a	g_b	prevPerm
1	111.86	0.86	0.26	40.23	72.39	59.77	72.39	1.80	0	0	1
2	111.86	0.86	0.26	2.96	4.64	59.77	72.39	1.57	1	0	2
3	126.82	0.74	0.23	22.67	19.61	56.81	77.02	0.87	0	1	4
4	174.64	12.17	0.07	4.87	1.27	34.14	96.64	0.26	0	2	7
5	160.35	14.24	0.05	19.20	1.94	29.27	97.91	0.10	1	1	5
6	64.25	4.83	0.01	6.47	0.15	10.07	99.85	0.02	1	2	8
7	25.11	0.41	0.00	0.14	0.00	3.61	100.00	0.00	2	0	3
8	24.06	0.26	0.00	0.82	0.00	3.47	100.00	0.00	2	1	6
9	18.58	0.00	0.00	2.65	0.00	2.65	100.00	0.00	2	2	9

Figure 11: Epistatic interaction of a univariately "strong" SNP with a much "weaker" partner, in T1D data. The pair shows both strong protective and contributory effects. These SNPs are apced only $\approx$ 10K bp apart. For a full description of this figure, see Supplementary Section 4, "Representative SNP Pairs". Note that these pairs have extreme flt_{SS} values, $= 178.50$ and $= 174.64$, respectively. They corresponding to the most extreme a red circles in the Supplementary Figure 18. Note that the first pair segregates 34.96% of controls with extreme odds ratio ≈ 0.05 and this is generating such an extreme p-value see Row 5 of the table in the figure). In the following plots we show another SNP-pair form this region showing equally impressive segregation capabilities. This will warrants follow up investigation, in particular, a quality control check for genotype calls.

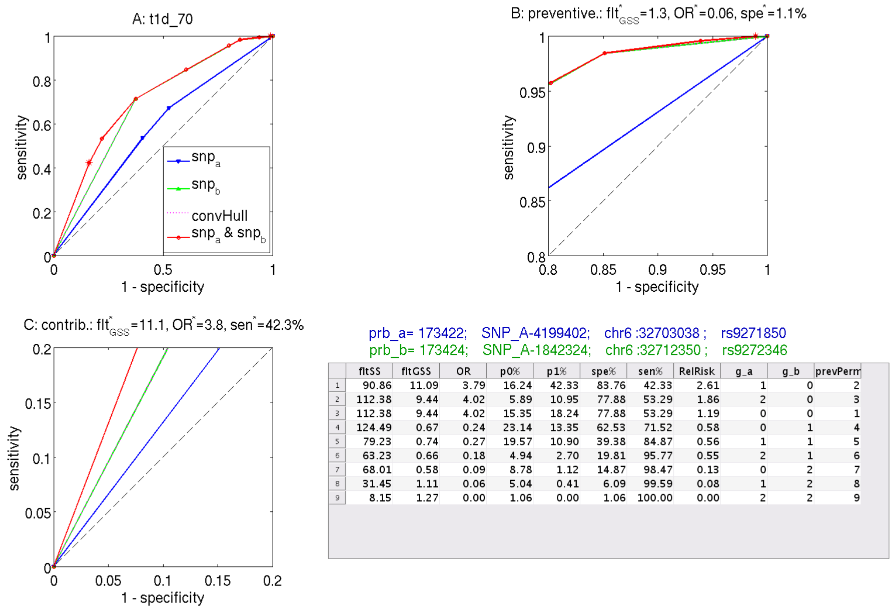

	fltSS	fltGSS	OR	p0%	p1%	spe%	sen%	RelRisk	g_a	g_b	prevPerm
1	90.86	11.09	3.79	16.24	42.33	83.76	42.33	2.61	1	0	2
2	112.38	9.44	4.02	5.89	10.95	77.88	53.29	1.86	2	0	3
3	112.38	9.44	4.02	15.35	18.24	77.88	53.29	1.19	0	0	1
4	124.49	0.67	0.24	23.14	13.35	62.53	71.52	0.58	0	1	4
5	79.23	0.74	0.27	19.57	10.90	39.38	84.87	0.56	1	1	5
6	63.23	0.66	0.18	4.94	2.70	19.81	95.77	0.55	2	1	6
7	68.01	0.58	0.09	8.78	1.12	14.87	98.47	0.13	0	2	7
8	31.45	1.11	0.06	5.04	0.41	6.09	99.59	0.08	1	2	8
9	8.15	1.27	0.00	1.06	0.00	1.06	100.00	0.00	2	2	9

Figure 12: Epistatic interaction of a univariately "strong" SNP with a much "weaker" partner, in T1D data. The pair shows both strong protective and contributory effects. These SNPs are spaced only $\approx$ 10K bp apart. For a full description of this figure, see Supplementary Section 4, "Representative SNP Pairs". Note that this pair has extreme $\mathrm{flt}_{\mathrm{SS}} = 124.49$, however, a red circle representing it in the Supplementary Figure 18 is masked by 'univariate' blue dot.

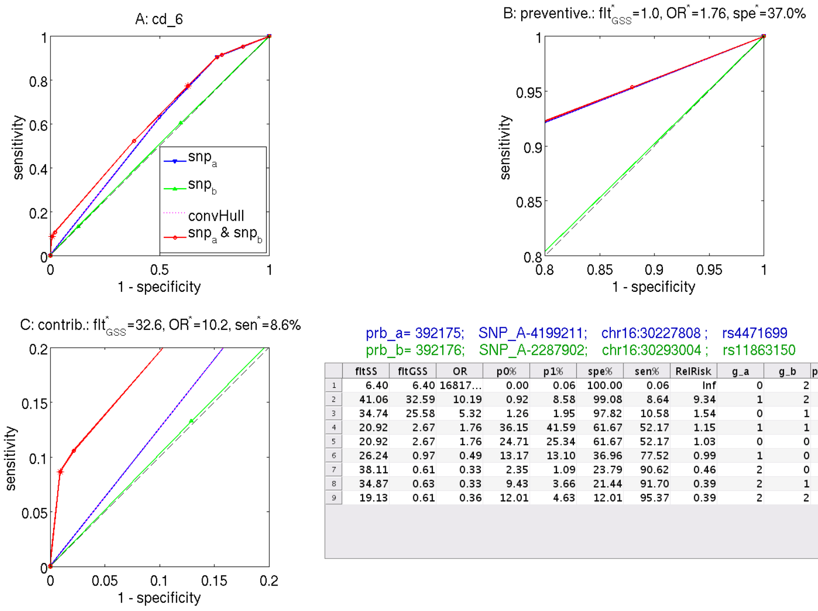

	fltSS	fltGSS	OR	p0%	p1%	spe%	sen%	RelRisk	g_a	g_b	prevPerm
1	6.40	6.40	16817...	0.00	0.06	100.00	0.06	Inf	0	2	7
2	41.06	32.59	10.19	0.92	8.58	99.08	8.64	9.34	1	2	8
3	34.74	25.58	5.32	1.26	1.95	97.82	10.58	1.54	0	1	4
4	20.92	2.67	1.76	36.15	41.59	61.67	52.17	1.15	1	1	5
5	20.92	2.67	1.76	24.71	25.34	61.67	52.17	1.03	0	0	1
6	26.24	0.97	0.49	13.17	13.10	36.96	77.52	0.99	1	0	2
7	38.11	0.61	0.33	2.35	1.09	23.79	90.62	0.46	2	0	3
8	34.87	0.63	0.33	9.43	3.66	21.44	91.70	0.39	2	1	6
9	19.13	0.61	0.36	12.01	4.63	12.01	95.37	0.39	2	2	9

Figure 13: Epistatic interaction of a univariately "strong" SNP with a SNP showing no effect whatsoever, in CD data. Epistatic interactions of this pair of SNPs showing both protective and contributory effects, on WTCCC CD data. Note this is a pair of proximal probes on the array, spaced $\approx$ 60K bp in the genome.

5 Additional Manhattan plots

Here we show plots analogous to Figure 1 in the main paper, but for the remaining 6 diseases (other than Rheumatoid Arthritis).

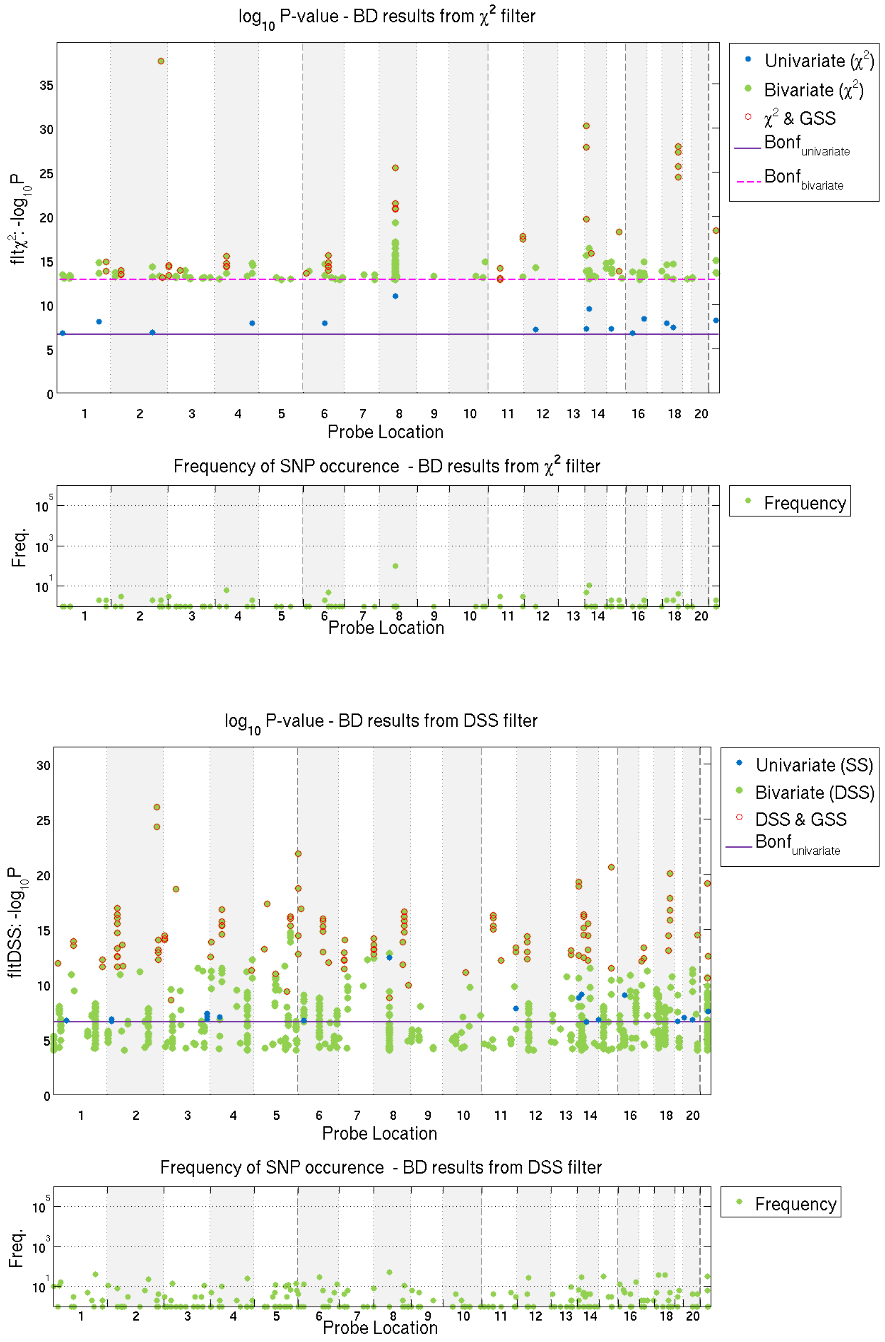

Figure 14: Manhattan plots for filtered pairs in for Bipolar Disorder (BD) data. This is figure analogous to Fig.1 in the paper for Rheumatoid Arthritis. In this figure, blue dot marks univariately significant SNP; green dot - a SNP in a significant pair, either χ^2 (top) or $\mathrm{flt}_{\mathrm{DSS}}$ (bottom); red circle - a SNP in a pair additionally significant according to $\mathrm{flt}_{\mathrm{GSS}}$. For the DSS plot, the univariate strength is marked by $\mathrm{flt}_{\mathrm{SS}}$ and is on the same scale as the $\mathrm{flt}_{\mathrm{DSS}}$ for the pair.

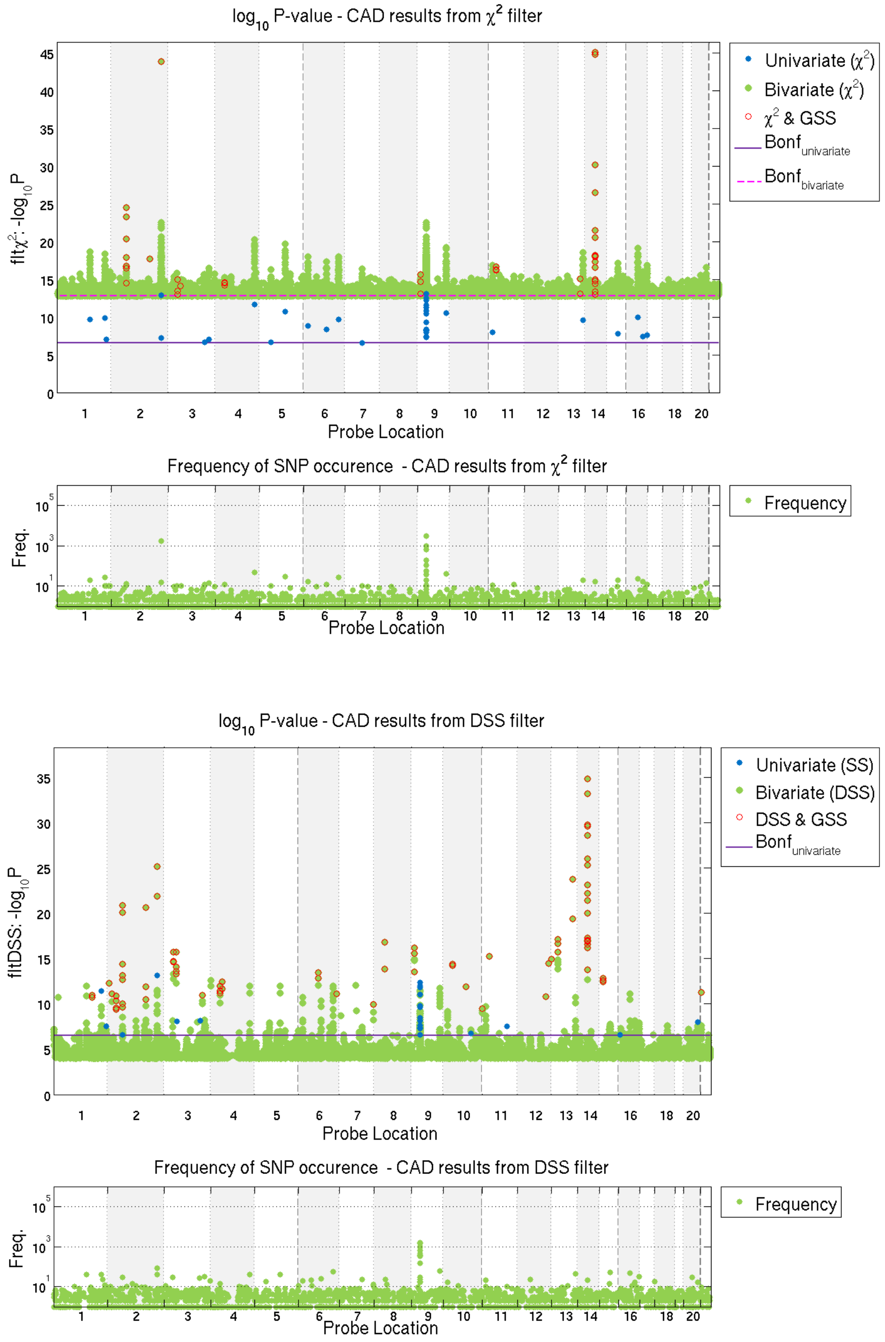

Figure 15: Manhattan plots for filtered pairs in for Coronary Artery Disease (CAD) data. This is figure analogous to Fig.1 in the paper for Rheumatoid Arthritis. In this figure, blue dot marks univariately significant SNP; green dot - a SNP in a significant pair, either χ^2 (top) or flt_{DSS} (bottom); red circle - a SNP in a pair additionally significant according to flt_{GSS}. For the DSS plot, the univariate strength is marked by flt_{SS} and is on the same scale as the flt_{DSS} for the pair.

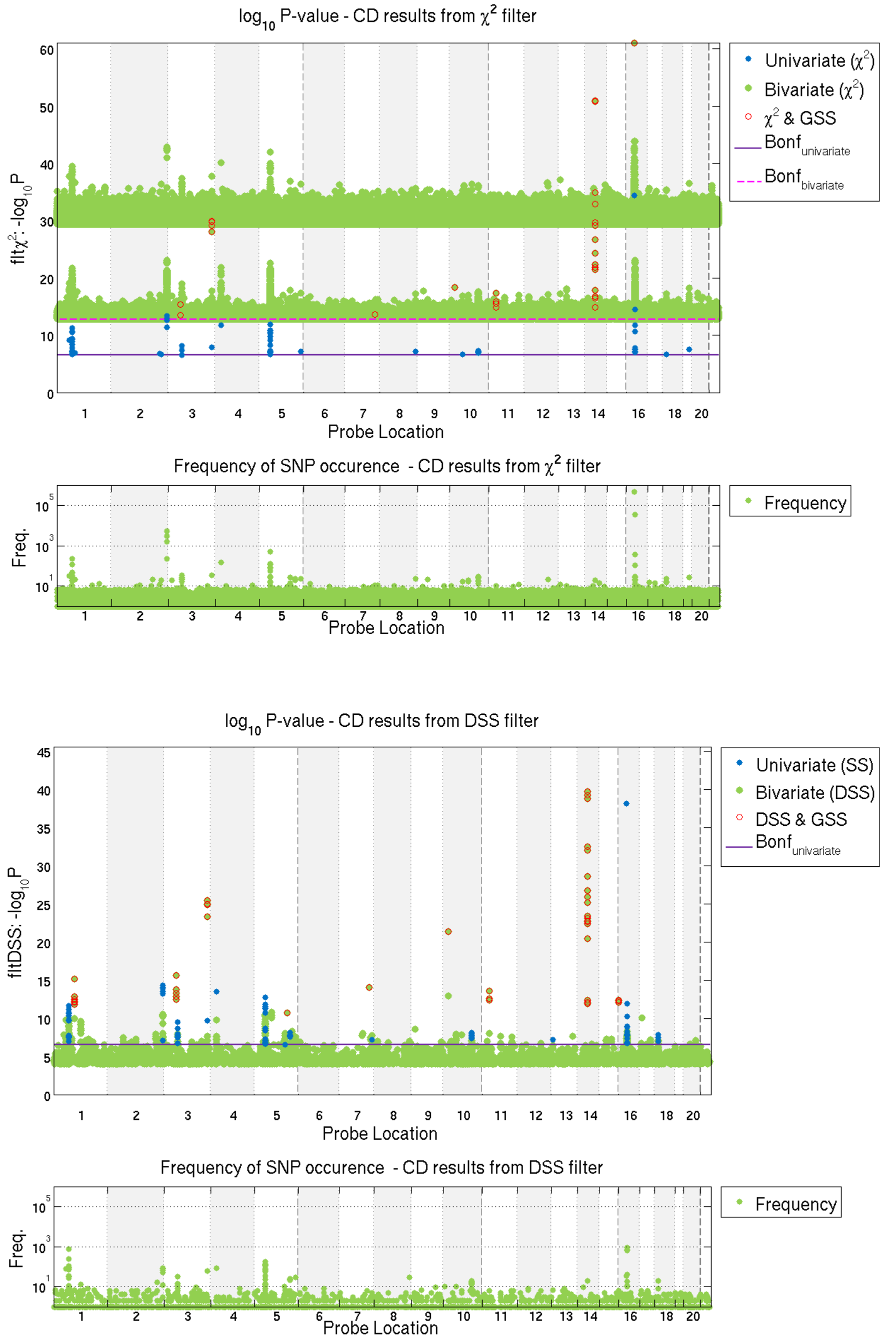

Figure 16: Manhattan plots for filtered pairs in for Crohn's Disease (CD) data. This is figure analogous to Fig.1 in the paper for Rheumatoid Arthritis. In this figure, blue dot marks univariately significant SNP; green dot - a SNP in a significant pair, either χ^2 (top) or flt_{DSS} (bottom); red circle - a SNP in a pair additionally significant according to flt_{GSS}. For the DSS plot, the univariate strength is marked by flt_{SS} and is on the same scale as the flt_{DSS} for the pair.

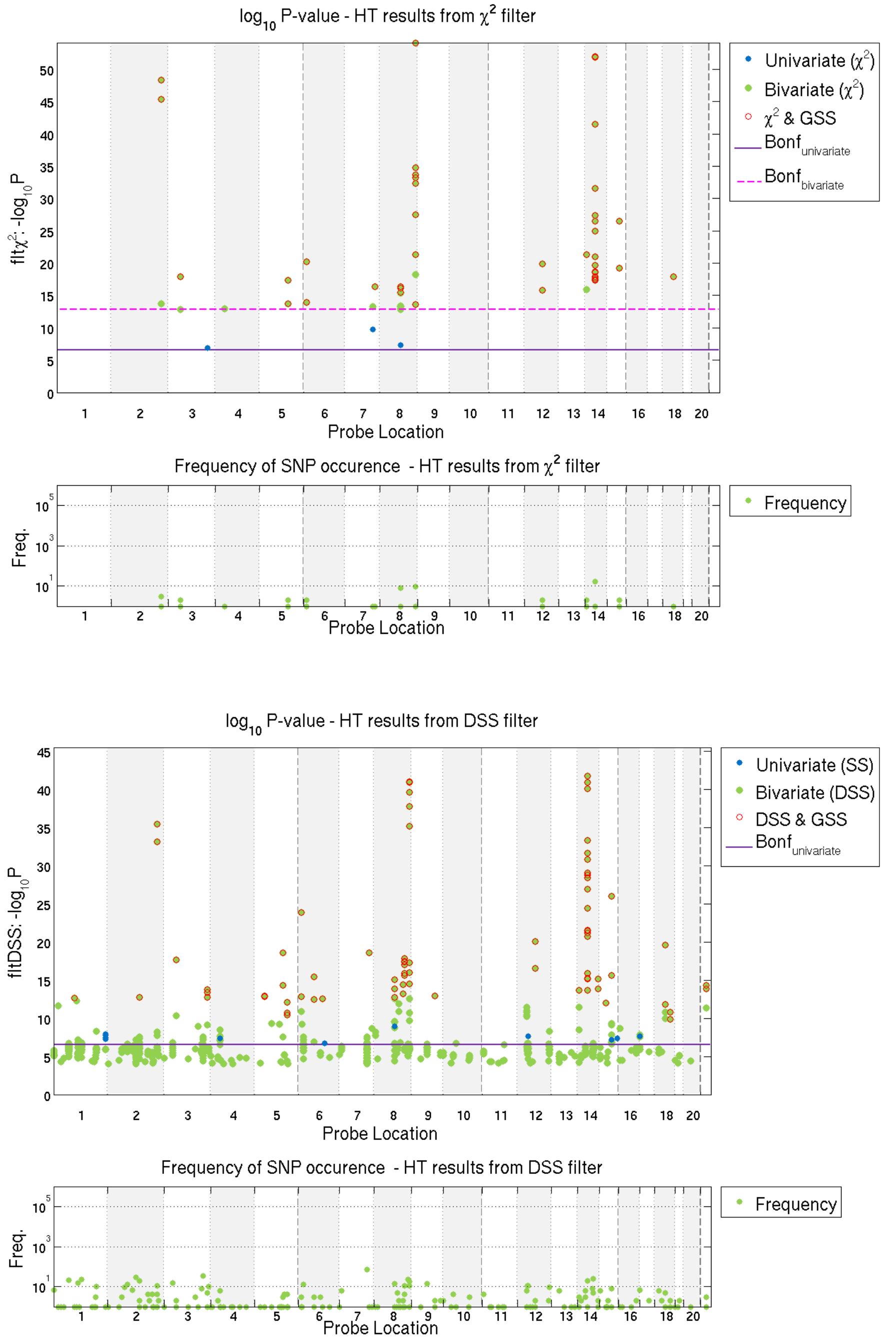

Figure 17: Manhattan plots for filtered pairs in for Hypertension (HT) data. This is figure analogous to Fig.1 in the paper for Rheumatoid Arthritis. In this figure, blue dot marks univariately significant SNP; green dot - a SNP in a significant pair, either χ^2 (top) or $\mathrm{flt}_{\mathrm{DSS}}$ (bottom); red circle - a SNP in a pair additionally significant according to $\mathrm{flt}_{\mathrm{GSS}}$. For the DSS plot, the univariate strength is marked by $\mathrm{flt}_{\mathrm{SS}}$ and is on the same scale as the $\mathrm{flt}_{\mathrm{DSS}}$ for the pair.

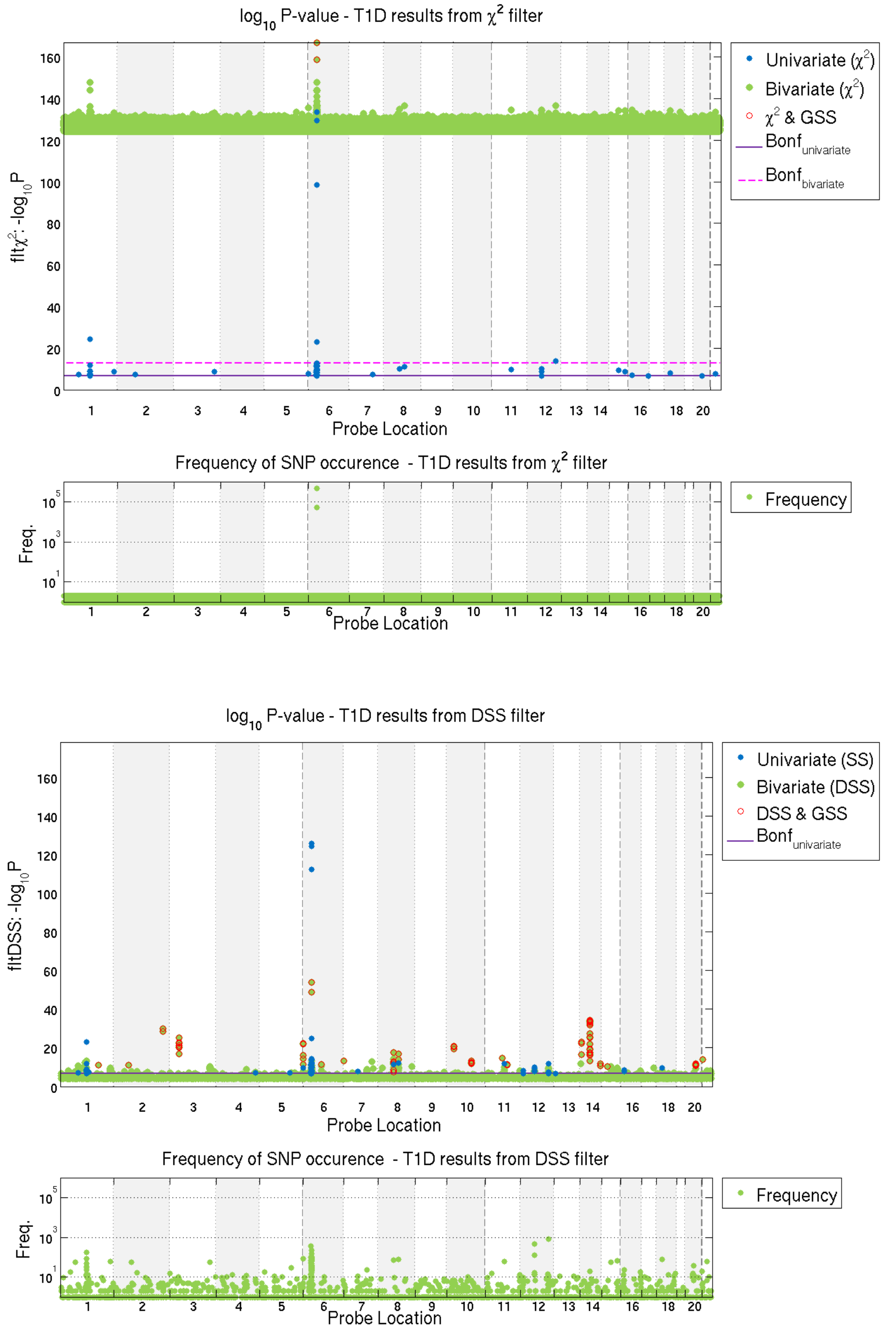

Figure 18: Manhattan plots for filtered pairs in for Type 1 Diabetes (T1D) data. This is figure analogous to Fig.1 in the paper for Rheumatoid Arthritis. In this figure, blue dot marks univariately significant SNP; green dot - a SNP in a significant pair, either χ^2 (top) or flt_{DSS} (bottom); red circle - a SNP in a pair additionally significant according to flt_{GSS}. For the DSS plot, the univariate strength is marked by flt_{SS} and is on the same scale as the flt_{DSS} for the pair. Note that the "extreme" p-values seen here for χ^2 are due to the strong associations coming from the HLA region which have been previously reported [11]. Similar values can be seen when using the SS filter which measures association, but are greatly reduced when using flt_{DSS} or $fltGSS$ which search for "epistatic effects".

Manhattan plots for filtered pairs in for Bipolar Disorder (BD) data. This is figure analogous to Fig.1 in the paper for Rheumatoid Arthritis. In this figure, blue dot marks univariately significant SNP; green dot - a SNP in a significant pair, either χ^2 (top) or flt_{DSS} (bottom); red circle - a SNP in a pair additionally significant according to flt_{GSS}. For the DSS plot, the univariate strength is marked by flt_{SS} and is on the same scale as the flt_{DSS} for the pair.

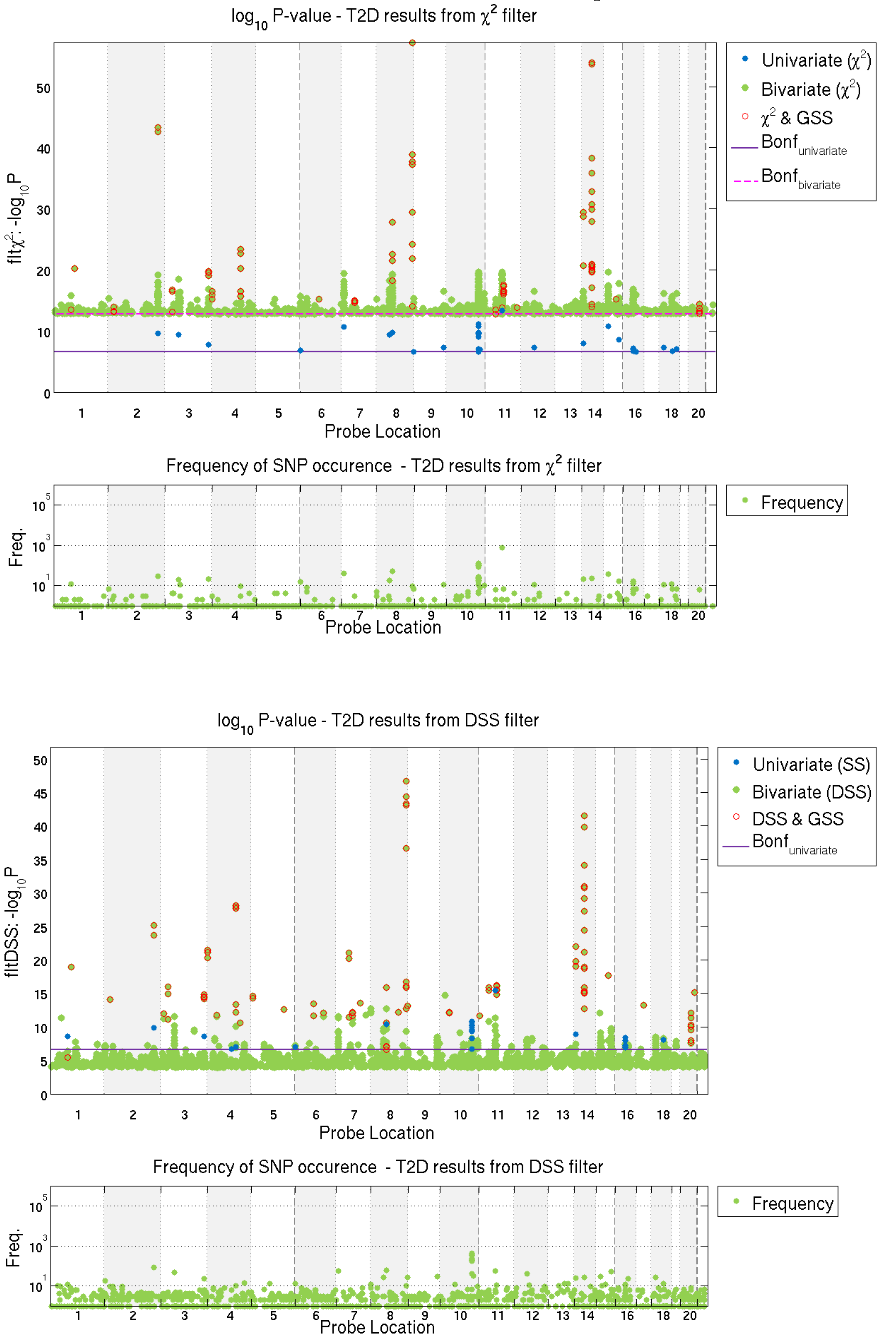

Figure 19: Manhattan plots for filtered pairs in for Type 2 Diabetes (T2D) data. This is figure analogous to Fig.1 in the paper for Rheumatoid Arthritis. In this figure, blue dot marks univariately significant SNP; green dot - a SNP in a significant pair, either χ^2 (top) or flt_{DSS} (bottom); red circle - a SNP in a pair additionally significant according to flt_{GSS}. For the DSS plot, the univariate strength is marked by flt_{SS} and is on the same Scale as the flt_{DSS} for the pair.

6 Additional Plots

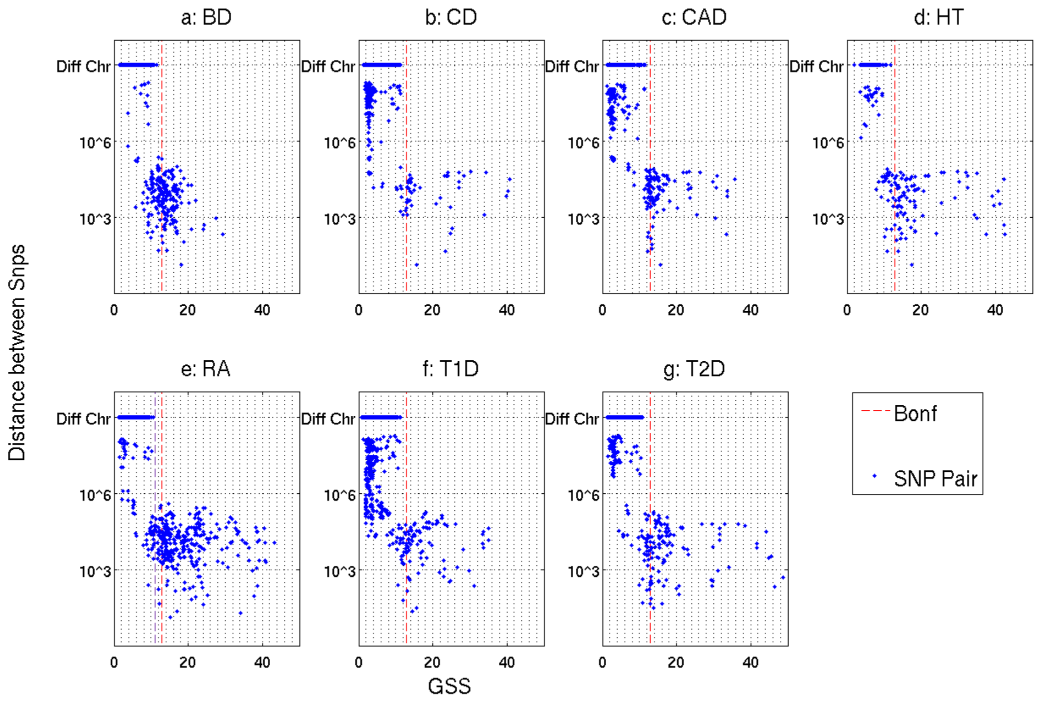

Figure 20: This figure plots genomic distances (base pairs) against flt_{GSS} scores for the top $500K$ pairs detected by flt_{DSS} for seven WTCCC datasets. For pairs on different chromosomes we have used a default distance of 10^9 bp. In each dataset we observe an abundance of such pairs, but predominantly with flt_{GSS} scores below Bonferroni threshold, i.e. $< \log_{10}\binom{n_{snp}}{2} \approx 11$ marked by red vertical lines. This scarcity of strong inter-chromosomal interaction signals in these datasets is in agreement with previous studies of WTCCC datasets.

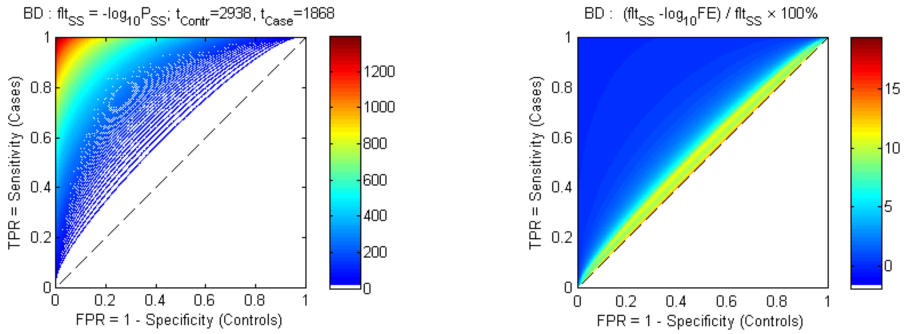

Figure 21: Comparison between Fisher exact and SS tests on Bipolar Disorder data. The left panel shows distribution of $\text{flt}_{\text{ss}} = -\log_{10} P_{\text{ss}}$ values. The contour lines are drawn every decade. The right panel shows the relative difference between $-\log_{10}$ p-values for Fisher Exact as a percentage of the flt_{ss} value. We observe that in the critical regions of interest, i.e. above the Bonferroni threshold, $\approx 10^{11}$, the difference is below 5%. Although this amounts to a huge factor, for practical scanning with those filters, where logarithms are used rather than the p-values, we expect that the performance of both filters will be virtually identical.

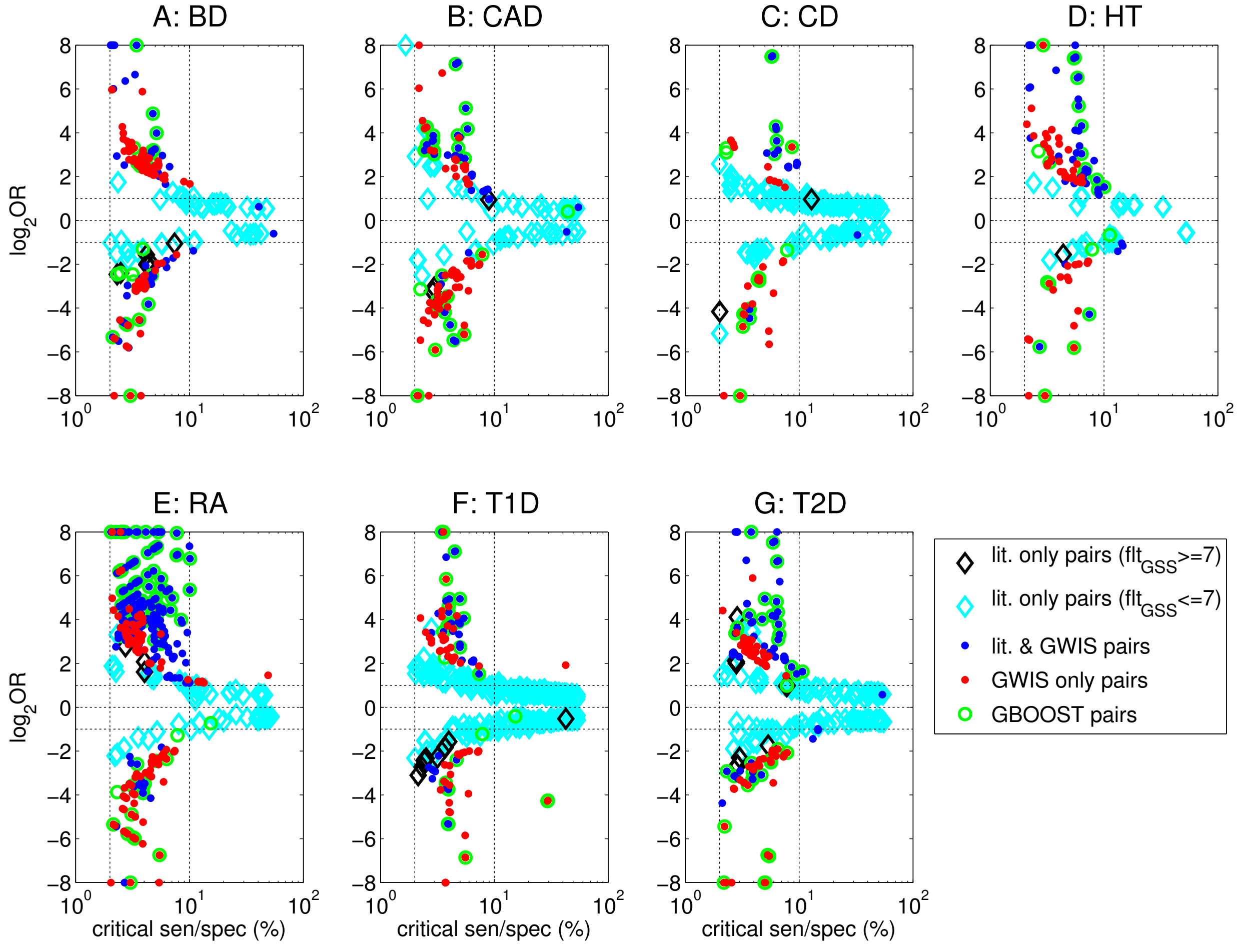

Figure 22: Comparison of GWIS results to literature and GBOOST for relaxation of epistasis criterion to $\mathrm{flt}_{\mathrm{GSS}} \geq 7$ (from $\mathrm{flt}_{\mathrm{GSS}} \geq 11$ used in Figure 2 of the paper). We plot odds ratio (OR) vs. "critical sens/spec", i.e. sensitivity for contributing genotypes ($\log_2 \mathrm{OR} > 0$) or vs. specificity for protective genotypes ($\log_2 \mathrm{OR} < 0$). We show pairs from seven WTCCC datasets reported by GWIS or in previous literature. Results from GBOOSThave also been indicated. Each pair is represented by a point whose style indicates the methods it was reported by. Here, GWIS results have been selected such that $\mathrm{flt}_{\mathrm{GSS}} > 7$. The left most vertical dotted line marks the formal minimum requirement of critical sens/spec $\geq 2\%$, while such horizontal lines are for $log_2\mathrm{OR} = \pm 1$ corresponding to $\mathrm{OR} = 2$ or $\mathrm{OR} = 1/2$, respectively. Note that for simplicity we did not relaxed DSS pre-filter, which could increases dramatically number of GWIS pairs.

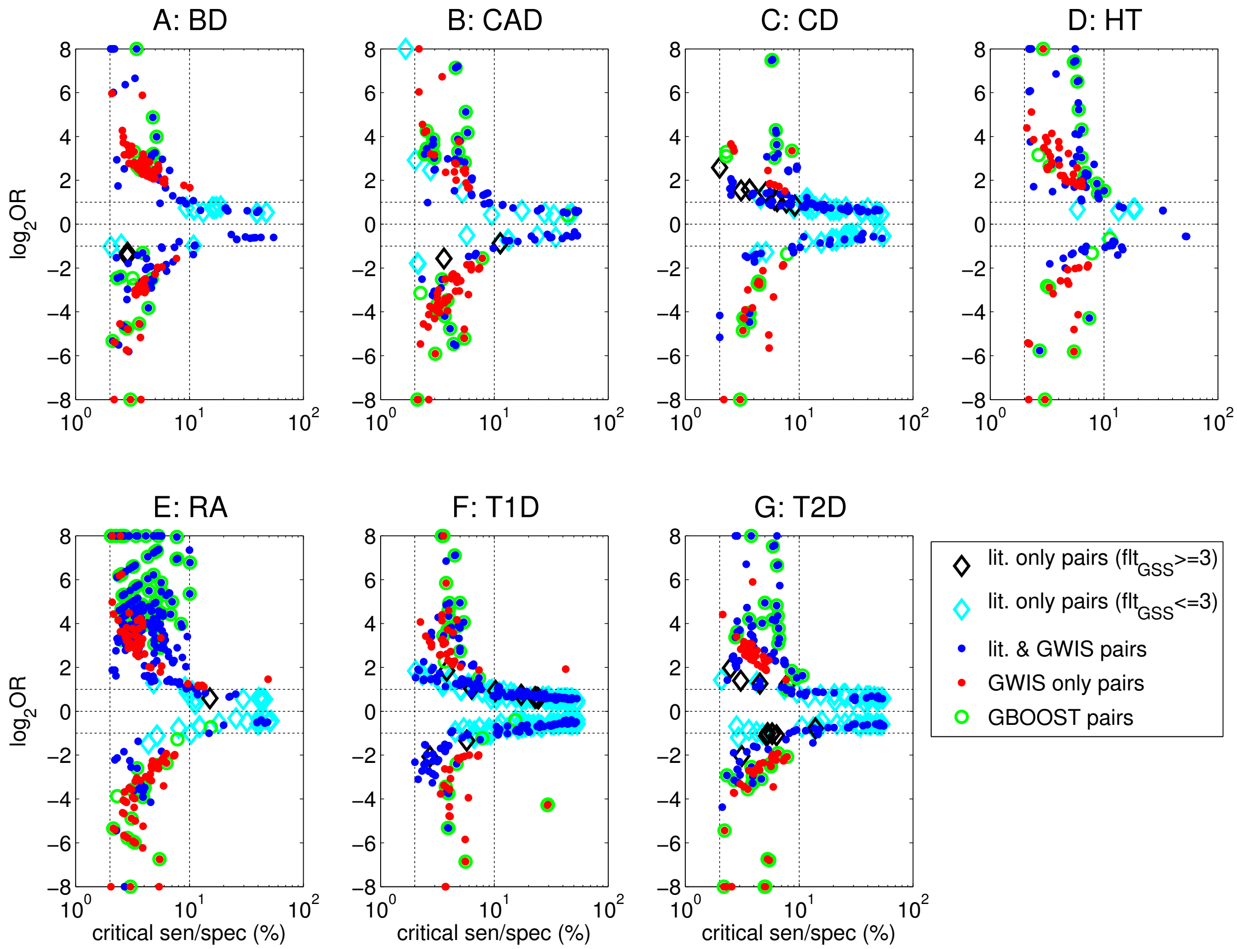

Figure 23: Comparison of GWIS results to literature and GBOOST for relaxation of epistasis criterion to $\mathrm{flt}_{\mathrm{GSS}} \geq 3$ (from $\mathrm{flt}_{\mathrm{GSS}} \geq 11$ used in Figure 2 of the paper). This figure is analogous to the previous one i.e. Supplementary Figure 22.

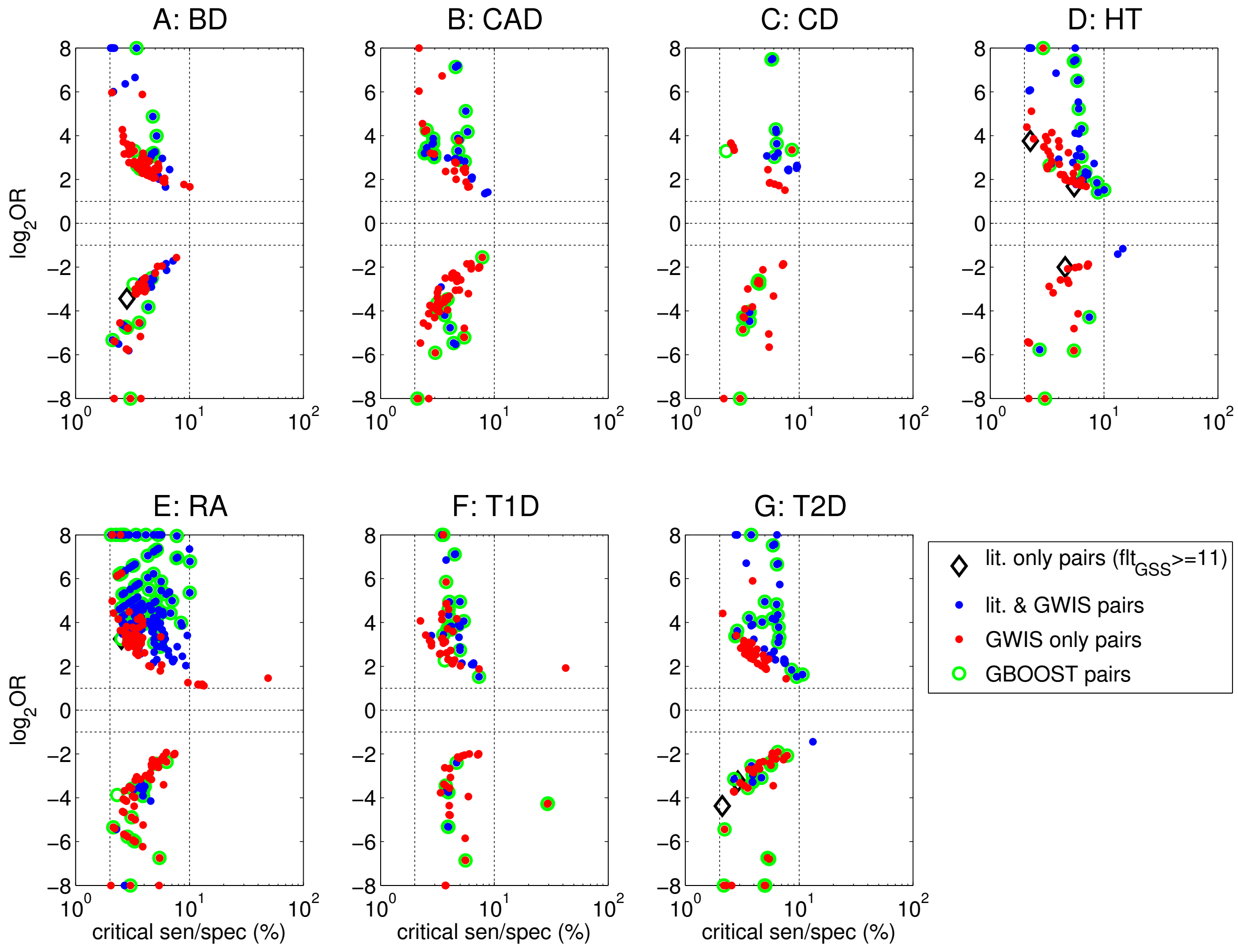

Figure 24: Plot of overlap between SNP pairs found by GWIS using the DSS filter and previous studies after strict GSS filtering ($\text{flt}_{\text{GSS}} \geq 11$), see Columns 3 and 4 of Table 4, the main paper. As previously we show pairs reported by GWIS, previous literature or by GBOOST. For each pair, we plot odds ratio (OR) vs. "critical sens/spec". Here, the literature results have been selected such that $\text{flt}_{\text{GSS}} > 11$ to clearly demonstrate which literature pairs have not been selected using the primary stage DSS filter. The left most vertical dotted line marks the formal minimum requirement of critical sens/spec $\geq 2\%$, while such horizontal lines are for $log_2\text{OR} = \pm 1$ corresponding to $\text{OR} = 2$ or $\text{OR} = 1/2$, respectively.

7 Pairs detected by GWIS

In this section, we list the pairs detected by GWIS for each of the WTCCC datasets. Pairs are sorted by the strength of association according to our GSS filter. For each pair, we show a number of characteristics:

- ID - Rank of the SNP pair in the list when sorted by GSS.
- rs - Rs identifier.
- chr - Chromosome.
- pos - Position relative to chromosome (in base pairs).
- χ^2 - $-log_{10}$ P-value according to χ^2 test for individual SNP.
- flt_{GSS} - $-log_{10}$ P-value according to GSS filter for pair of SNPs.
- Prev. Lit. - denotes previous work that has reported this SNP pair.

The previous literature results have been compiled from [10, 12–14] and also includes the results of running the GBOOST software [6] over the WTCCC datasets using default parameters.

We should emphasise that these pairs need to be screened for errors, especially for quality control of genotype calls, before any biological conclusions can be drawn.

Here we treat the list of pairs shown below as formal validation of our novel filter techniques for detection of statistically significant associations. We have used genotype calls as provided by WTCCC. In particular, no genotype calls were marked as missing. While the WTCCC list of SNPs to exclude for quality control reasons was used, some evidence exists that genotyping errors remain [10]. The data unmodified allows others to compare against our results, presented in the tables below.

7.1 Bipolar Disorder Results

ID	rs_1	rs_2	chr_1	bp_1	chr_2	bp_2	χ_1^2	χ_2^2	flt_{GSS}	source
1	rs7844299	rs11984645	8	50430790	8	55069305	0.9	50.4	14.4	[10], [6]
2	rs6983650	rs11984645	8	50406226	8	55069305	0.4	50.4	14.4	[10], [6]
3	rs10099080	rs11984645	8	50357376	8	55069305	0.8	50.4	13.7	[10], [6]
4	rs6473901	rs11984645	8	50386264	8	55069305	1.4	50.4	13.5	[10], [6]
5	rs6473903	rs11984645	8	50388147	8	55069305	0.0	50.4	12.6	[10]
6	rs4654792	rs909812	1	22600672	1	22603366	3.2	2.5	12.7	
7	rs199698	rs41515647	1	75636696	1	75647488	0.8	19.7	11.9	
8	rs625045	rs668860	1	85434613	1	85436462	2.1	4.5	16.5	
9	rs668860	rs10873672	1	85436462	1	85450726	4.5	2.3	16.6	
10	rs668860	rs6691970	1	85436462	1	85450920	4.5	2.3	16.3	
11	rs10922726	rs1782127	1	90265279	1	90280342	3.6	17.5	13.2	

12	rs7417737	rs3811480	1	230468063	1	230469202	0.5	8.3	15.0	
13	rs7414802	rs3811480	1	230468275	1	230469202	0.5	8.3	14.3	
14	rs7597849	rs1595734	2	35744997	2	35813424	1.1	5.1	17.8	
15	rs1371427	rs1595734	2	35779358	2	35813424	1.3	5.1	19.0	
16	rs2719093	rs1595734	2	35795100	2	35813424	3.7	5.1	15.4	
17	rs2372008	rs1595734	2	35801114	2	35813424	3.0	5.1	14.2	
18	rs2372009	rs1595734	2	35802886	2	35813424	0.7	5.1	18.4	
19	rs1595734	rs1439684	2	35813424	2	35826687	5.1	1.1	16.7	
20	rs1595734	rs2043914	2	35813424	2	35831008	5.1	1.3	14.5	
21	rs1595734	rs13011599	2	35813424	2	35848903	5.1	0.9	14.7	
22	rs13028177	rs6543990	2	36236450	2	36240074	1.8	0.5	17.5	
23	rs7570865	rs6544738	2	44305057	2	44305240	9.8	1.0	13.8	
24	rs350747	rs350753	2	52868656	2	52873788	6.1	1.3	16.2	
25	rs17046061	rs17046067	2	54596679	2	54600145	0.6	11.4	14.2	[10]
26	rs16849921	rs10197379	2	214061022	2	205912603	18.6	0.0	30.4	[10], [6]
27	rs16849921	rs12694298	2	214061022	2	205913268	18.6	0.2	28.6	[10], [6]
28	rs1983218	rs2738290	2	217336645	2	217352253	1.1	9.4	14.8	
29	rs2280043	rs2738290	2	217342396	2	217352253	0.9	9.4	15.5	
30	rs1110998	rs2738290	2	217343952	2	217352253	0.7	9.4	15.7	
31	rs2738287	rs2738290	2	217349836	2	217352253	0.8	9.4	16.6	
32	rs17193526	rs17786145	3	1500657	3	1445217	0.3	14.2	16.7	
33	rs17786145	rs17039822	3	1445217	3	1453429	14.2	0.1	15.9	
34	rs17786145	rs17786151	3	1445217	3	1455381	14.2	0.5	16.0	
35	rs939298	rs1520050	3	20477076	3	20500104	2.1	13.3	13.4	
36	rs9859908	rs1520050	3	20489086	3	20500104	2.8	13.3	12.7	
37	rs6809441	rs33916626	3	41494605	3	41539388	2.7	0.5	19.2	[6]
38	rs506996	rs382441	4	2000258	4	2009095	8.1	1.6	14.7	
39	rs4600860	rs4688938	4	5508488	4	5516030	0.7	10.9	13.0	[10]
40	rs4688938	rs4586871	4	5516030	4	5516378	10.9	0.7	14.8	[10]
41	rs4688938	rs6446371	4	5516030	4	5526209	10.9	0.0	11.4	
42	rs4688938	rs2267654	4	5516030	4	5527249	10.9	0.1	12.1	[10]
43	rs4688938	rs16837389	4	5516030	4	5536310	10.9	0.6	12.3	[10]
44	rs4695062	rs10032741	4	44909419	4	44960444	3.2	15.7	11.3	[6]
45	rs1948584	rs10032741	4	44909853	4	44960444	1.8	15.7	14.3	
46	rs7690764	rs10032741	4	44924382	4	44960444	0.9	15.7	13.3	
47	rs7690944	rs10032741	4	44924464	4	44960444	1.7	15.7	11.2	
48	rs1390911	rs10032741	4	44253487	4	44960444	2.9	15.7	13.8	
49	rs1845945	rs10032741	4	44933047	4	44960444	0.2	15.7	14.0	
50	rs9998147	rs10032741	4	44934205	4	44960444	0.3	15.7	13.9	
51	rs10032741	rs1908806	4	44960444	4	44964953	15.7	0.5	15.1	
52	rs2545308	rs2613097	4	181637915	4	181639323	6.2	4.8	13.7	
53	rs34691926	rs6945822	4	183829027	7	129698576	7.6	3.9	11.8	
54	rs34691926	rs11624794	4	183829027	14	24260146	7.6	11.2	11.2	
55	rs7735940	rs12515142	5	36423931	5	36426411	0.6	0.9	14.2	[10], [6]
56	rs2935260	rs2992406	5	54444880	5	54444983	0.0	6.3	19.1	[13], [10]
57	rs10060331	rs12188163	5	93020050	5	93250608	6.2	13.7	12.9	[6]
58	rs13154650	rs12188163	5	93052205	5	93250608	4.5	13.7	13.2	
59	rs1470150	rs12188163	5	93138654	5	93250608	7.2	13.7	12.4	
60	rs13155452	rs12188163	5	93164449	5	93250608	3.3	13.7	13.3	
61	rs17083377	rs12188163	5	93164870	5	93250608	6.3	13.7	12.5	
62	rs10075217	rs12188163	5	93176774	5	93250608	3.3	13.7	13.0	
63	rs6874766	rs12188163	5	93213807	5	93250608	3.1	13.7	13.0	
64	rs7715562	rs12188163	5	93222034	5	93250608	3.5	13.7	12.9	
65	rs896729	rs12188163	5	93222178	5	93250608	6.3	13.7	12.3	
66	rs17314825	rs12188163	5	93234937	5	93250608	2.8	13.7	13.3	

67	rs10214278	rs12188163	5	93237679	5	93250608	2.8	13.7	13.3	
68	rs12188163	rs10053405	5	93250608	5	93275403	13.7	1.9	13.0	
69	rs11744199	rs3749820	5	132527098	5	132533063	1.2	7.1	11.9	
70	rs17287085	rs7700706	5	141284815	5	141294362	2.7	17.9	13.4	
71	rs7700706	rs6580205	5	141294362	5	141294691	17.9	4.1	13.4	
72	rs7700706	rs758462	5	141294362	5	141297178	17.9	5.0	12.0	
73	rs12515561	rs1552835	5	152955611	5	152959769	1.1	4.8	13.0	[10], [6]
74	rs12515563	rs1552835	5	152955632	5	152959769	2.5	4.8	13.2	
75	rs12515520	rs1552835	5	152955663	5	152959769	1.2	4.8	12.7	[10], [6]
76	rs1552837	rs1552835	5	152959669	5	152959769	2.5	4.8	14.0	
77	rs1552835	rs17519558	5	152959769	5	152960319	4.8	1.4	14.7	[13], [10]
78	rs1552835	rs17519656	5	152959769	5	152960528	4.8	3.2	13.4	
79	rs1552835	rs17591636	5	152959769	5	152960615	4.8	2.1	14.5	
80	rs2438077	rs2438083	6	1272236	6	1277371	3.4	0.1	14.6	
81	rs2496292	rs2438083	6	1274617	6	1277371	6.8	0.1	16.8	
82	rs2438083	rs977674	6	1277371	6	1277702	0.1	7.5	21.1	
83	rs2438083	rs977673	6	1277371	6	1277715	0.1	7.7	24.3	
84	rs9357438	rs9357440	6	9492158	6	9492393	1.1	11.3	18.0	[10]
85	rs1886330	rs365237	6	18162229	6	18186697	2.2	23.2	11.2	
86	rs365237	rs214614	6	18186697	6	18201306	23.2	4.3	11.3	
87	rs365237	rs214610	6	18186697	6	18203094	23.2	4.1	11.3	
88	rs365237	rs214599	6	18186697	6	18207443	23.2	2.4	11.6	
89	rs10499047	rs9320174	6	106983128	6	106985408	4.8	10.0	16.4	
90	rs9486383	rs9320174	6	106983359	6	106985408	4.5	10.0	15.7	
91	rs9480682	rs9320174	6	106983406	6	106985408	4.1	10.0	16.2	
92	rs13218960	rs9320174	6	106983465	6	106985408	4.6	10.0	16.2	[13]
93	rs9320173	rs9320174	6	106984669	6	106985408	4.5	10.0	16.5	
94	rs9320174	rs783397	6	106985408	6	106987161	10.0	1.5	14.2	
95	rs1729549	rs1190806	6	129087423	6	129106943	4.6	0.5	14.2	
96	rs985307	rs985882	7	19573763	7	19575505	4.1	0.5	15.7	[10]
97	rs985306	rs985882	7	19574066	7	19575505	4.9	0.5	15.1	[10]
98	rs2192481	rs985882	7	19574677	7	19575505	7.2	0.5	14.7	[10]
99	rs985882	rs985881	7	19575505	7	19575555	0.5	4.4	14.9	[10]
100	rs7781714	rs6949019	7	23593479	7	23594050	1.2	6.6	16.9	[10]
101	rs10253608	rs10266006	7	158469060	7	158474325	2.3	2.0	14.1	
102	rs12113120	rs10266006	7	158470080	7	158474325	2.2	2.0	14.5	
103	rs10949739	rs10266006	7	158472082	7	158474325	2.5	2.0	14.3	
104	rs4909259	rs10266006	7	158473900	7	158474325	2.2	2.0	14.5	
105	rs10266006	rs10237585	7	158474325	7	158474373	2.0	2.0	13.0	
106	rs10266006	rs3793181	7	158474325	7	158481827	2.0	3.0	14.4	[13]
107	rs10266006	rs6459895	7	158474325	7	158482870	2.0	2.9	13.9	
108	rs10266006	rs12698265	7	158474325	7	158489297	2.0	2.2	14.5	
109	rs16919784	rs11984645	8	55063538	8	55069305	3.0	50.4	13.1	[10]
110	rs11984645	rs4737503	8	55069305	8	55071319	50.4	2.7	15.3	[10], [6]
111	rs7002915	rs4437686	8	114310388	8	114357413	0.9	2.9	13.5	
112	rs7012271	rs4437686	8	114317672	8	114357413	0.8	2.9	15.7	
113	rs2447183	rs2469997	8	120352978	8	120353267	0.5	1.4	18.1	[10]
114	rs2469996	rs2469997	8	120353011	8	120353267	0.2	1.4	17.7	[10]
115	rs2469997	rs6469823	8	120353267	8	120353984	1.4	0.3	17.5	[10]
116	rs2469997	rs2447179	8	120353267	8	120355775	1.4	0.4	18.1	[10]
117	rs2469997	rs2447178	8	120353267	8	120356188	1.4	0.3	17.9	[10]
118	rs2469997	rs2470002	8	120353267	8	120357424	1.4	0.2	17.7	[10]
119	rs2469997	rs2470025	8	120353267	8	120364727	1.4	0.3	17.9	[10]
120	rs2469997	rs2447169	8	120353267	8	120365063	1.4	0.3	18.0	[10]
121	rs2469997	rs2470026	8	120353267	8	120365112	1.4	0.2	16.8	[10]

122	rs2469997	rs2447168	8	120353267	8	120365613	1.4	0.3	18.5	[10]
123	rs2469997	rs2470040	8	120353267	8	120385452	1.4	0.4	16.7	[10]
124	rs7013124	rs7014384	8	130232771	8	130236557	13.8	0.3	13.1	
125	rs17459521	rs892542	10	80496476	10	80498057	3.2	0.7	13.4	[10], [6]
126	rs12805895	rs1461902	11	37707868	11	37708266	4.7	0.7	17.5	
127	rs12805895	rs7929645	11	37707868	11	37708514	4.7	0.7	17.8	
128	rs12805895	rs4756413	11	37707868	11	37708626	4.7	0.3	16.6	
129	rs12805895	rs1381428	11	37707868	11	37708653	4.7	1.2	17.0	
130	rs11237746	rs11237747	11	75175059	11	75180559	8.7	0.2	14.7	
131	rs1102478	rs858719	11	130089380	11	130160456	1.3	27.9	16.6	
132	rs7943478	rs858719	11	130089402	11	130160456	0.4	27.9	16.6	
133	rs2892410	rs11168985	12	38990282	12	39045983	0.8	2.3	14.5	
134	rs11168985	rs826886	12	39045983	12	39095797	2.3	1.6	16.7	
135	rs11168985	rs826838	12	39045983	12	39106731	2.3	1.8	15.0	
136	rs11168985	rs1164971	12	39045983	12	39110284	2.3	1.6	16.1	
137	rs869877	rs869878	13	75282465	13	75282895	8.5	1.5	12.6	
138	rs9561326	rs9561329	13	94007107	13	94011169	5.1	8.2	14.2	
139	rs9561329	rs9561336	13	94011169	13	94034140	8.2	7.1	13.9	
140	rs11624794	rs1958305	14	24260146	14	24273124	11.2	33.4	18.5	[10], [6]
141	rs1958305	rs17184408	14	24273124	14	24282020	33.4	0.5	17.9	[10], [6]
142	rs1958305	rs12100601	14	24273124	14	24284173	33.4	1.9	17.4	
143	rs1439170	rs7152370	14	46638877	14	46735956	1.4	2.8	18.0	[10]
144	rs858870	rs7152370	14	46666812	14	46735956	6.1	2.8	14.8	
145	rs17737767	rs7152370	14	46718257	14	46735956	1.4	2.8	17.8	[10]
146	rs7152370	rs10483596	14	46735956	14	46740909	2.8	5.8	17.0	[10]
147	rs7152370	rs8017858	14	46735956	14	46741726	2.8	1.3	16.8	[10]
148	rs4899113	rs12323479	14	63916159	14	63924082	1.0	1.2	16.4	
149	rs10152067	rs12323479	14	63916963	14	63924082	1.7	1.2	15.1	
150	rs12323479	rs8008684	14	63924082	14	63944791	1.2	1.1	17.4	[6]
151	rs12323479	rs17101239	14	63924082	14	63949994	1.2	1.6	14.7	
152	rs6574039	rs17105918	14	72491015	14	72494808	2.3	16.0	11.2	
153	rs17105918	rs4902976	14	72494808	14	72505876	16.0	1.7	13.2	[10]
154	rs746655	rs921535	15	74107677	15	74111343	4.5	9.6	21.3	[10]
155	rs921535	rs999742	15	74111343	15	74115517	9.6	14.8	13.9	[10]
156	rs12593542	rs7166105	15	75282147	15	75343942	7.6	1.5	11.2	
157	rs17139600	rs17139608	16	6279094	16	6281175	1.2	11.2	12.5	
158	rs886889	rs10521202	17	12804201	17	12814564	1.2	10.0	14.7	[10]
159	rs10521202	rs5017214	17	12814564	17	12817783	10.0	0.8	12.5	[10]
160	rs12941700	rs9906443	17	30185022	17	30185565	0.9	1.2	15.1	[10]
161	rs12941700	rs9899093	17	30185022	17	30191954	0.9	1.8	14.4	
162	rs8085631	rs7233258	18	57654043	18	57655248	2.1	2.5	14.9	
163	rs17066458	rs7233258	18	57654067	18	57655248	1.2	2.5	16.2	
164	rs1944328	rs1944327	18	61838457	18	61838947	21.8	4.4	25.0	[10]
165	rs1944328	rs8091006	18	61838457	18	61842934	21.8	0.3	23.0	[10], [6]
166	rs1944328	rs9675798	18	61838457	18	61856967	21.8	1.0	21.9	[10], [6]
167	rs1944328	rs9676116	18	61838457	18	61857065	21.8	2.2	21.0	[10], [6]
168	rs16999569	rs12980129	19	22902710	19	22908911	4.5	23.4	11.7	
169	rs12980129	rs2194111	19	22908911	19	22922549	23.4	4.2	11.7	
170	rs6095722	rs6020395	20	33045515	20	33074440	0.0	9.4	11.6	
171	rs16986890	rs6020395	20	33061637	20	33074440	0.0	9.4	12.5	
172	rs6020395	rs6020846	20	33074440	20	33140988	9.4	0.0	12.2	
173	rs2011703	rs6014572	20	54556388	20	54574544	16.7	1.6	16.7	[6]
174	rs8130402	rs999789	21	40420409	21	40426451	2.7	16.3	18.6	[10]
175	rs2836860	rs999789	21	40423964	21	40426451	1.6	16.3	12.3	
176	rs4279007	rs999789	21	40424954	21	40426451	1.6	16.3	13.1	

177	rs999789	rs428424	21	40426451	21	40445319	16.3	1.1	14.4
178	rs999789	rs445593	21	40426451	21	40448382	16.3	1.0	14.4
179	rs2837630	rs2837632	21	41818543	21	41819281	0.2	3.5	14.6

Table 2: BD results

7.2 Coronary Artery Disease Results

ID	rs_1	rs_2	chr_1	bp_1	chr_2	bp_2	χ^2_1	χ^2_2	flt_{GSS}	source
1	rs4471699	rs11863150	16	30320307	16	30385503	4.0	0.8	32.6	[6]
2	rs2146340	rs1782127	1	90185106	1	90280342	4.8	17.5	12.1	
3	rs12135351	rs1782127	1	90254276	1	90280342	3.1	17.5	14.3	[6]
4	rs10922719	rs1782127	1	90254411	1	90280342	3.9	17.5	15.0	[6]
5	rs7538578	rs1782127	1	90257041	1	90280342	4.3	17.5	14.4	[6]
6	rs10922725	rs1782127	1	90265153	1	90280342	3.8	17.5	14.1	[6]
7	rs10922726	rs1782127	1	90265279	1	90280342	3.6	17.5	17.4	
8	rs6663717	rs1782127	1	90267116	1	90280342	3.6	17.5	14.7	[6]
9	rs13069584	rs6809441	3	41516619	3	41494605	0.9	2.7	14.9	[6]
10	rs6599155	rs6809441	3	41489604	3	41494605	2.0	2.7	13.4	
11	rs12054014	rs6809441	3	41490064	3	41494605	0.2	2.7	14.2	[6]
12	rs12054016	rs6809441	3	41490294	3	41494605	0.3	2.7	14.0	[6]
13	rs6809441	rs33916626	3	41494605	3	41539388	2.7	0.5	16.7	[6]
14	rs959880	rs2314349	3	183088613	3	183091098	5.2	7.8	25.7	[10]
15	rs2314349	rs906719	3	183091098	3	183091144	7.8	4.6	24.4	[10]
16	rs2314349	rs2314348	3	183091098	3	183091330	7.8	5.4	25.8	[10]
17	rs2314349	rs2089588	3	183091098	3	183091474	7.8	5.2	26.1	[10]
18	rs10065019	rs417769	5	71794569	5	71811478	2.5	7.1	12.2	
19	rs4703937	rs417769	5	71797851	5	71811478	2.6	7.1	12.5	
20	rs246571	rs417769	5	71805150	5	71811478	2.7	7.1	12.5	
21	rs246562	rs417769	5	71810246	5	71811478	1.9	7.1	12.9	
22	rs17287085	rs7700706	5	141284815	5	141294362	2.7	17.9	13.8	
23	rs7700706	rs758462	5	141294362	5	141297178	17.9	5.0	13.8	
24	rs11526287	rs2250603	7	135297419	7	135315708	0.2	2.7	15.3	
25	rs1535624	rs7028357	9	7120095	9	7132694	1.3	1.4	11.4	
26	rs17152197	rs17152205	10	12792541	10	12794336	1.0	0.4	13.3	
27	rs17152205	rs41380844	10	12794336	10	12797828	0.4	1.2	21.8	[10]
28	rs11027910	rs10219185	11	24458713	11	24504903	2.3	0.2	14.6	[10], [6]
29	rs10500978	rs10219185	11	24493480	11	24504903	2.4	0.2	15.7	[10], [6]
30	rs10500979	rs10219185	11	24493876	11	24504903	0.3	0.2	14.6	[10], [6]
31	rs10500980	rs10219185	11	24494465	11	24504903	1.2	0.2	14.7	[10], [6]
32	rs11849674	rs7154773	14	60688023	14	40912999	4.7	2.5	30.6	[10], [6]
33	rs10148587	rs7154773	14	60689160	14	40912999	4.2	2.5	28.0	[10]
34	rs188620	rs7154773	14	40853319	14	40912999	0.8	2.5	24.7	[10], [6]
35	rs10137732	rs7154773	14	60689209	14	40912999	4.4	2.5	34.4	[10], [6]
36	rs6573298	rs7154773	14	40869612	14	40912999	2.1	2.5	24.5	[10]
37	rs7145505	rs7154773	14	40869718	14	40912999	1.6	2.5	24.3	[10]
38	rs8019531	rs7154773	14	40871554	14	40912999	3.1	2.5	29.2	
39	rs11628587	rs7154773	14	40871574	14	40912999	1.8	2.5	25.1	[10]
40	rs11628628	rs7154773	14	40871689	14	40912999	2.0	2.5	24.8	[10]
41	rs8011227	rs7154773	14	40882151	14	40912999	1.9	2.5	41.7	[10]
42	rs7158657	rs7154773	14	40888940	14	40912999	2.9	2.5	27.7	
43	rs10142834	rs7154773	14	40898846	14	40912999	3.0	2.5	27.7	

44	rs17097262	rs7154773	14	40902242	14	40912999	1.0	2.5	41.3	[10], [6]
45	rs1887103	rs7154773	14	40907104	14	40912999	3.1	2.5	22.9	
46	rs7154773	rs8012816	14	40912999	14	40914246	2.5	1.1	33.9	[10]
47	rs7154773	rs10130695	14	40912999	14	40919869	2.5	1.3	40.9	[13], [10], [6]
48	rs7154773	rs1998225	14	40912999	14	40941373	2.5	1.9	14.5	
49	rs7154773	rs8004280	14	40912999	14	60780429	2.5	2.2	14.6	
50	rs7154773	rs1951117	14	40912999	14	60782214	2.5	2.0	14.8	
51	rs1625279	rs1742431	16	1901844	16	1905874	0.7	0.4	14.1	
52	rs1625393	rs1742431	16	1901893	16	1905874	0.8	0.4	14.0	
53	rs1657117	rs1742431	16	1903651	16	1905874	0.8	0.4	14.0	
54	rs7199384	rs1742431	16	1904587	16	1905874	0.6	0.4	14.1	
55	rs1742431	rs1657094	16	1905874	16	1920629	0.4	0.5	14.3	
56	rs1742431	rs2974856	16	1905874	16	1920757	0.4	0.5	14.1	
57	rs237348	rs7213121	17	12426753	17	12428464	0.5	7.9	11.9	

Table 3: CAD results

7.3 Chron's Disease Results

ID	rs_1	rs_2	chr_1	bp_1	chr_2	bp_2	χ_1^2	χ_2^2	flt_{GSS}	source
1	rs6531271	rs12472889	2	17139933	2	17161082	0.0	0.2	13.5	[10], [6]
2	rs3773090	rs9833015	3	29609837	3	29680632	0.1	1.9	13.1	[6]
3	rs12620194	rs12472889	2	17148004	2	17161082	0.3	0.2	12.6	[10], [6]
4	rs10495652	rs12472889	2	17139289	2	17161082	0.1	0.2	11.8	[10], [6]
5	rs4654792	rs909812	1	22600672	1	22603366	3.2	2.5	12.3	
6	rs6683655	rs12137523	1	188436602	1	188442049	2.3	1.5	14.9	
7	rs12137523	rs4396083	1	188442049	1	188448223	1.5	2.3	14.7	
8	rs10178286	rs10187740	2	3823821	2	3823965	1.5	0.4	13.1	
9	rs3954862	rs6432267	2	12304753	2	12318466	1.6	8.3	12.3	
10	rs6733006	rs6432267	2	12316070	2	12318466	2.6	8.3	13.2	[10]
11	rs3768666	rs6737027	2	29005870	2	29070805	1.4	2.4	14.7	
12	rs7601413	rs6737027	2	28774193	2	29070805	1.7	2.4	15.5	
13	rs7601934	rs6737027	2	29032673	2	29070805	1.8	2.4	16.0	
14	rs6547881	rs6737027	2	29032746	2	29070805	1.7	2.4	14.6	
15	rs4535080	rs10167057	2	52259604	2	52288297	0.5	0.3	15.0	[6]
16	rs13397279	rs10167057	2	52274871	2	52288297	0.7	0.3	14.0	
17	rs10167057	rs6723221	2	52288297	2	52034007	0.3	3.1	24.3	[10], [6]
18	rs10167057	rs4619644	2	52288297	2	52300414	0.3	0.8	12.3	
19	rs10167057	rs1806739	2	52288297	2	52301200	0.3	1.1	16.2	
20	rs10167057	rs4268955	2	52288297	2	52307791	0.3	0.3	16.6	
21	rs10167057	rs4260279	2	52288297	2	52307905	0.3	1.0	17.7	
22	rs10167057	rs4328669	2	52288297	2	52308015	0.3	0.8	24.2	
23	rs11687163	rs4664327	2	153171454	2	161294348	0.4	2.6	15.3	
24	rs11687163	rs12692605	2	153171454	2	153198677	0.4	0.5	20.6	
25	rs11687163	rs7423892	2	153171454	2	153213010	0.4	0.1	13.9	
26	rs16849921	rs10197379	2	214061022	2	205912603	18.6	0.0	34.4	[10], [6]
27	rs16849921	rs12694298	2	214061022	2	205913268	18.6	0.2	31.0	[10], [6]
28	rs3773090	rs2168765	3	29609837	3	29663099	0.1	1.4	15.4	[10], [6]
29	rs3773090	rs9818762	3	29609837	3	29680018	0.1	1.3	13.0	[10], [6]
30	rs3773090	rs9832480	3	29609837	3	29680095	0.1	2.5	12.9	[6]
31	rs3773090	rs9832974	3	29609837	3	29680561	0.1	1.2	12.7	[10], [6]
32	rs3773090	rs9838725	3	29609837	3	29681858	0.1	1.2	13.9	[10], [6]

33	rs3773090	rs9858796	3	29609837	3	29691014	0.1	2.3	14.9	[10], [6]
34	rs13069584	rs6809441	3	41516619	3	41494605	0.9	2.7	13.6	[6]
35	rs6599155	rs6809441	3	41489604	3	41494605	2.0	2.7	14.2	
36	rs12054014	rs6809441	3	41490064	3	41494605	0.2	2.7	15.0	[6]
37	rs12054016	rs6809441	3	41490294	3	41494605	0.3	2.7	14.7	[6]
38	rs6809441	rs33916626	3	41494605	3	41539388	2.7	0.5	16.7	[6]
39	rs1455872	rs1568573	3	160416966	3	160458015	0.6	2.7	13.0	
40	rs12638557	rs1568573	3	160423728	3	160458015	0.6	2.7	13.0	
41	rs7646957	rs1568573	3	160429700	3	160458015	0.2	2.7	13.3	
42	rs7624766	rs1568573	3	160429869	3	160458015	0.0	2.7	13.9	
43	rs11729131	rs10017573	4	4460925	4	4463064	6.2	1.7	12.9	
44	rs10017573	rs6816884	4	4463064	4	4473216	1.7	4.4	13.1	
45	rs12507016	rs13149206	4	37099612	4	37140462	0.9	23.3	13.4	[6]
46	rs10433872	rs13149206	4	37107441	4	37140462	0.9	23.3	13.6	[6]
47	rs1346183	rs13149206	4	37115329	4	37140462	0.3	23.3	13.8	[6]
48	rs34855198	rs13149206	4	37134174	4	37140462	3.2	23.3	13.2	
49	rs13149206	rs17547815	4	37140462	4	37140506	23.3	6.4	13.0	
50	rs13149206	rs12502592	4	37140462	4	37140633	23.3	6.7	13.0	
51	rs6846200	rs4277811	4	43207754	4	43208770	4.2	1.0	14.8	
52	rs6846200	rs4309873	4	43207754	4	43210488	4.2	1.6	14.1	
53	rs4706990	rs945238	6	84108065	6	84165856	0.1	1.9	13.6	
54	rs945238	rs6903322	6	84165856	6	84168968	1.9	0.8	15.0	
55	rs945238	rs7745251	6	84165856	6	84171756	1.9	2.6	11.9	
56	rs945238	rs10943933	6	84165856	6	84171930	1.9	0.7	14.3	
57	rs2846530	rs2803101	6	163039045	6	163070021	0.1	0.4	13.9	
58	rs10950088	rs11773150	7	67264560	7	67264811	2.6	0.8	13.3	
59	rs6970199	rs6964677	7	154382776	7	154386052	6.2	2.7	13.5	[10]
60	rs6970199	rs10224868	7	154382776	7	154386472	6.2	3.1	14.2	[10]
61	rs6970199	rs10227971	7	154382776	7	154386680	6.2	2.9	12.7	[10]
62	rs240940	rs2237812	8	28557627	8	28586036	0.0	2.6	15.9	
63	rs4732651	rs2237812	8	28581823	8	28586036	0.0	2.6	18.8	
64	rs10739077	rs7862229	9	5857408	9	5831153	1.2	3.5	13.1	
65	rs6477015	rs7862229	9	5825194	9	5831153	3.0	3.5	14.0	[10]
66	rs7874675	rs7862229	9	5825336	9	5831153	1.8	3.5	13.2	
67	rs4740826	rs7862229	9	5870703	9	5831153	1.4	3.5	12.6	[10]
68	rs7848093	rs7862229	9	5831098	9	5831153	1.8	3.5	14.4	[10]
69	rs1148159	rs2448097	10	26928067	10	26944313	0.2	0.2	16.0	
70	rs2477266	rs2448097	10	26936405	10	26944313	0.2	0.2	16.1	
71	rs17459521	rs892542	10	80496476	10	80498057	3.2	0.7	14.8	[10], [6]
72	rs10902239	rs10902249	11	946484	11	958566	3.0	0.1	12.9	
73	rs10902239	rs11246357	11	946484	11	963980	3.0	0.1	13.3	
74	rs10902239	rs7105477	11	946484	11	964271	3.0	0.1	13.1	
75	rs11027910	rs10219185	11	24458713	11	24504903	2.3	0.2	16.8	[10], [6]
76	rs10500978	rs10219185	11	24493480	11	24504903	2.4	0.2	16.8	[10], [6]
77	rs10500979	rs10219185	11	24493876	11	24504903	0.3	0.2	16.8	[10], [6]
78	rs10500980	rs10219185	11	24494465	11	24504903	1.2	0.2	16.8	[10], [6]
79	rs9971746	rs16941759	12	112337362	12	112367921	3.3	2.3	14.0	
80	rs1169722	rs2668252	12	118651207	12	121668474	5.5	3.7	16.3	
81	rs4770584	rs9318336	13	19586754	13	19588659	0.2	4.2	17.0	
82	rs6563726	rs9315704	13	40131922	13	40140215	1.7	1.0	13.8	
83	rs9315704	rs1885758	13	40140215	13	40145536	1.0	1.1	15.5	
84	rs9315704	rs2324344	13	40140215	13	40151443	1.0	1.7	12.9	
85	rs9315704	rs1160321	13	40140215	13	40155143	1.0	0.8	14.6	
86	rs9315704	rs3858867	13	40140215	13	40155612	1.0	1.4	15.7	
87	rs9315704	rs927515	13	40140215	13	40157114	1.0	0.6	13.4	

88	rs9315704	rs2324346	13	40140215	13	40158827	1.0	1.1	13.0	
89	rs12430163	rs17710571	13	99538377	13	99552723	0.0	0.1	24.9	[10]
90	rs2296999	rs17710571	13	99550351	13	99552723	0.1	0.1	21.2	[10], [6]
91	rs11849674	rs7154773	14	60688023	14	40912999	4.7	2.5	24.3	[10], [6]
92	rs10148587	rs7154773	14	60689160	14	40912999	4.2	2.5	22.0	[10], [6]
93	rs188620	rs7154773	14	40853319	14	40912999	0.8	2.5	23.4	[10], [6]
94	rs10137732	rs7154773	14	60689209	14	40912999	4.4	2.5	27.3	[10], [6]
95	rs6573298	rs7154773	14	40869612	14	40912999	2.1	2.5	18.0	[10]
96	rs7145505	rs7154773	14	40869718	14	40912999	1.6	2.5	17.3	[10]
97	rs8019531	rs7154773	14	40871554	14	40912999	3.1	2.5	31.3	[6]
98	rs11628587	rs7154773	14	40871574	14	40912999	1.8	2.5	17.8	[10]
99	rs11628628	rs7154773	14	40871689	14	40912999	2.0	2.5	17.9	[10]
100	rs8011227	rs7154773	14	40882151	14	40912999	1.9	2.5	36.8	[10]
101	rs7158657	rs7154773	14	40888940	14	40912999	2.9	2.5	30.1	
102	rs10142834	rs7154773	14	40898846	14	40912999	3.0	2.5	31.1	
103	rs17097262	rs7154773	14	40902242	14	40912999	1.0	2.5	34.8	[10], [6]
104	rs1887103	rs7154773	14	40907104	14	40912999	3.1	2.5	24.6	
105	rs7154773	rs8012816	14	40912999	14	40914246	2.5	1.1	26.5	[10]
106	rs7154773	rs10130695	14	40912999	14	40919869	2.5	1.3	34.8	[10], [6]
107	rs7154773	rs1998225	14	40912999	14	40941373	2.5	1.9	18.6	
108	rs7154773	rs8004280	14	40912999	14	60780429	2.5	2.2	18.3	
109	rs7154773	rs1951116	14	40912999	14	40946079	2.5	1.9	15.4	
110	rs7154773	rs1951117	14	40912999	14	60782214	2.5	2.0	18.6	
111	rs7154773	rs7146988	14	40912999	14	60811204	2.5	2.9	13.6	[6]
112	rs7162070	rs16969478	15	39920918	15	39930953	0.2	5.0	14.2	
113	rs1876853	rs16969478	15	39921167	15	39930953	0.3	5.0	14.2	
114	rs8029602	rs16969478	15	39930540	15	39930953	0.3	5.0	14.5	
115	rs16969475	rs16969478	15	39930869	15	39930953	0.2	5.0	14.3	
116	rs7279935	rs1893382	21	17382916	21	17389011	3.5	0.6	14.3	

Table 4: CD results

7.4 Hypertension Results

ID	rs_1	rs_2	chr_1	bp_1	chr_2	bp_2	χ^2_1	χ^2_2	flt_{GSS}	source
1	rs1944328	rs9676116	18	61838457	18	61857065	21.8	2.2	12.0	
2	rs4654792	rs909812	1	22600672	1	22603366	3.2	2.5	12.3	
3	rs2209307	rs11587221	1	89723344	1	89729950	7.8	1.0	14.9	
4	rs4423049	rs11165939	1	98409303	1	98443509	9.5	7.0	13.1	
5	rs4309626	rs10188442	2	133187880	2	133189239	0.5	0.1	14.6	
6	rs13420028	rs10188442	2	133188106	2	133189239	0.6	0.1	14.6	
7	rs16849921	rs10197379	2	214061022	2	205912603	18.6	0.0	37.9	[10], [6]
8	rs16849921	rs12694298	2	214061022	2	205913268	18.6	0.2	35.4	[10], [6]
9	rs12054014	rs6809441	3	41490064	3	41494605	0.2	2.7	11.4	[6]
10	rs6809441	rs33916626	3	41494605	3	41539388	2.7	0.5	18.5	[6]
11	rs6439887	rs11707973	3	139688912	3	139689253	9.4	1.8	13.1	
12	rs959880	rs2314349	3	183088613	3	183091098	5.2	7.8	14.4	[10]
13	rs2314349	rs906719	3	183091098	3	183091144	7.8	4.6	14.2	[10]
14	rs2314349	rs2314348	3	183091098	3	183091330	7.8	5.4	15.1	[10]
15	rs2314349	rs2089588	3	183091098	3	183091474	7.8	5.2	15.1	[10]
16	rs12507016	rs13149206	4	37099612	4	37140462	0.9	23.3	11.5	
17	rs10433872	rs13149206	4	37107441	4	37140462	0.9	23.3	11.5	

18	rs7735940	rs12515142	5	36423931	5	36426411	0.6	0.9	15.3	[10]
19	rs10070418	rs10055279	5	38724924	5	38725043	9.9	4.6	14.4	
20	rs4626363	rs4524536	5	121247900	5	121258740	0.8	0.5	20.8	[10]
21	rs4626363	rs2972345	5	121247900	5	121263708	0.8	2.3	16.9	
22	rs17287085	rs7700706	5	141284815	5	141294362	2.7	17.9	14.8	
23	rs7700706	rs6580205	5	141294362	5	141294691	17.9	4.1	13.4	
24	rs7700706	rs4912787	5	141294362	5	141297123	17.9	4.5	13.2	
25	rs7700706	rs758462	5	141294362	5	141297178	17.9	5.0	13.4	
26	rs1003063	rs9357440	6	9487676	6	9492393	2.1	11.3	13.7	[10]
27	rs9357438	rs9357440	6	9492158	6	9492393	1.1	11.3	24.2	[10]
28	rs6909956	rs4235980	6	67425904	6	67469995	7.5	1.0	13.7	
29	rs1869682	rs4235980	6	67463990	6	67469995	8.3	1.0	16.5	
30	rs9377535	rs4260778	6	104055157	6	104072132	6.4	4.9	14.7	
31	rs11526287	rs2250603	7	135297419	7	135315708	0.2	2.7	19.1	
32	rs2919408	rs11782342	8	73739252	8	69281738	5.1	14.0	13.2	
33	rs4571768	rs11782342	8	73743348	8	69281738	2.2	14.0	11.2	
34	rs4307386	rs11782342	8	73743751	8	69281738	2.1	14.0	11.0	
35	rs11780806	rs11782342	8	73771329	8	69281738	0.4	14.0	13.4	
36	rs11782342	rs10096807	8	69281738	8	69283209	14.0	2.0	16.6	[6]
37	rs11782342	rs6994225	8	69281738	8	69299056	14.0	0.0	12.3	
38	rs2978169	rs3018857	8	95023498	8	95023826	0.7	11.0	11.6	
39	rs7002915	rs4437686	8	114310388	8	114357413	0.9	2.9	15.6	
40	rs7012271	rs4437686	8	114317672	8	114357413	0.8	2.9	16.7	
41	rs2447183	rs2469997	8	120352978	8	120353267	0.5	1.4	19.4	[10]
42	rs2469996	rs2469997	8	120353011	8	120353267	0.2	1.4	19.4	[10]
43	rs2469997	rs6469823	8	120353267	8	120353984	1.4	0.3	19.1	[10]
44	rs2469997	rs2447179	8	120353267	8	120355775	1.4	0.4	19.4	[10]
45	rs2469997	rs2447178	8	120353267	8	120356188	1.4	0.3	19.4	[10]
46	rs2469997	rs2470002	8	120353267	8	120357424	1.4	0.2	19.1	[10]
47	rs2469997	rs2470025	8	120353267	8	120364727	1.4	0.3	19.4	[10]
48	rs2469997	rs2447169	8	120353267	8	120365063	1.4	0.3	19.1	[10]
49	rs2469997	rs2470026	8	120353267	8	120365112	1.4	0.2	17.6	[10]
50	rs2469997	rs2447168	8	120353267	8	120365613	1.4	0.3	19.8	[10]
51	rs2469997	rs2470040	8	120353267	8	120385452	1.4	0.4	18.0	[10]
52	rs6421008	rs7827545	8	135512645	8	135566567	1.5	0.6	36.1	[10], [6]
53	rs7386230	rs7827545	8	135526459	8	135566567	0.1	0.6	19.3	[10], [6]
54	rs6988000	rs7827545	8	135559534	8	135566567	2.6	0.6	16.2	[10]
55	rs6578234	rs7827545	8	135566363	8	135566567	0.6	0.6	43.1	[10]
56	rs7827545	rs1372662	8	135566567	8	135567046	0.6	0.6	43.4	[10]
57	rs7827545	rs6578237	8	135566567	8	135569358	0.6	0.3	40.1	[10]
58	rs7827545	rs7846013	8	135566567	8	135569967	0.6	0.8	42.1	[10], [6]
59	rs7827545	rs6983560	8	135566567	8	135584553	0.6	1.0	12.9	[10]
60	rs7827545	rs12679315	8	135566567	8	135624837	0.6	6.8	15.4	[10], [6]
61	rs7827545	rs16905198	8	135566567	8	135626272	0.6	0.3	12.4	[10], [6]
62	rs10816425	rs2035783	9	109477710	9	109479279	1.9	9.1	15.5	
63	rs9942941	rs2035783	9	109479134	9	109479279	2.0	9.1	15.4	
64	rs1392331	rs10219460	12	33249832	12	33328392	0.2	0.1	11.0	
65	rs10844474	rs10219460	12	33269608	12	33328392	0.1	0.1	11.7	
66	rs10459094	rs10219460	12	33275884	12	33328392	0.2	0.1	11.6	
67	rs1445311	rs10219460	12	33279859	12	33328392	0.6	0.1	11.1	
68	rs7973393	rs10219460	12	33282226	12	33328392	0.1	0.1	11.6	
69	rs7311281	rs1688786	12	67412742	12	70380015	1.0	0.2	17.6	[10], [6]
70	rs1688786	rs10879068	12	70380015	12	67431375	0.2	1.7	20.8	[10], [6]
71	rs1958305	rs17184408	14	24273124	14	24282020	33.4	0.5	16.4	[10], [6]
72	rs1958305	rs12100601	14	24273124	14	24284173	33.4	1.9	12.7	

73	rs11849674	rs7154773	14	60688023	14	40912999	4.7	2.5	30.1	[10]
74	rs10148587	rs7154773	14	60689160	14	40912999	4.2	2.5	26.1	[10]
75	rs188620	rs7154773	14	40853319	14	40912999	0.8	2.5	27.2	[10], [6]
76	rs10137732	rs7154773	14	60689209	14	40912999	4.4	2.5	32.4	[10]
77	rs6573298	rs7154773	14	40869612	14	40912999	2.1	2.5	22.6	[10]
78	rs7145505	rs7154773	14	40869718	14	40912999	1.6	2.5	22.2	[10]
79	rs8019531	rs7154773	14	40871554	14	40912999	3.1	2.5	33.0	[6]
80	rs11628587	rs7154773	14	40871574	14	40912999	1.8	2.5	22.9	[10]
81	rs11628628	rs7154773	14	40871689	14	40912999	2.0	2.5	22.9	[10]
82	rs8011227	rs7154773	14	40882151	14	40912999	1.9	2.5	43.3	[10]
83	rs7158657	rs7154773	14	40888940	14	40912999	2.9	2.5	30.4	
84	rs10142834	rs7154773	14	40898846	14	40912999	3.0	2.5	33.0	
85	rs17097262	rs7154773	14	40902242	14	40912999	1.0	2.5	41.7	[10], [6]
86	rs1887103	rs7154773	14	40907104	14	40912999	3.1	2.5	30.1	
87	rs7154773	rs8012816	14	40912999	14	40914246	2.5	1.1	34.7	[10]
88	rs7154773	rs10130695	14	40912999	14	40919869	2.5	1.3	40.2	[10], [6]
89	rs7154773	rs1998225	14	40912999	14	40941373	2.5	1.9	16.6	
90	rs7154773	rs8004280	14	40912999	14	60780429	2.5	2.2	16.6	
91	rs7154773	rs1951116	14	40912999	14	40946079	2.5	1.9	15.4	
92	rs7154773	rs1951117	14	40912999	14	60782214	2.5	2.0	17.1	
93	rs2757527	rs2757528	14	100660933	14	100661077	1.2	0.0	16.6	
94	rs2757527	rs2766696	14	100660933	14	100661320	1.2	0.1	17.8	
95	rs2414093	rs934635	15	51478285	15	28308840	1.7	0.5	13.6	[10]
96	rs746655	rs921535	15	74107677	15	74111343	4.5	9.6	26.3	[10]
97	rs921535	rs999742	15	74111343	15	74115517	9.6	14.8	17.7	[10]
98	rs7243021	rs6417101	18	45657983	18	45672819	0.7	1.2	15.0	
99	rs8085875	rs6417101	18	45659367	18	45672819	0.2	1.2	20.2	
100	rs8098626	rs6417101	18	45667057	18	45672819	1.7	1.2	15.0	
101	rs6417101	rs7231864	18	45672819	18	45698356	1.2	0.1	11.6	
102	rs6417101	rs4940307	18	45672819	18	45700303	1.2	0.0	11.3	
103	rs1944328	rs8091006	18	61838457	18	61842934	21.8	0.3	14.4	[6]
104	rs1944328	rs9675798	18	61838457	18	61856967	21.8	1.0	13.4	
105	rs9305551	rs12482676	21	35965084	21	35970712	0.4	6.7	13.5	
106	rs2300390	rs12482676	21	35969515	21	35970712	0.6	6.7	16.3	
107	rs2300391	rs12482676	21	35969900	21	35970712	0.4	6.7	16.0	

Table 5: HT results

7.5 Rhuemetoid Arthritis Results

ID	rs_1	rs_2	chr_1	bp_1	chr_2	bp_2	χ_1^2	χ_2^2	flt_{GSS}	source
1	rs4654792	rs909812	1	22600672	1	22603366	3.2	2.5	11.6	
2	rs10923966	rs12037204	1	116135466	1	116167796	2.0	2.8	13.5	
3	rs11582522	rs12037204	1	116137772	1	116167796	0.6	2.8	11.7	
4	rs4240547	rs12037204	1	116151255	1	116167796	0.9	2.8	13.6	[10], [6]
5	rs10924064	rs12037204	1	116164698	1	116167796	1.9	2.8	16.5	[10]
6	rs12039479	rs12037204	1	116167482	1	116167796	2.5	2.8	20.2	[10]
7	rs12037204	rs17034079	1	116167796	1	116170761	2.8	1.9	20.2	[10]
8	rs12037204	rs7523839	1	116167796	1	116173080	2.8	0.1	13.0	[6]
9	rs12037204	rs3010380	1	116167796	1	116188971	2.8	0.1	12.1	[6]
10	rs12037204	rs41447448	1	116167796	1	116191145	2.8	3.0	18.1	[10]
11	rs1343295	rs7543540	1	166306753	1	166308285	2.8	3.6	24.7	[10]

12	rs1343295	rs6693244	1	166306753	1	166308823	2.8	3.2	23.1	[10]
13	rs6425407	rs4370732	1	148277619	1	148281500	7.4	6.3	12.9	
14	rs6425407	rs12732164	1	148277619	1	148282752	7.4	6.8	12.9	
15	rs2808250	rs2809345	1	200592547	1	200596263	2.1	0.6	15.2	[13]
16	rs2809345	rs7529962	1	200596263	1	200596977	0.6	0.6	18.2	
17	rs16984594	rs16984606	2	18451111	2	18451405	3.2	3.1	23.5	[10]
18	rs3768666	rs6737027	2	29005870	2	29070805	1.4	2.4	16.8	
19	rs7601413	rs6737027	2	28774193	2	29070805	1.7	2.4	15.9	
20	rs7601934	rs6737027	2	29032673	2	29070805	1.8	2.4	14.6	
21	rs6547881	rs6737027	2	29032746	2	29070805	1.7	2.4	16.6	
22	rs7597048	rs6737027	2	29066756	2	29070805	1.8	2.4	17.1	
23	rs6756762	rs9653500	2	64720795	2	64820616	1.1	0.4	12.3	
24	rs12713512	rs9653500	2	64720870	2	64820616	1.4	0.4	13.2	
25	rs6756813	rs9653500	2	64721016	2	64820616	1.2	0.4	13.7	
26	rs12104832	rs9653500	2	64722470	2	64820616	1.3	0.4	12.9	
27	rs12105600	rs9653500	2	64722511	2	64820616	1.4	0.4	13.9	
28	rs6759598	rs9653500	2	64726801	2	64820616	1.3	0.4	13.5	
29	rs9309362	rs9653500	2	64798538	2	64820616	0.0	0.4	23.5	[10]
30	rs10184493	rs9653500	2	64800765	2	64820616	0.3	0.4	28.3	[10]
31	rs3770729	rs9653500	2	64813491	2	64820616	0.2	0.4	31.1	[10]
32	rs4663053	rs10496658	2	127638990	2	127663497	2.0	1.1	11.1	
33	rs4663054	rs10496658	2	127639995	2	127663497	1.9	1.1	11.6	
34	rs7425345	rs10496658	2	127640396	2	127663497	2.0	1.1	11.7	
35	rs3795874	rs10496658	2	127656383	2	127663497	1.9	1.1	11.4	
36	rs4664633	rs4664635	2	153811011	2	153830935	1.5	0.2	17.8	
37	rs7601781	rs4664635	2	153814233	2	153830935	1.2	0.2	24.8	[10], [6]
38	rs16849921	rs10197379	2	214061022	2	205912603	18.6	0.0	38.9	[10], [6]
39	rs16849921	rs12694298	2	214061022	2	205913268	18.6	0.2	38.0	[10], [6]
40	rs1917946	rs4467334	2	235517079	2	235531268	2.0	2.9	14.0	
41	rs7605520	rs4467334	2	235519242	2	235531268	1.5	2.9	13.4	
42	rs6711637	rs4467334	2	235527499	2	235531268	1.9	2.9	14.7	
43	rs11708019	rs1605705	3	7163913	3	7290044	0.3	2.1	14.1	[10]
44	rs11710946	rs1605705	3	7180535	3	7290044	5.8	2.1	12.4	[10]
45	rs951557	rs1605705	3	7269035	3	7290044	13.9	2.1	19.9	[10], [6]
46	rs2136152	rs1605705	3	7282402	3	7290044	2.6	2.1	52.2	[13], [10]
47	rs4686119	rs1605705	3	7282739	3	7290044	3.8	2.1	51.8	[10], [6]
48	rs908465	rs1605705	3	7283715	3	7290044	3.6	2.1	53.0	[10], [6]
49	rs1605705	rs9809928	3	7290044	3	7302350	2.1	3.4	51.0	[10], [6]
50	rs1605705	rs6443099	3	7290044	3	7320514	2.1	8.4	12.5	[10]
51	rs1605705	rs1876614	3	7290044	3	7321289	2.1	0.9	39.7	[10]
52	rs1605705	rs1396402	3	7290044	3	7330517	2.1	0.4	41.2	[10], [6]
53	rs1605705	rs7632044	3	7290044	3	7335947	2.1	1.7	39.2	[10]
54	rs13069584	rs6809441	3	41516619	3	41494605	0.9	2.7	15.5	[6]
55	rs6599155	rs6809441	3	41489604	3	41494605	2.0	2.7	16.0	
56	rs12054014	rs6809441	3	41490064	3	41494605	0.2	2.7	15.9	[6]
57	rs12054016	rs6809441	3	41490294	3	41494605	0.3	2.7	15.6	[6]
58	rs6809441	rs33916626	3	41494605	3	41539388	2.7	0.5	16.1	[6]
59	rs4312689	rs2128361	3	45528633	3	45533224	2.7	2.5	11.2	
60	rs17073902	rs17073922	3	65813197	3	65817183	0.7	3.3	11.6	
61	rs3772556	rs2171146	3	105249211	3	105252377	3.7	4.6	18.5	[10], [6]
62	rs3772556	rs9811466	3	105249211	3	105254709	3.7	4.5	19.9	[10], [6]
63	rs3772556	rs10933821	3	105249211	3	105266536	3.7	1.6	18.2	[10], [6]
64	rs4308237	rs4538338	3	139942366	3	139953635	1.2	0.6	32.6	[10], [6]
65	rs9856460	rs4538338	3	139942453	3	139953635	0.8	0.6	36.2	[10], [6]
66	rs2350514	rs4538338	3	139951382	3	139953635	0.3	0.6	37.4	[10], [6]

67	rs935972	rs1010342	4	1045171	4	1068819	2.9	1.5	14.8	
68	rs935970	rs1010342	4	1045323	4	1068819	2.6	1.5	16.0	[10]
69	rs6839931	rs1010342	4	1053172	4	1068819	1.9	1.5	18.4	[10]
70	rs10084890	rs1010342	4	1053934	4	1068819	1.7	1.5	21.2	[10]
71	rs12645644	rs1010342	4	1054159	4	1068819	2.0	1.5	17.2	[10]
72	rs7669622	rs1010342	4	1058659	4	1068819	1.9	1.5	22.1	[10]
73	rs6599278	rs1010342	4	1058798	4	1068819	1.5	1.5	20.1	[10]
74	rs6827357	rs1010342	4	1059202	4	1068819	1.8	1.5	21.9	[10]
75	rs6599280	rs1010342	4	1063818	4	1068819	1.9	1.5	20.8	[10]
76	rs2290409	rs1010342	4	1067249	4	1068819	1.9	1.5	18.3	[10], [6]
77	rs2290408	rs1010342	4	1067267	4	1068819	1.8	1.5	22.1	[10]
78	rs2014318	rs1010342	4	1068778	4	1068819	1.8	1.5	24.4	[10]
79	rs1010342	rs11939380	4	1068819	4	1086871	1.5	1.7	18.7	[10], [6]
80	rs1010342	rs4045481	4	1068819	4	1090625	1.5	0.9	14.7	[10]
81	rs1010342	rs651017	4	1068819	4	1091062	1.5	1.7	21.0	[10]
82	rs11943360	rs10017573	4	4440520	4	4463064	4.1	1.7	13.0	
83	rs11729131	rs10017573	4	4460925	4	4463064	6.2	1.7	16.3	[6]
84	rs10017573	rs6816884	4	4463064	4	4473216	1.7	4.4	14.1	
85	rs10017573	rs6841963	4	4463064	4	4473835	1.7	2.4	16.1	[6]
86	rs10017573	rs6853914	4	4463064	4	4499020	1.7	5.4	11.5	
87	rs34855198	rs13149206	4	37134174	4	37140462	3.2	23.3	11.1	
88	rs13149206	rs17547815	4	37140462	4	37140506	23.3	6.4	12.3	
89	rs13149206	rs12502592	4	37140462	4	37140633	23.3	6.7	12.2	
90	rs6846200	rs4277811	4	43207754	4	43208770	4.2	1.0	17.2	
91	rs6846200	rs4309873	4	43207754	4	43210488	4.2	1.6	14.7	
92	rs6850108	rs7679010	4	48078593	4	48083885	1.0	1.6	22.1	[10]
93	rs4589663	rs7679010	4	48080573	4	48083885	1.4	1.6	21.0	[10]
94	rs7679010	rs10007288	4	48083885	4	48090135	1.6	1.3	20.0	[10]
95	rs7679010	rs13118073	4	48083885	4	48094275	1.6	1.3	16.4	
96	rs7679010	rs1466998	4	48083885	4	48097400	1.6	1.3	18.3	[10]
97	rs12509680	rs10005635	4	114754586	4	114773178	3.0	2.1	15.2	[6]
98	rs6834503	rs10005635	4	114771175	4	114773178	1.4	2.1	21.4	[10]
99	rs1447322	rs10520443	4	176910200	4	176912425	0.0	3.8	14.2	
100	rs6831911	rs4532278	4	177504092	4	177505933	1.0	0.0	29.2	[10], [6]
101	rs6831911	rs4274891	4	177504092	4	177506157	1.0	0.1	27.9	[10], [6]
102	rs6831911	rs7657018	4	177504092	4	177512358	1.0	1.2	22.9	[10]
103	rs6831911	rs11737773	4	177504092	4	177521043	1.0	1.9	25.8	[10]
104	rs6831911	rs17810380	4	177504092	4	177528334	1.0	3.1	24.6	[10]
105	rs7711236	rs871879	5	3675933	5	3677182	1.2	0.9	14.5	
106	rs4703937	rs417769	5	71797851	5	71811478	2.6	7.1	12.7	
107	rs937622	rs6889735	5	76399282	5	76427462	6.6	4.5	14.1	[10], [6]
108	rs7711901	rs6889735	5	76400285	5	76427462	7.1	4.5	14.8	[10], [6]
109	rs7727604	rs6889735	5	76403145	5	76427462	6.2	4.5	15.4	[10], [6]
110	rs11242569	rs12656675	5	105399292	5	105405016	1.6	1.4	15.1	
111	rs9271850	rs3129768	6	0	6	0	4.4	1.1	18.1	
112	rs9296318	rs10947857	6	39672484	6	40111515	0.9	2.9	23.3	[10]
113	rs4594945	rs10947857	6	39954159	6	40111515	1.3	2.9	27.0	[10]
114	rs10456478	rs10947857	6	39678867	6	40111515	1.0	2.9	21.5	[10]
115	rs2894387	rs10947857	6	39680754	6	40111515	0.9	2.9	23.3	[10]
116	rs10807222	rs10947857	6	39692893	6	40111515	1.1	2.9	22.6	[10]
117	rs10947849	rs10947857	6	39722691	6	40111515	5.7	2.9	21.8	[10], [6]
118	rs1007026	rs10947857	6	40039031	6	40111515	1.2	2.9	33.2	[10]
119	rs847777	rs10947857	6	40055754	6	40111515	4.5	2.9	17.6	[6]
120	rs10947854	rs10947857	6	40066516	6	40111515	2.0	2.9	31.3	[10]
121	rs10807224	rs10947857	6	40072712	6	40111515	2.4	2.9	28.6	[10]

122	rs6912289	rs10947857	6	40079519	6	40111515	2.1	2.9	33.8	[10]
123	rs847747	rs10947857	6	40080069	6	40111515	4.8	2.9	19.2	[10], [6]
124	rs9369170	rs10947857	6	40083496	6	40111515	4.5	2.9	27.2	[10], [6]
125	rs761798	rs10947857	6	40087514	6	40111515	3.2	2.9	15.8	[6]
126	rs9471244	rs10947857	6	40092331	6	40111515	3.5	2.9	20.3	[10], [6]
127	rs10947855	rs10947857	6	40096889	6	40111515	3.2	2.9	40.2	[10], [6]
128	rs6936016	rs10947857	6	40097013	6	40111515	3.7	2.9	20.7	[10], [6]
129	rs2091113	rs10947857	6	40099385	6	40111515	2.4	2.9	39.8	[10]
130	rs10947856	rs10947857	6	40111427	6	40111515	2.8	2.9	38.8	[10]
131	rs10947857	rs940357	6	40111515	6	40112662	2.9	3.5	39.4	[10]
132	rs10947857	rs9296320	6	40111515	6	40118298	2.9	2.2	17.7	[6]
133	rs13205512	rs9394893	6	41969530	6	42257161	1.9	0.8	13.8	[13], [10], [6]
134	rs1753505	rs1891698	6	72604896	6	72633028	8.5	6.0	22.6	[10], [6]
135	rs9293855	rs1891698	6	72621500	6	72633028	5.9	6.0	44.3	[13], [10]
136	rs945238	rs6903322	6	84165856	6	84168968	1.9	0.8	19.8	
137	rs945238	rs6454329	6	84165856	6	84171532	1.9	0.5	16.9	
138	rs945238	rs7745251	6	84165856	6	84171756	1.9	2.6	19.1	[6]
139	rs945238	rs10943933	6	84165856	6	84171930	1.9	0.7	20.1	
140	rs945238	rs9449623	6	84165856	6	84180355	1.9	0.9	14.6	
141	rs6569717	rs1338905	6	131329259	6	131361935	1.8	3.4	12.8	
142	rs6908917	rs1338905	6	131355864	6	131361935	4.9	3.4	13.0	
143	rs2846530	rs2803101	6	163039045	6	163070021	0.1	0.4	14.2	[13]
144	rs10237371	rs12536071	7	42425753	7	42468346	1.8	2.5	13.0	
145	rs10488181	rs12536071	7	42462596	7	42468346	1.8	2.5	17.2	
146	rs6969107	rs12536071	7	42463367	7	42468346	3.2	2.5	14.2	
147	rs12536071	rs12531052	7	42468346	7	42468515	2.5	2.3	20.1	[10]
148	rs12531615	rs6947662	7	82724539	7	82725618	0.2	0.2	17.7	[10]
149	rs12531615	rs17157155	7	82724539	7	82729409	0.2	0.2	19.5	[10]
150	rs12531615	rs17157173	7	82724539	7	82735376	0.2	0.2	17.4	[10]
151	rs12531615	rs17157177	7	82724539	7	82735618	0.2	0.3	19.2	[10]
152	rs12531615	rs16887406	7	82724539	7	82735658	0.2	0.3	19.6	[10]
153	rs9942681	rs2106311	7	126187739	7	126229390	1.8	4.0	22.6	[10]
154	rs12154335	rs2106311	7	126192371	7	126229390	2.2	4.0	20.7	[10]
155	rs1361990	rs2106311	7	126218229	7	126229390	2.5	4.0	26.1	[10]
156	rs2106311	rs2106312	7	126229390	7	126229457	4.0	3.2	22.0	[10]
157	rs2106311	rs2299476	7	126229390	7	126244330	4.0	2.4	26.1	[10]
158	rs2106311	rs6467092	7	126229390	7	126262995	4.0	1.9	23.1	[10]
159	rs2106311	rs728600	7	126229390	7	126278808	4.0	2.1	23.9	[10]
160	rs2106311	rs1419484	7	126229390	7	126286601	4.0	1.6	24.2	[10]
161	rs2106311	rs7792592	7	126229390	7	126295941	4.0	1.9	23.7	[10]
162	rs240940	rs2237812	8	28557627	8	28586036	0.0	2.6	14.3	
163	rs4732651	rs2237812	8	28581823	8	28586036	0.0	2.6	18.6	[13]
164	rs2237812	rs4732873	8	28586036	8	28601554	2.6	0.1	14.8	
165	rs10089599	rs4733372	8	32598221	8	32605388	0.1	0.1	24.1	
166	rs4733372	rs4733373	8	32605388	8	32605583	0.1	0.1	24.8	[10]
167	rs10099080	rs11984645	8	50357376	8	55069305	0.8	50.4	16.3	[10], [6]
168	rs6473901	rs11984645	8	50386264	8	55069305	1.4	50.4	16.5	[10], [6]
169	rs6473903	rs11984645	8	50388147	8	55069305	0.0	50.4	15.6	[10], [6]
170	rs6983650	rs11984645	8	50406226	8	55069305	0.4	50.4	17.0	[10], [6]
171	rs7844299	rs11984645	8	50430790	8	55069305	0.9	50.4	16.6	[10], [6]
172	rs16919784	rs11984645	8	55063538	8	55069305	3.0	50.4	13.1	[10]
173	rs11984645	rs4737503	8	55069305	8	55071319	50.4	2.7	13.4	[10], [6]
174	rs3763523	rs2958371	8	71033469	8	71077195	0.5	2.4	17.6	[10], [6]
175	rs4074358	rs2958371	8	71046411	8	71077195	2.4	2.4	34.5	[10]
176	rs2977984	rs2958371	8	71073672	8	71077195	2.3	2.4	34.0	[10]

177	rs2958371	rs7017499	8	71077195	8	71083453	2.4	0.1	28.3	[10], [6]
178	rs2958371	rs10504469	8	71077195	8	71089017	2.4	3.3	32.8	[10]
179	rs2958371	rs11993276	8	71077195	8	71095359	2.4	3.2	28.1	[10]
180	rs2958371	rs7015854	8	71077195	8	71101356	2.4	0.3	20.1	[10], [6]
181	rs2958371	rs2977988	8	71077195	8	71146050	2.4	0.2	23.1	[10], [6]
182	rs2958371	rs2926702	8	71077195	8	71167994	2.4	2.0	24.5	[10], [6]
183	rs2958371	rs12676045	8	71077195	8	71259947	2.4	0.4	23.4	[10], [6]
184	rs2958371	rs10504473	8	71077195	8	71260332	2.4	0.3	23.4	[10], [6]
185	rs2958371	rs11998005	8	71077195	8	71335876	2.4	3.5	25.1	[10]
186	rs2958371	rs16937001	8	71077195	8	71340989	2.4	4.5	23.3	[10], [6]
187	rs2958371	rs16937002	8	71077195	8	71341140	2.4	4.3	25.5	[10]
188	rs10983142	rs7868322	9	119174267	9	119176906	4.4	7.1	11.3	
189	rs7868322	rs3747830	9	119176906	9	119185987	7.1	4.3	11.4	
190	rs7868322	rs9299239	9	119176906	9	119187220	7.1	4.8	11.7	
191	rs7868322	rs6478237	9	119176906	9	119192516	7.1	3.2	11.6	
192	rs6602681	rs10737079	10	13783685	10	13785400	1.2	0.9	13.2	
193	rs10827054	rs7910994	10	32726960	10	33096734	2.8	2.5	15.7	[10]
194	rs2505382	rs7910994	10	32761062	10	33096734	1.3	2.5	13.7	[10]
195	rs11008969	rs7910994	10	32834243	10	33096734	1.3	2.5	13.4	[10]
196	rs11009021	rs7910994	10	32951737	10	33096734	0.7	2.5	16.7	[10]
197	rs9418016	rs7910994	10	32966615	10	33096734	1.3	2.5	15.1	[10]
198	rs7910994	rs2230395	10	33096734	10	33211227	2.5	1.0	11.4	
199	rs7910994	rs2230394	10	33096734	10	33217110	2.5	1.4	11.4	
200	rs10883365	rs1548964	10	101287764	10	101289653	2.7	1.9	21.7	
201	rs10883367	rs1548964	10	101287990	10	101289653	2.6	1.9	21.2	
202	rs1548964	rs1548962	10	101289653	10	101289735	1.9	2.4	22.0	
203	rs1548964	rs10883371	10	101289653	10	101292455	1.9	2.2	18.7	
204	rs7896275	rs4256909	10	114081995	10	114093585	1.1	0.1	32.5	[10], [6]
205	rs4256909	rs7898941	10	114093585	10	114094931	0.1	9.1	16.6	[10], [6]
206	rs4256909	rs11195939	10	114093585	10	114142104	0.1	6.0	11.2	
207	rs3825075	rs6598060	11	217140	11	243987	4.1	3.4	15.4	
208	rs6421986	rs6598060	11	221659	11	243987	5.8	3.4	16.7	
209	rs6598060	rs7116130	11	243987	11	244129	3.4	3.7	17.5	[10]
210	rs6598060	rs1128322	11	243987	11	244197	3.4	3.3	21.9	[10]
211	rs2334501	rs10768666	11	1620784	11	1632411	0.9	1.1	12.8	
212	rs11027910	rs10219185	11	24458713	11	24504903	2.3	0.2	14.5	[10], [6]
213	rs10500978	rs10219185	11	24493480	11	24504903	2.4	0.2	14.5	[10], [6]
214	rs10500979	rs10219185	11	24493876	11	24504903	0.3	0.2	14.1	[10], [6]
215	rs10500980	rs10219185	11	24494465	11	24504903	1.2	0.2	14.7	[10], [6]
216	rs11604421	rs4988327	11	64363232	11	64413928	0.1	4.5	13.5	[10]
217	rs11606508	rs4988327	11	64392274	11	64413928	0.2	4.5	19.6	[10], [6]
218	rs4376999	rs10842357	12	24620969	12	24628433	2.1	2.4	13.5	
219	rs4963762	rs10842357	12	24622160	12	24628433	2.4	2.4	17.0	[10]
220	rs10785426	rs7486175	12	43703858	12	43716240	1.2	0.8	19.9	[10]
221	rs7295668	rs7486175	12	43713372	12	43716240	1.1	0.8	21.8	[10]
222	rs6582453	rs7486175	12	43714825	12	43716240	1.1	0.8	22.6	[10]
223	rs6582454	rs7486175	12	43714906	12	43716240	1.1	0.8	22.6	[10]
224	rs7486175	rs1995339	12	43716240	12	43718844	0.8	1.0	24.0	[10]
225	rs6490403	rs7322082	13	29954105	13	29955805	0.0	1.6	14.2	
226	rs6490403	rs7997274	13	29954105	13	29957085	0.0	1.8	14.1	[13]
227	rs6490403	rs7332561	13	29954105	13	29962516	0.0	2.0	15.0	[10], [6]
228	rs6490403	rs7319196	13	29954105	13	29967550	0.0	1.8	14.4	
229	rs9548796	rs9315704	13	40094490	13	40140215	2.7	1.0	15.2	
230	rs1022808	rs9315704	13	40094679	13	40140215	1.3	1.0	14.8	
231	rs4545673	rs9315704	13	40118499	13	40140215	7.1	1.0	14.4	

232	rs6563726	rs9315704	13	40131922	13	40140215	1.7	1.0	20.4	
233	rs9315704	rs1885758	13	40140215	13	40145536	1.0	1.1	21.5	
234	rs9315704	rs2324344	13	40140215	13	40151443	1.0	1.7	15.0	
235	rs9315704	rs1160321	13	40140215	13	40155143	1.0	0.8	15.9	
236	rs9315704	rs3858867	13	40140215	13	40155612	1.0	1.4	16.8	
237	rs9315704	rs927515	13	40140215	13	40157114	1.0	0.6	14.4	
238	rs9315704	rs2324346	13	40140215	13	40158827	1.0	1.1	13.0	
239	rs9315704	rs4567589	13	40140215	13	40160541	1.0	1.2	13.6	
240	rs9315704	rs9566462	13	40140215	13	40169423	1.0	2.2	13.1	
241	rs7327105	rs9591470	13	34144716	13	34146991	1.4	1.1	14.7	
242	rs9591470	rs1407760	13	34146991	13	34149445	1.1	1.1	13.5	[10]
243	rs7336478	rs2038825	13	46675360	13	46683136	0.1	0.0	15.0	
244	rs12430163	rs17710571	13	99538377	13	99552723	0.0	0.1	21.1	[10]
245	rs2296999	rs17710571	13	99550351	13	99552723	0.1	0.1	16.8	[10], [6]
246	rs279937	rs279936	13	103704077	13	103704608	4.0	2.8	23.8	[10]
247	rs279937	rs188096	13	103704077	13	103705044	4.0	2.3	23.8	[10]
248	rs279937	rs157266	13	103704077	13	103712499	4.0	1.9	19.3	[10]
249	rs12427557	rs7328544	13	104369267	13	104369448	5.9	5.7	21.5	[10]
250	rs17731911	rs12873360	13	89294665	13	89298845	0.2	0.0	14.5	
251	rs7152370	rs10483596	14	46735956	14	46740909	2.8	5.8	16.5	
252	rs10498441	rs2144977	14	52544224	14	52561350	0.4	2.2	28.3	[10], [6]
253	rs1956286	rs2144977	14	52553691	14	52561350	0.9	2.2	23.8	[10], [6]
254	rs17125074	rs2144977	14	52559041	14	52561350	1.8	2.2	40.7	[10]
255	rs2144977	rs1998093	14	52561350	14	52563297	2.2	1.3	41.0	[10]
256	rs2144977	rs17125124	14	52561350	14	52572995	2.2	0.5	41.6	[10], [6]
257	rs11849674	rs7154773	14	60688023	14	40912999	4.7	2.5	25.1	[10]
258	rs10148587	rs7154773	14	60689160	14	40912999	4.2	2.5	22.4	[10]
259	rs188620	rs7154773	14	40853319	14	40912999	0.8	2.5	23.6	[10], [6]
260	rs10137732	rs7154773	14	60689209	14	40912999	4.4	2.5	28.8	[10], [6]
261	rs6573298	rs7154773	14	40869612	14	40912999	2.1	2.5	22.1	[10]
262	rs7145505	rs7154773	14	40869718	14	40912999	1.6	2.5	21.8	[10]
263	rs8019531	rs7154773	14	40871554	14	40912999	3.1	2.5	33.1	[6]
264	rs11628587	rs7154773	14	40871574	14	40912999	1.8	2.5	22.9	[10]
265	rs11628628	rs7154773	14	40871689	14	40912999	2.0	2.5	23.2	[10]
266	rs8011227	rs7154773	14	40882151	14	40912999	1.9	2.5	39.5	[10]
267	rs7158657	rs7154773	14	40888940	14	40912999	2.9	2.5	33.5	
268	rs10142834	rs7154773	14	40898846	14	40912999	3.0	2.5	34.9	
269	rs17097262	rs7154773	14	40902242	14	40912999	1.0	2.5	36.8	[10], [6]
270	rs1887103	rs7154773	14	40907104	14	40912999	3.1	2.5	25.3	
271	rs7154773	rs8012816	14	40912999	14	40914246	2.5	1.1	31.3	[10]
272	rs7154773	rs10130695	14	40912999	14	40919869	2.5	1.3	37.3	[13], [10], [6]
273	rs7154773	rs1998225	14	40912999	14	40941373	2.5	1.9	18.1	
274	rs7154773	rs8004280	14	40912999	14	60780429	2.5	2.2	17.8	
275	rs7154773	rs1951116	14	40912999	14	40946079	2.5	1.9	15.0	
276	rs7154773	rs1951117	14	40912999	14	60782214	2.5	2.0	18.2	
277	rs8035210	rs8034355	15	73230766	15	73231011	2.7	1.3	14.3	[10]
278	rs8034178	rs8034355	15	73230849	15	73231011	1.4	1.3	19.8	[10]
279	rs8034355	rs4777568	15	73231011	15	73232225	1.3	4.3	14.3	[10]
280	rs8034355	rs1900899	15	73231011	15	73234080	1.3	3.0	14.6	[10]
281	rs8034355	rs7172629	15	73231011	15	50112864	1.3	0.1	12.3	[10]
282	rs8034355	rs11858307	15	73231011	15	50115951	1.3	0.2	11.9	
283	rs8034355	rs7174900	15	73231011	15	50121893	1.3	1.5	13.1	[10]
284	rs2881439	rs7205378	16	7283490	16	7283842	1.0	0.9	15.4	
285	rs4238755	rs1420247	16	52746089	16	52748342	8.7	9.9	40.1	[13], [10]
286	rs4784244	rs1420247	16	52748247	16	52748342	0.1	9.9	26.1	[10], [6]

287	rs1420247	rs1420248	16	52748342	16	52748361	9.9	6.7	35.1	[10], [6]
288	rs1420247	rs12446384	16	52748342	16	52768754	9.9	12.4	22.4	[10], [6]
289	rs1420247	rs1362413	16	52748342	16	52776657	9.9	1.2	15.2	[10]
290	rs1420247	rs7499961	16	52748342	16	52778697	9.9	11.9	23.4	[10], [6]
291	rs9302946	rs11077601	17	70346698	17	70350140	4.1	5.7	14.8	
292	rs12452792	rs11077601	17	70349624	17	70350140	5.7	5.7	24.2	[10]
293	rs11077601	rs8065637	17	70350140	17	70355387	5.7	5.1	15.8	
294	rs11077601	rs9916746	17	70350140	17	70355568	5.7	5.3	15.9	
295	rs11077601	rs3744313	17	70350140	17	70358058	5.7	6.3	19.7	
296	rs3923514	rs901064	17	78515210	17	78596040	0.8	1.7	14.2	
297	rs11150863	rs901064	17	78532321	17	78596040	0.9	1.7	13.5	
298	rs4889863	rs901064	17	78550468	17	78596040	0.7	1.7	20.3	[10], [6]
299	rs7503807	rs901064	17	78591111	17	78596040	1.1	1.7	72.3	[13], [10], [6]
300	rs4890056	rs901064	17	78591211	17	78596040	1.3	1.7	65.3	[10], [6]
301	rs901064	rs8080265	17	78596040	17	78604814	1.7	0.2	22.7	[10], [6]
302	rs901064	rs12939549	17	78596040	17	78611724	1.7	1.7	73.1	[10]
303	rs901064	rs12946972	17	78596040	17	78618922	1.7	1.3	24.5	[10], [6]
304	rs901064	rs9897453	17	78596040	17	78634300	1.7	1.0	24.1	[10], [6]
305	rs901064	rs884204	17	78596040	17	78669248	1.7	2.6	32.0	[10], [6]
306	rs901064	rs9913162	17	78596040	17	78695546	1.7	3.6	32.8	[10], [6]
307	rs901064	rs9896771	17	78596040	17	78703899	1.7	0.9	22.5	[10], [6]
308	rs901064	rs9915378	17	78596040	17	78708418	1.7	2.3	32.4	[10], [6]
309	rs1435188	rs1527436	18	41082592	18	37971767	3.6	3.6	14.2	
310	rs13370227	rs7407082	18	75595652	18	75600114	1.4	1.5	16.1	
311	rs7279935	rs1893382	21	17382916	21	17389011	3.5	0.6	14.9	
312	rs7279935	rs2823549	21	17382916	21	17389741	3.5	0.7	13.9	

Table 6: RA results

7.6 Type 1 Diabetes Results

ID	rs_1	rs_2	chr_1	bp_1	chr_2	bp_2	χ_1^2	χ_2^2	flt_{GSS}	source
1	rs9271850	rs9272346	6	0	6	0	4.4	5.3	11.1	
2	rs199698	rs41515647	1	75636696	1	75647488	0.8	19.7	12.0	
3	rs640874	rs1627391	1	108650358	1	108652144	1.9	4.6	12.7	
4	rs1627391	rs499535	1	108652144	1	108654479	4.6	1.5	12.4	
5	rs1627391	rs601063	1	108652144	1	108655473	4.6	1.7	12.4	
6	rs7525703	rs2077749	1	146649064	1	146652637	4.3	12.4	12.9	
7	rs621793	rs16827732	1	187889323	1	187889812	9.6	2.9	13.7	
8	rs350747	rs350753	2	52868656	2	52873788	6.1	1.3	14.3	
9	rs16849921	rs10197379	2	214061022	2	205912603	18.6	0.0	32.1	[10], [6]
10	rs16849921	rs12694298	2	214061022	2	205913268	18.6	0.2	30.5	[10], [6]
11	rs9811898	rs3773090	3	29500834	3	29609837	2.7	0.1	16.9	[10], [6]
12	rs3773090	rs2168765	3	29609837	3	29663099	0.1	1.4	23.4	[10], [6]
13	rs3773090	rs9818762	3	29609837	3	29680018	0.1	1.3	19.4	[10], [6]
14	rs3773090	rs9832480	3	29609837	3	29680095	0.1	2.5	18.8	[10], [6]
15	rs3773090	rs9832974	3	29609837	3	29680561	0.1	1.2	18.8	[10], [6]
16	rs3773090	rs9833015	3	29609837	3	29680632	0.1	1.9	19.5	[10], [6]
17	rs3773090	rs9838725	3	29609837	3	29681858	0.1	1.2	20.8	[10], [6]
18	rs3773090	rs9858796	3	29609837	3	29691014	0.1	2.3	20.6	[10], [6]
19	rs2420412	rs2420407	4	134348127	4	130103904	7.0	7.1	13.5	
20	rs2438074	rs2438083	6	1269690	6	1277371	3.7	0.1	14.3	[10]

21	rs2438077	rs2438083	6	1272236	6	1277371	3.4	0.1	17.3	[6]
22	rs2496292	rs2438083	6	1274617	6	1277371	6.8	0.1	19.2	
23	rs2438083	rs977674	6	1277371	6	1277702	0.1	7.5	25.2	
24	rs2438083	rs977673	6	1277371	6	1277715	0.1	7.7	25.4	
25	rs3129768	rs9272346	6	0	6	0	1.1	5.3	15.0	[6]
26	rs3129768	rs9272723	6	0	6	0	1.1	7.5	15.2	[6]
27	rs7774418	rs2655693	6	80234010	6	80248326	0.6	0.3	11.7	
28	rs2655694	rs2655693	6	80248295	6	80248326	0.2	0.3	16.6	[10]
29	rs2655693	rs7775536	6	80248326	6	80264812	0.3	0.3	11.7	
30	rs1639044	rs1724932	7	2714335	7	2714357	1.9	1.1	15.4	
31	rs6592988	rs12673016	7	52281433	7	52286230	1.1	1.1	13.6	[10]
32	rs6592988	rs4523204	7	52281433	7	52287973	1.1	0.9	13.6	[10]
33	rs691184	rs12706898	7	129197408	7	129254997	0.7	17.5	13.2	[10], [6]
34	rs11526287	rs2250603	7	135297419	7	135315708	0.2	2.7	11.4	
35	rs1809006	rs4840393	8	8961017	8	8962666	2.5	0.4	13.2	
36	rs10099080	rs11984645	8	50357376	8	55069305	0.8	50.4	18.7	[10], [6]
37	rs6473901	rs11984645	8	50386264	8	55069305	1.4	50.4	18.4	[10], [6]
38	rs6473903	rs11984645	8	50388147	8	55069305	0.0	50.4	18.0	[10], [6]
39	rs6983650	rs11984645	8	50406226	8	55069305	0.4	50.4	22.7	[10], [6]
40	rs7844299	rs11984645	8	50430790	8	55069305	0.9	50.4	22.7	[10], [6]
41	rs16919782	rs11984645	8	55063437	8	55069305	3.9	50.4	11.4	
42	rs16919784	rs11984645	8	55063538	8	55069305	3.0	50.4	14.5	[10]
43	rs11991952	rs11984645	8	55069061	8	55069305	4.0	50.4	11.6	
44	rs11984645	rs4737503	8	55069305	8	55071319	50.4	2.7	14.6	[10], [6]
45	rs2919408	rs11782342	8	73739252	8	69281738	5.1	14.0	15.0	[6]
46	rs4571768	rs11782342	8	73743348	8	69281738	2.2	14.0	12.4	
47	rs4307385	rs11782342	8	73743688	8	69281738	2.4	14.0	11.7	
48	rs4307386	rs11782342	8	73743751	8	69281738	2.1	14.0	11.7	
49	rs11780806	rs11782342	8	73771329	8	69281738	0.4	14.0	15.2	
50	rs11782342	rs10096807	8	69281738	8	69283209	14.0	2.0	18.3	[6]
51	rs11782342	rs6994225	8	69281738	8	69299056	14.0	0.0	12.0	
52	rs1833226	rs1420500	8	132701504	8	132701563	0.8	0.7	13.1	
53	rs1420500	rs529894	8	132701563	8	132718006	0.7	5.3	13.1	
54	rs3852458	rs10764038	10	19767401	10	19769651	4.5	2.4	23.2	
55	rs3904887	rs10764038	10	19768895	10	19769651	4.5	2.4	22.1	
56	rs6481942	rs10764038	10	19769118	10	19769651	4.6	2.4	23.2	
57	rs10764038	rs3852459	10	19769651	10	19770716	2.4	4.4	23.6	
58	rs12765184	rs12573160	10	89510229	10	89540888	2.8	1.9	14.3	
59	rs12775041	rs12573160	10	89515818	10	89540888	2.8	1.9	15.5	
60	rs9664653	rs12573160	10	89525860	10	89540888	2.7	1.9	14.6	
61	rs12573160	rs11202557	10	89540888	10	89552283	1.9	2.7	14.6	
62	rs12573160	rs11816798	10	89540888	10	89555714	1.9	2.6	14.3	
63	rs12573160	rs12781171	10	89540888	10	89565700	1.9	2.7	14.6	
64	rs12573160	rs12762731	10	89540888	10	89579646	1.9	2.7	14.7	
65	rs7395011	rs10791991	11	68471479	11	68484983	0.5	1.3	17.0	
66	rs4628676	rs10791991	11	68482230	11	68484983	0.3	1.3	17.0	
67	rs10791991	rs7101673	11	68484983	11	68489300	1.3	0.5	17.0	
68	rs2196519	rs12790605	11	91020012	11	91024468	7.5	5.0	12.7	
69	rs2196519	rs12791202	11	91020012	11	91024722	7.5	5.7	12.0	
70	rs2196519	rs1436623	11	91020012	11	91024970	7.5	4.6	13.3	
71	rs7152833	rs4144189	14	21861211	14	21918876	3.6	2.3	11.4	
72	rs11624794	rs1958305	14	24260146	14	24273124	11.2	33.4	14.2	[6]
73	rs1958305	rs17184408	14	24273124	14	24282020	33.4	0.5	20.7	[10], [6]
74	rs1958305	rs12100601	14	24273124	14	24284173	33.4	1.9	20.0	[6]
75	rs11849674	rs7154773	14	60688023	14	40912999	4.7	2.5	24.2	[10]

76	rs10148587	rs7154773	14	60689160	14	40912999	4.2	2.5	21.2	[10]
77	rs188620	rs7154773	14	40853319	14	40912999	0.8	2.5	20.2	[10], [6]
78	rs10137732	rs7154773	14	60689209	14	40912999	4.4	2.5	27.2	[10], [6]
79	rs6573298	rs7154773	14	40869612	14	40912999	2.1	2.5	18.0	[10]
80	rs7145505	rs7154773	14	40869718	14	40912999	1.6	2.5	18.3	[10]
81	rs8019531	rs7154773	14	40871554	14	40912999	3.1	2.5	35.4	[6]
82	rs11628587	rs7154773	14	40871574	14	40912999	1.8	2.5	19.3	[10]
83	rs11628628	rs7154773	14	40871689	14	40912999	2.0	2.5	19.0	[10]
84	rs8011227	rs7154773	14	40882151	14	40912999	1.9	2.5	34.6	[10]
85	rs7158657	rs7154773	14	40888940	14	40912999	2.9	2.5	33.7	
86	rs10142834	rs7154773	14	40898846	14	40912999	3.0	2.5	35.2	
87	rs17097262	rs7154773	14	40902242	14	40912999	1.0	2.5	34.3	[10], [6]
88	rs1887103	rs7154773	14	40907104	14	40912999	3.1	2.5	29.2	
89	rs7154773	rs8012816	14	40912999	14	40914246	2.5	1.1	27.6	[10]
90	rs7154773	rs10130695	14	40912999	14	40919869	2.5	1.3	34.3	[10], [6]
91	rs7154773	rs1998225	14	40912999	14	40941373	2.5	1.9	17.2	
92	rs7154773	rs8004280	14	40912999	14	60780429	2.5	2.2	17.0	
93	rs7154773	rs1951116	14	40912999	14	40946079	2.5	1.9	14.6	
94	rs7154773	rs1951117	14	40912999	14	60782214	2.5	2.0	17.2	
95	rs2757527	rs2757528	14	100660933	14	100661077	1.2	0.0	13.4	
96	rs2757527	rs2766696	14	100660933	14	100661320	1.2	0.1	14.2	
97	rs17478618	rs17479003	15	26568722	15	26577328	0.7	1.7	13.1	
98	rs17478618	rs17400706	15	26568722	15	26579253	0.7	1.4	13.1	
99	rs6016703	rs7262414	20	37529966	20	37549074	0.8	5.9	14.1	
100	rs6030019	rs7262414	20	37530026	20	37549074	0.4	5.9	14.2	
101	rs2223424	rs7262414	20	37539550	20	37549074	3.1	5.9	15.0	
102	rs7262414	rs6072650	20	37549074	20	37560066	5.9	3.1	12.9	
103	rs7262414	rs6102728	20	37549074	20	37570311	5.9	2.9	14.7	
104	rs7262414	rs2867064	20	37549074	20	37582450	5.9	3.3	14.7	
105	rs7262414	rs6072667	20	37549074	20	37606093	5.9	3.2	15.0	
106	rs7262414	rs10485689	20	37549074	20	40879061	5.9	3.7	14.7	
107	rs2823543	rs2404032	21	17375633	21	17376332	1.9	1.2	17.0	

Table 7: T1D results

7.7 Type 2 Diabetes Results

ID	rs_1	rs_2	chr_1	bp_1	chr_2	bp_2	$\chi2_1$	$\chi2_2$	$fltGSS$	source
1	rs10495652	rs12472889	2	17139289	2	17161082	0.1	0.2	14.8	[10], [6]
2	rs12620194	rs12472889	2	17148004	2	17161082	0.3	0.2	14.6	[10], [6]
3	rs6809441	rs33916626	3	41494605	3	41539388	2.7	0.5	11.1	[10], [6]
4	rs2073108	rs17101278	1	54839069	1	54840496	9.8	2.0	13.3	
5	rs4598448	rs13373826	1	75963934	1	75970795	0.8	0.5	14.2	
6	rs2209307	rs11587221	1	89723344	1	89729950	7.8	1.0	23.1	[10]
7	rs6531271	rs12472889	2	17139933	2	17161082	0.0	0.2	16.9	[10], [6]
8	rs16849921	rs10197379	2	214061022	2	205912603	18.6	0.0	30.5	[10], [6]
9	rs16849921	rs12694298	2	214061022	2	205913268	18.6	0.2	31.5	[10], [6]
10	rs156820	rs266404	3	6469083	3	6481514	0.8	4.4	13.1	
11	rs266403	rs266404	3	6481002	3	6481514	1.0	4.4	14.1	
12	rs6777476	rs1520050	3	20455900	3	20500104	2.2	13.3	15.2	
13	rs939298	rs1520050	3	20477076	3	20500104	2.1	13.3	19.7	[10]
14	rs9859908	rs1520050	3	20489086	3	20500104	2.8	13.3	19.2	[10]

15	rs4683464	rs295470	3	139196961	3	139213903	5.7	18.9	13.2	
16	rs176983	rs295470	3	139213755	3	139213903	5.6	18.9	13.1	
17	rs295470	rs167187	3	139213903	3	139237008	18.9	5.9	13.2	
18	rs959880	rs2314349	3	183088613	3	183091098	5.2	7.8	17.2	[10]
19	rs2314349	rs906719	3	183091098	3	183091144	7.8	4.6	16.9	[10]
20	rs2314349	rs2314348	3	183091098	3	183091330	7.8	5.4	17.2	[10]
21	rs2314349	rs2089588	3	183091098	3	183091474	7.8	5.2	17.5	[10]
22	rs6826705	rs6853712	4	1122634	4	1126327	0.6	2.2	22.9	[10], [6]
23	rs6826705	rs2127908	4	1122634	4	1128925	0.6	0.2	23.1	[10]
24	rs6826705	rs4974627	4	1122634	4	1129975	0.6	0.5	22.2	[10]
25	rs13149206	rs17547815	4	37140462	4	37140506	23.3	6.4	13.3	
26	rs13149206	rs12502592	4	37140462	4	37140633	23.3	6.7	13.2	
27	rs343197	rs1450463	4	121798485	4	121893165	3.3	5.1	12.9	[6]
28	rs1450472	rs1450463	4	121803292	4	121893165	2.9	5.1	12.9	[6]
29	rs2667195	rs1450463	4	121807690	4	121893165	2.0	5.1	16.0	[6]
30	rs343170	rs1450463	4	121811427	4	121893165	2.1	5.1	15.2	[6]
31	rs343168	rs1450463	4	121816501	4	121893165	2.2	5.1	16.0	[6]
32	rs1450466	rs1450463	4	121892782	4	121893165	0.8	5.1	30.3	[6]
33	rs1450465	rs1450463	4	121892799	4	121893165	1.8	5.1	30.7	[6]
34	rs1450463	rs12650193	4	121893165	4	121900882	5.1	7.0	30.2	[6]
35	rs1872292	rs6831744	4	143232882	4	143233187	2.4	2.7	13.2	
36	rs1173196	rs1173198	5	4816811	5	4818616	6.6	2.5	16.2	
37	rs1173196	rs816471	5	4816811	5	4828722	6.6	2.7	16.2	
38	rs1173196	rs816478	5	4816811	5	4833867	6.6	2.1	15.9	
39	rs17287085	rs7700706	5	141284815	5	141294362	2.7	17.9	14.8	[6]
40	rs7700706	rs6580205	5	141294362	5	141294691	17.9	4.1	14.8	
41	rs7700706	rs758462	5	141294362	5	141297178	17.9	5.0	14.8	
42	rs2655694	rs2655693	6	80248295	6	80248326	0.2	0.3	14.8	
43	rs2655693	rs2655688	6	80248326	6	80260073	0.3	1.7	13.3	
44	rs2679692	rs2684266	6	119938231	6	119938301	0.4	5.4	13.1	
45	rs2679692	rs1357056	6	119938231	6	119940718	0.4	5.3	12.7	
46	rs2679692	rs2684264	6	119938231	6	119942010	0.4	4.6	12.6	
47	rs10235699	rs4308634	7	46919177	7	46887213	0.5	1.8	13.7	[6]
48	rs7791862	rs7792409	7	47743256	7	47743362	1.4	1.1	21.8	[10]
49	rs7791862	rs7792551	7	47743256	7	47743434	1.4	1.2	21.0	[10]
50	rs7791862	rs7792432	7	47743256	7	47743485	1.4	1.2	21.8	[10]
51	rs10807711	rs10950088	7	67247690	7	67264560	0.1	2.6	12.9	
52	rs10950088	rs11773150	7	67264560	7	67264811	2.6	0.8	13.7	
53	rs10950088	rs6961794	7	67264560	7	67265709	2.6	0.1	14.6	
54	rs10950088	rs10755834	7	67264560	7	67267349	2.6	0.1	14.4	
55	rs10950088	rs2865111	7	67264560	7	67269597	2.6	0.2	12.9	
56	rs1513921	rs4730350	7	108606430	7	108607230	8.6	4.1	15.6	
57	rs11526287	rs2250603	7	135297419	7	135315708	0.2	2.7	12.1	
58	rs10253608	rs10266006	7	158469060	7	158474325	2.3	2.0	13.0	
59	rs12113120	rs10266006	7	158470080	7	158474325	2.2	2.0	12.2	
60	rs10949739	rs10266006	7	158472082	7	158474325	2.5	2.0	12.4	
61	rs4909259	rs10266006	7	158473900	7	158474325	2.2	2.0	11.8	
62	rs10266006	rs10237585	7	158474325	7	158474373	2.0	2.0	11.6	
63	rs10266006	rs3793181	7	158474325	7	158481827	2.0	3.0	13.2	
64	rs10266006	rs6459895	7	158474325	7	158482870	2.0	2.9	12.7	
65	rs10266006	rs12698265	7	158474325	7	158489297	2.0	2.2	12.7	
66	rs10099080	rs11984645	8	50357376	8	55069305	0.8	50.4	14.8	[10], [6]
67	rs6473901	rs11984645	8	50386264	8	55069305	1.4	50.4	14.8	[10], [6]
68	rs6473903	rs11984645	8	50388147	8	55069305	0.0	50.4	12.5	[10], [6]
69	rs6983650	rs11984645	8	50406226	8	55069305	0.4	50.4	16.0	[10], [6]

70	rs7844299	rs11984645	8	50430790	8	55069305	0.9	50.4	16.2	[10], [6]
71	rs16919784	rs11984645	8	55063538	8	55069305	3.0	50.4	13.9	[10]
72	rs11984645	rs4737503	8	55069305	8	55071319	50.4	2.7	17.8	[10], [6]
73	rs11774185	rs13256880	8	109082108	8	109086419	3.1	1.1	14.4	
74	rs6421008	rs7827545	8	135512645	8	135566567	1.5	0.6	38.5	[10], [6]
75	rs7386230	rs7827545	8	135526459	8	135566567	0.1	0.6	18.6	[10], [6]
76	rs6988000	rs7827545	8	135559534	8	135566567	2.6	0.6	16.4	[10]
77	rs6578234	rs7827545	8	135566363	8	135566567	0.6	0.6	46.6	[10]
78	rs7827545	rs1372662	8	135566567	8	135567046	0.6	0.6	48.9	[10]
79	rs7827545	rs6578237	8	135566567	8	135569358	0.6	0.3	45.4	[10]
80	rs7827545	rs7846013	8	135566567	8	135569967	0.6	0.8	45.4	[10], [6]
81	rs7827545	rs12679315	8	135566567	8	135624837	0.6	6.8	18.5	[10], [6]
82	rs7827545	rs16905198	8	135566567	8	135626272	0.6	0.3	15.2	[10], [6]
83	rs4072506	rs13271565	8	139672602	8	139675558	0.2	0.3	15.6	
84	rs17152205	rs41380844	10	12794336	10	12797828	0.4	1.2	12.9	
85	rs661882	rs10829261	10	27484632	10	27775288	3.3	4.0	14.1	
86	rs661882	rs717287	10	27484632	10	27495740	3.3	3.8	13.9	
87	rs6481485	rs7078534	10	28123364	10	28123716	2.6	1.0	14.1	
88	rs2566269	rs2641409	11	4569025	11	4569315	2.2	5.5	13.7	
89	rs201892	rs1486581	11	32682691	11	32723951	2.9	0.1	17.6	
90	rs201892	rs10767965	11	32682691	11	32796092	2.9	0.0	18.0	
91	rs201892	rs10835974	11	32682691	11	32816130	2.9	0.3	13.1	
92	rs564229	rs483271	11	74481318	11	74513026	4.0	15.1	11.3	
93	rs556916	rs483271	11	74485613	11	74513026	5.7	15.1	18.4	[10]
94	rs563473	rs483271	11	74488537	11	74513026	5.0	15.1	18.9	[10]
95	rs517035	rs483271	11	74492776	11	74513026	5.7	15.1	18.4	[10]
96	rs578477	rs483271	11	74508609	11	74513026	5.0	15.1	19.0	[10]
97	rs483271	rs500827	11	74513026	11	74522241	15.1	5.1	18.3	[10]
98	rs483271	rs503855	11	74513026	11	74533452	15.1	4.5	17.9	[10]
99	rs11624794	rs1958305	14	24260146	14	24273124	11.2	33.4	15.1	[10], [6]
100	rs1958305	rs17184408	14	24273124	14	24282020	33.4	0.5	19.9	[10], [6]
101	rs1958305	rs12100601	14	24273124	14	24284173	33.4	1.9	17.8	
102	rs11849674	rs7154773	14	60688023	14	40912999	4.7	2.5	34.1	[10], [6]
103	rs10148587	rs7154773	14	60689160	14	40912999	4.2	2.5	30.5	[10], [6]
104	rs188620	rs7154773	14	40853319	14	40912999	0.8	2.5	27.8	[10], [6]
105	rs10137732	rs7154773	14	60689209	14	40912999	4.4	2.5	37.4	[10], [6]
106	rs6573298	rs7154773	14	40869612	14	40912999	2.1	2.5	22.4	[10]
107	rs7145505	rs7154773	14	40869718	14	40912999	1.6	2.5	22.2	[10]
108	rs8019531	rs7154773	14	40871554	14	40912999	3.1	2.5	32.0	[6]
109	rs11628587	rs7154773	14	40871574	14	40912999	1.8	2.5	22.4	[10]
110	rs11628628	rs7154773	14	40871689	14	40912999	2.0	2.5	22.4	[10]
111	rs8011227	rs7154773	14	40882151	14	40912999	1.9	2.5	44.8	[10]
112	rs7158657	rs7154773	14	40888940	14	40912999	2.9	2.5	32.0	
113	rs10142834	rs7154773	14	40898846	14	40912999	3.0	2.5	32.0	
114	rs17097262	rs7154773	14	40902242	14	40912999	1.0	2.5	42.8	[10], [6]
115	rs1887103	rs7154773	14	40907104	14	40912999	3.1	2.5	24.1	
116	rs7154773	rs8012816	14	40912999	14	40914246	2.5	1.1	34.3	[10]
117	rs7154773	rs10130695	14	40912999	14	40919869	2.5	1.3	42.8	[10], [6]
118	rs7154773	rs1998225	14	40912999	14	40941373	2.5	1.9	18.0	
119	rs7154773	rs8004280	14	40912999	14	60780429	2.5	2.2	17.7	
120	rs7154773	rs1951116	14	40912999	14	40946079	2.5	1.9	16.1	
121	rs7154773	rs1951117	14	40912999	14	60782214	2.5	2.0	18.5	
122	rs7154773	rs7146988	14	40912999	14	60811204	2.5	2.9	17.7	[6]
123	rs12908846	rs16969478	15	39910404	15	39930953	0.6	5.0	11.1	
124	rs746655	rs921535	15	74107677	15	74111343	4.5	9.6	18.6	[10]

125	rs8078303	rs9915259	17	43117813	17	43118177	1.2	0.1	15.7	
126	rs6016703	rs7262414	20	37529966	20	37549074	0.8	5.9	12.6	
127	rs6030019	rs7262414	20	37530026	20	37549074	0.4	5.9	12.8	
128	rs2223424	rs7262414	20	37539550	20	37549074	3.1	5.9	14.8	
129	rs7262414	rs6072650	20	37549074	20	37560066	5.9	3.1	12.4	
130	rs7262414	rs6102728	20	37549074	20	37570311	5.9	2.9	14.2	
131	rs7262414	rs2867064	20	37549074	20	37582450	5.9	3.3	14.8	
132	rs7262414	rs6072667	20	37549074	20	37606093	5.9	3.2	16.6	
133	rs7262414	rs10485689	20	37549074	20	40879061	5.9	3.7	15.7	
134	rs2011703	rs6014572	20	54556388	20	54574544	16.7	1.6	15.8	[6]

Table 8: T2D results

References

1. Krzanowski WJ, Hand DJ: *ROC Curves for Continuous Data*, *Volume 111 of* CRC Monographs on Statistics and Applied Probability. Chapman & Hall/CRC 2009.
2. Agresti A: *An Introduction to Categorical Data Analysis*. Wiley 2007.
3. Wan X, Yang C, Yang Q, Xue H, Fan X, Tang NLS, Yu W: **BOOST: A fast approach to detecting gene-gene interactions in genome-wide case-control studies**. *Am J Hum Genet* 2010, **87**(3):1–4.
4. Hemani G, Theocharidis A, Wei W, Haley C: **EpiGPU: exhaustive pairwise epistasis scans parallelized on consumer level graphics cards.** *Bioinformatics* 2011, **27**(11):1462–1465.
5. Hu X, Liu Q, Zhang Z, Li Z, Wang S, He L, Shi Y: **SHEsisEpi, a GPU-enhanced genome-wide SNP-SNP interaction scanning algorithm, efficiently reveals the risk genetic epistasis in bipolar disorder.** *Cell Res* 2010, **20**(7):854–857.
6. Yung LS, Yang C, Wan X, Yu W: **GBOOST: a GPU-based tool for detecting gene-gene interactions in genome-wide case control studies.** *Bioinformatics* 2011, **27**(9):1309–1310.
7. Kam-Thong T, Pütz B, Karbalai N, Müller-Myhsok B, Borgwardt K: **Epistasis detection on quantitative phenotypes by exhaustive enumeration using GPUs.** *Bioinformatics* 2011, **27**(13):i214–i221.
8. Cordell HJ: **Detecting gene–gene interactions that underlie human diseases**. *Nat Rev Genet* 2009, **10**(6):392–404.
9. Slavin TP, Feng T, Schnell A, Zhu X, Elston RC: **Two-marker association tests yield new disease associations for coronary artery disease and hypertension.** *Hum Genet* 2011, **130**(6):725–733.
10. Liu Y, Xu H, Chen S, Chen X, Zhang Z, Zhu Z, Qin X, Hu L, Zhu J, Zhao GP, Kong X: **Genome-Wide Interaction-Based Association Analysis Identified Multiple New Susceptibility Loci for Common Diseases.** *PLoS Genet* 2011, **7**(3):e1001338.
11. The Wellcome Trust Case-Control Consortium: **Genome-wide association study of 14,000 cases of seven common diseases and 3,000 shared controls.** *Nature* 2007, **447**(7145):661–678.
12. Emily M, Mailund T, Hein J, Schauser L, Schierup MH: **Using biological networks to search for interacting loci in genome-wide association studies.** *Eur J Hum Genet* 2009, **17**(10):1231–1240.
13. Wan X, Yang C, Yang Q, Xue H, Tang NLS, Yu W: **Predictive rule inference for epistatic interaction detection in genome-wide association studies.** *Bioinformatics* 2010, **26**:30–37.
14. Gyenesei A, Moody J, Semple CAM, Haley CS, Wei WH: **High-throughput analysis of epistasis in genome-wide association studies with BiForce.** *Bioinformatics* 2012, **28**(15):1957–1964.
